# Supplementary material for: Sex- and age-specific development of ssRNA virus receptor expression in the human brain
Source: PLoS One. 2026 Oct 29;21(10):e0360526. doi: 10.1371/journal.pone.0360526 (PMC13718946; doi:10.1371/journal.pone.0360526)
Supplement: Supplementary file 1 [file pone.0360526.s001.pdf]

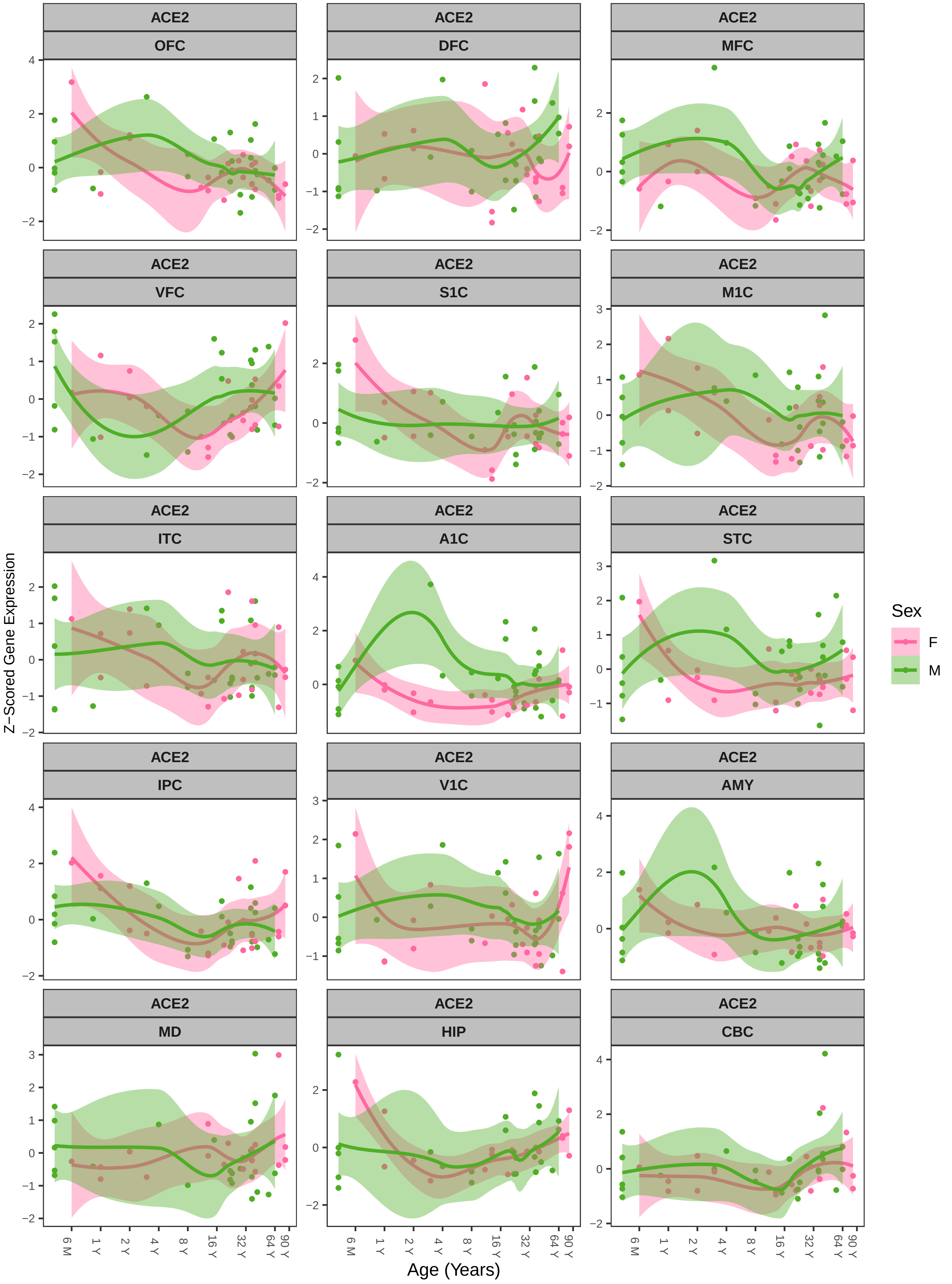
ACE2
OFC
DFC
MFC
VFC
S1C
M1C
ITC
A1C
STC
IPC
V1C
AMY
MD
HIP
CBC
Z−Scored Gene Expression
Age (Years)
6 M
1 Y
2 Y
4 Y
8 Y
16 Y
32 Y
64 Y
90 Y
Sex
F
M

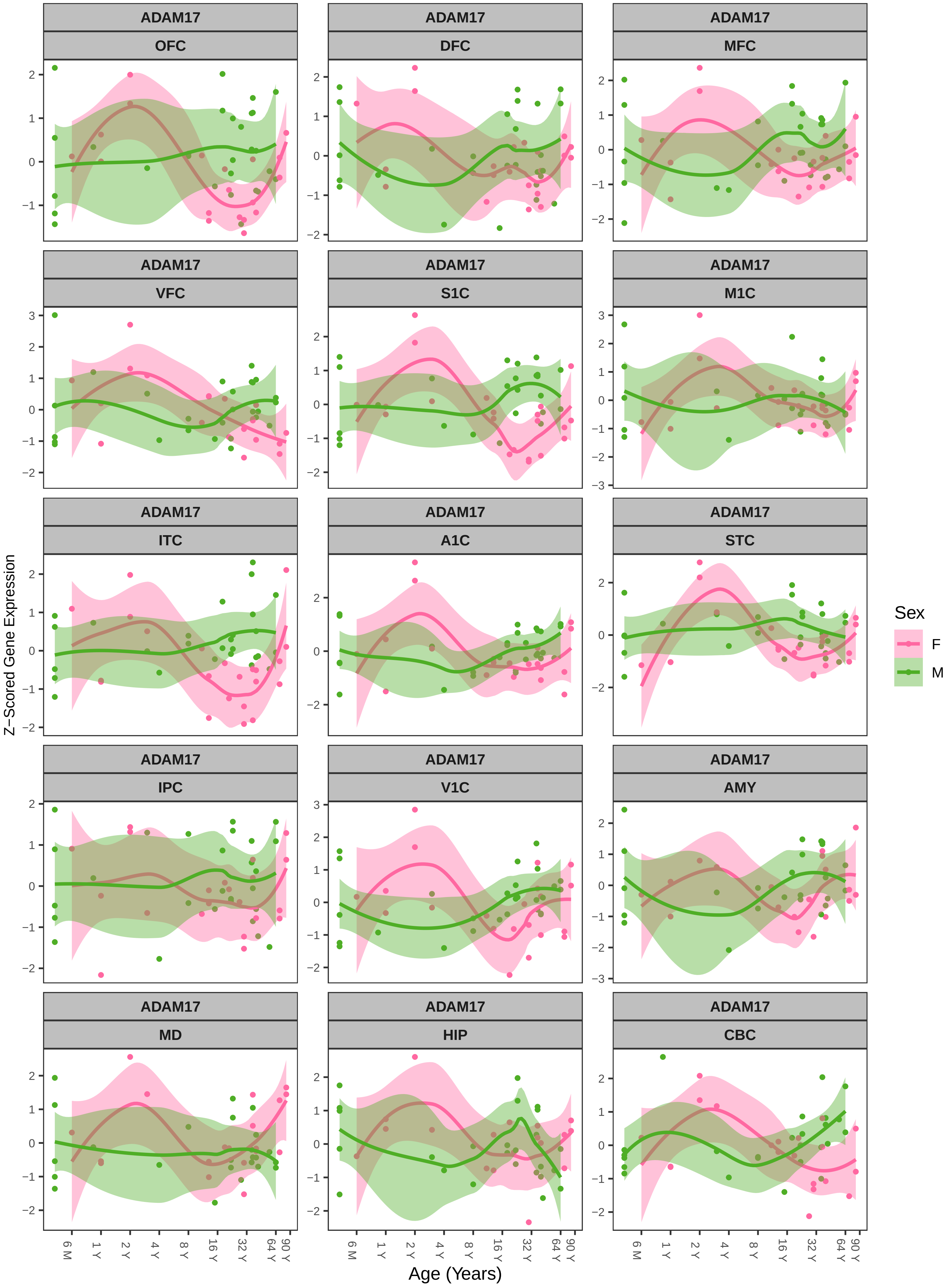

ADAM17
OFC
DFC
MFC
VFC
S1C
M1C
ITC
A1C
STC
IPC
V1C
AMY
MD
HIP
CBC
Z−Scored Gene Expression
Age (Years)
6 M
1 Y
2 Y
4 Y
8 Y
16 Y
32 Y
64 Y
90 Y
Sex
F
M

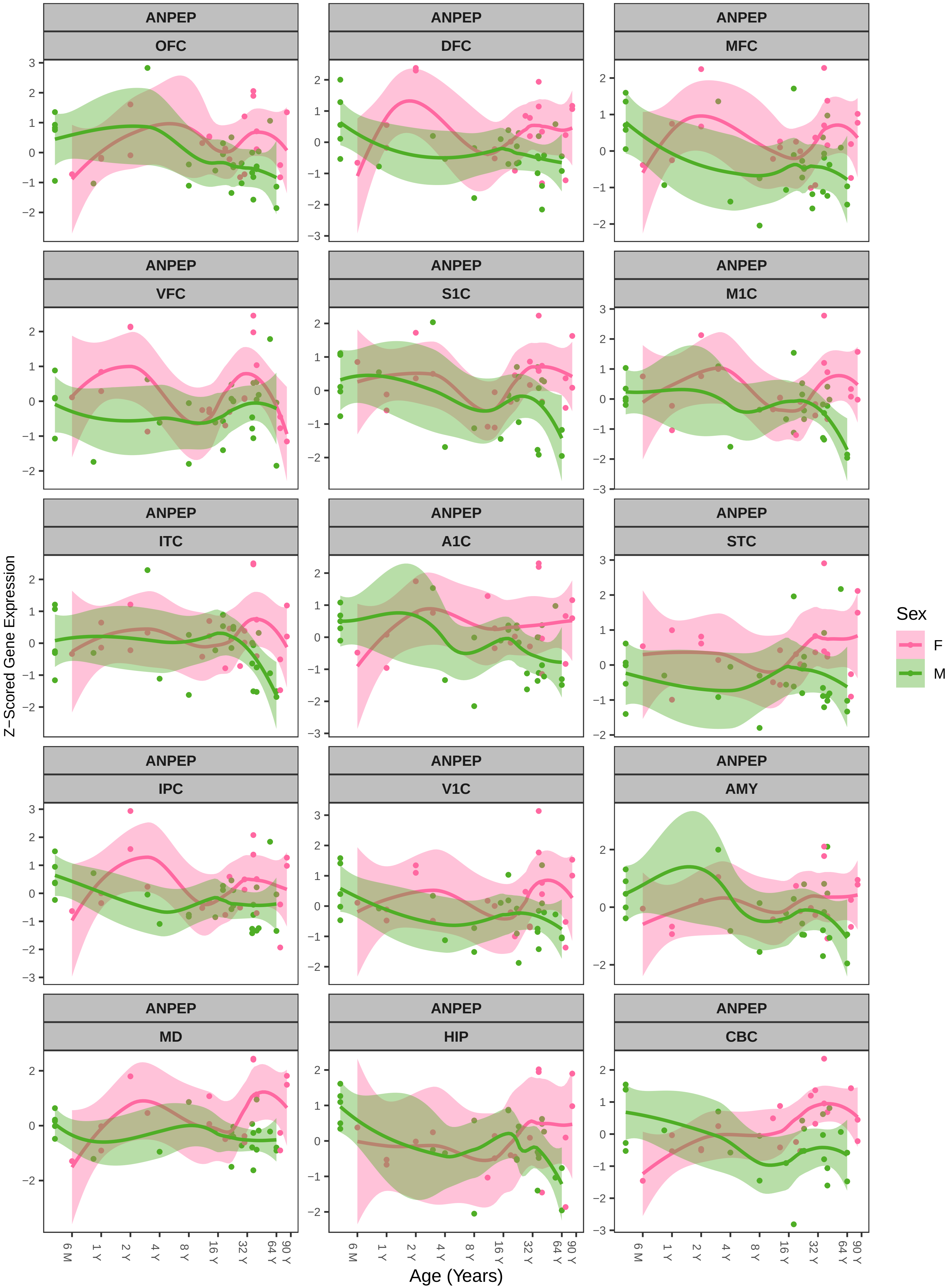

ANPEP
OFC
DFC
MFC
VFC
S1C
M1C
ITC
A1C
STC
IPC
V1C
AMY
MD
HIP
CBC
Z−Scored Gene Expression
Age (Years)
6 M
1 Y
2 Y
4 Y
8 Y
16 Y
32 Y
64 Y
90 Y
Sex
F
M

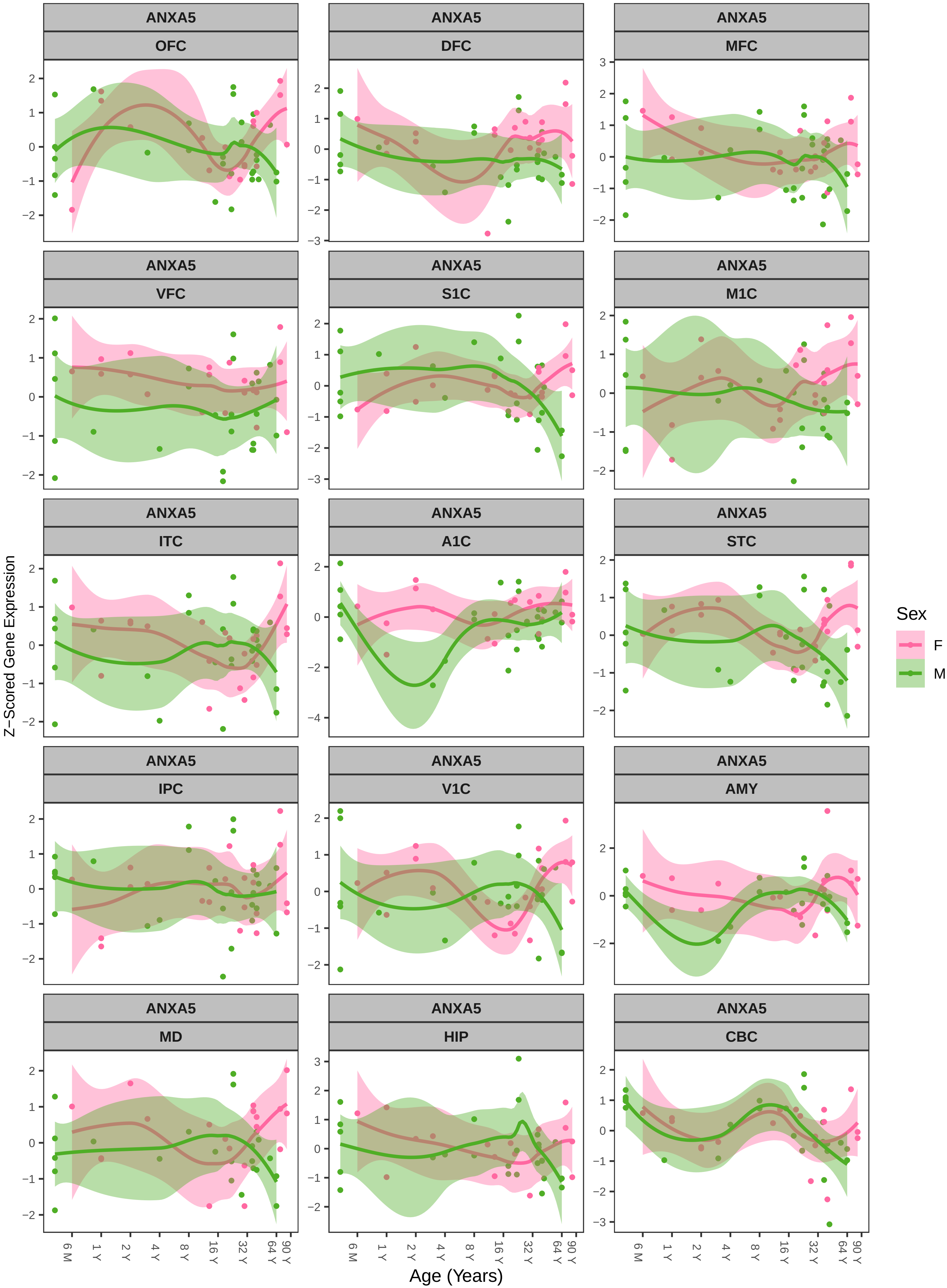
ANXA5
OFC
DFC
MFC
VFC
S1C
M1C
ITC
A1C
STC
IPC
V1C
AMY
MD
HIP
CBC
Z−Scored Gene Expression
Age (Years)
6 M
1 Y
2 Y
4 Y
8 Y
16 Y
32 Y
64 Y
90 Y
Sex
F
M

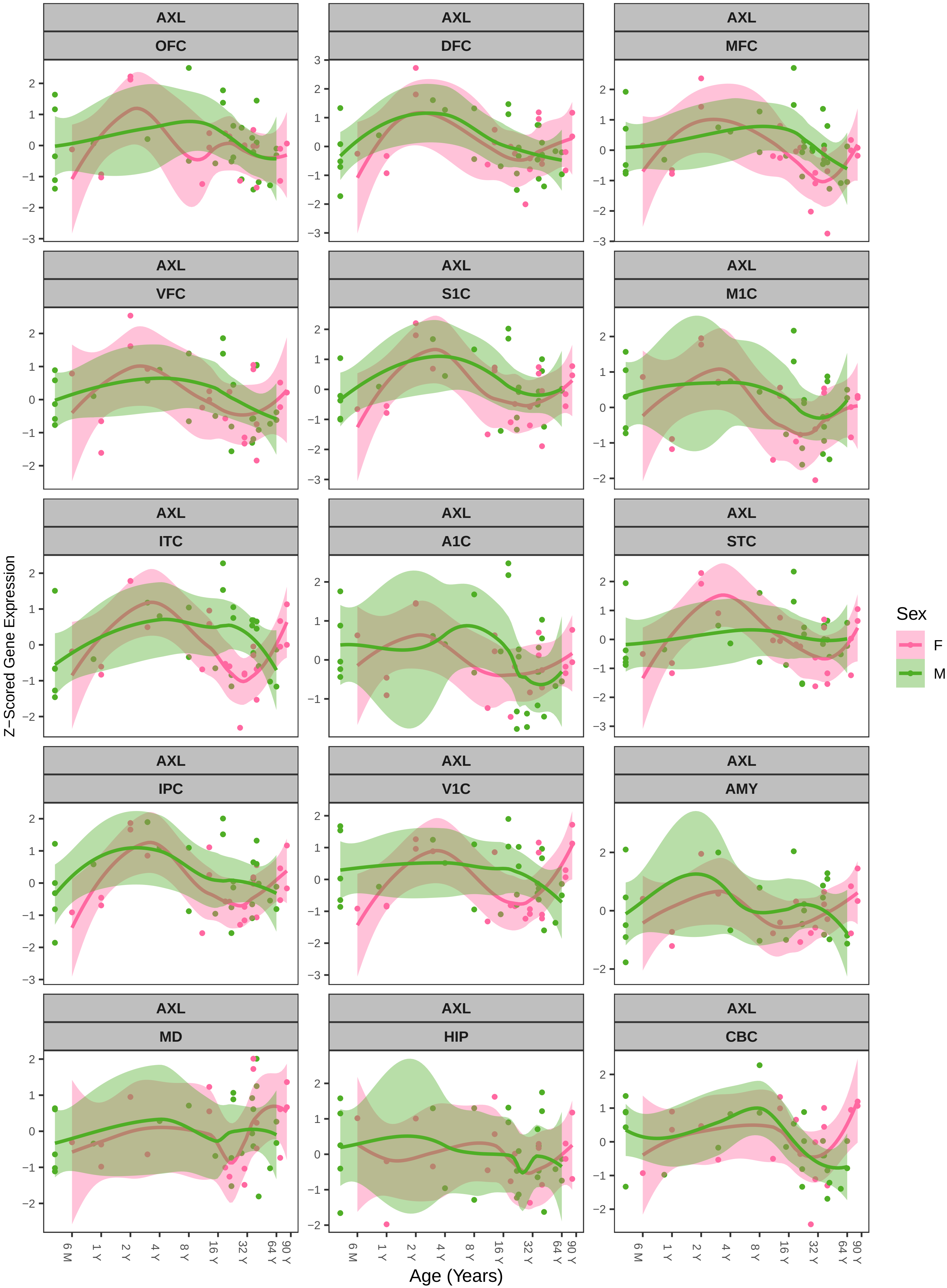
AXL
OFC
DFC
MFC
VFC
S1C
M1C
ITC
A1C
STC
IPC
V1C
AMY
MD
HIP
CBC
Z−Scored Gene Expression
Age (Years)
6 M
1 Y
2 Y
4 Y
8 Y
16 Y
32 Y
64 Y
90 Y
Sex
F
M

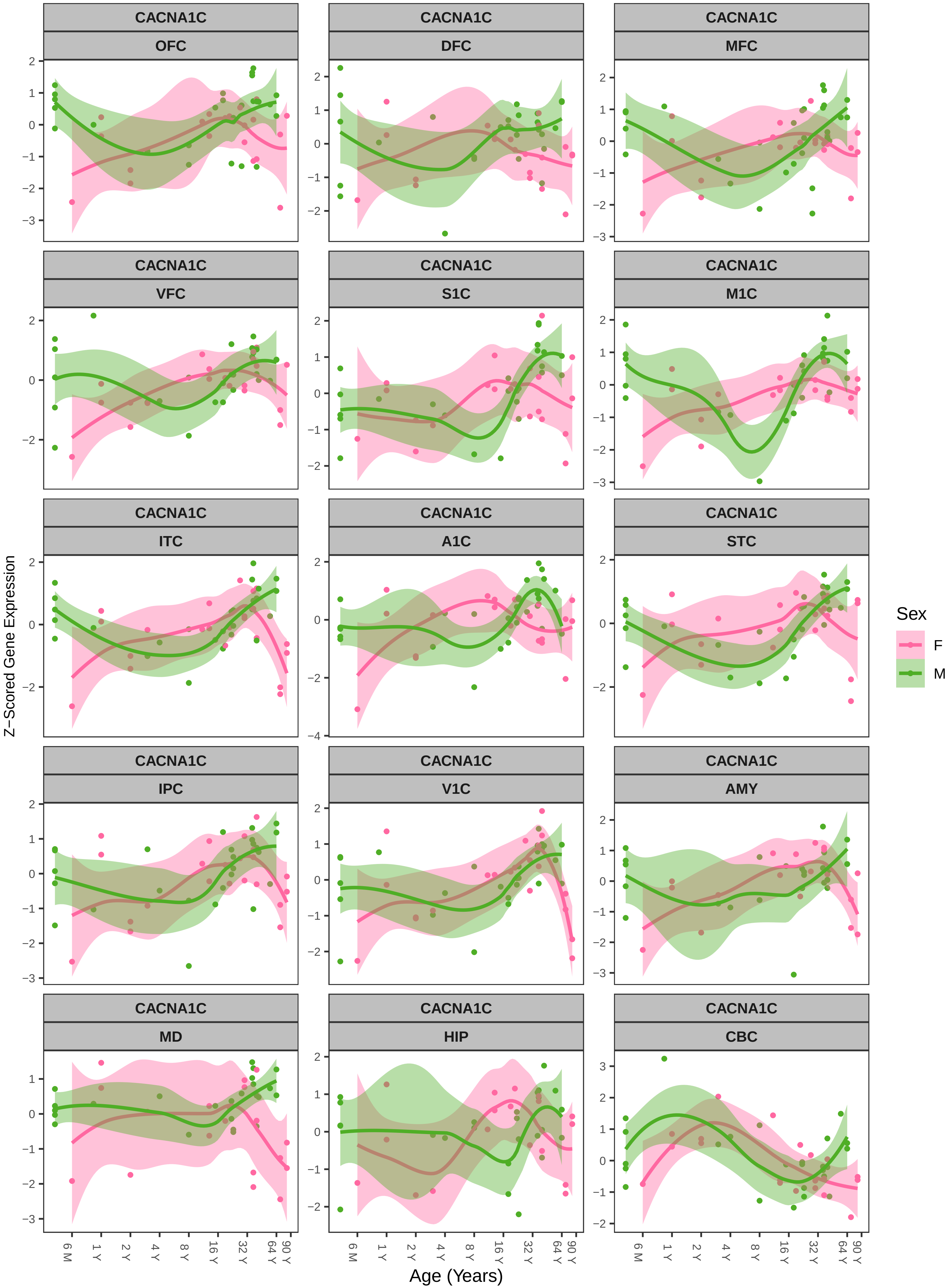
CACNA1C
OFC
DFC
MFC
VFC
S1C
M1C
ITC
A1C
STC
IPC
V1C
AMY
MD
HIP
CBC
Z−Scored Gene Expression
Age (Years)
6 M
1 Y
2 Y
4 Y
8 Y
16 Y
32 Y
64 Y
90 Y
Sex
F
M

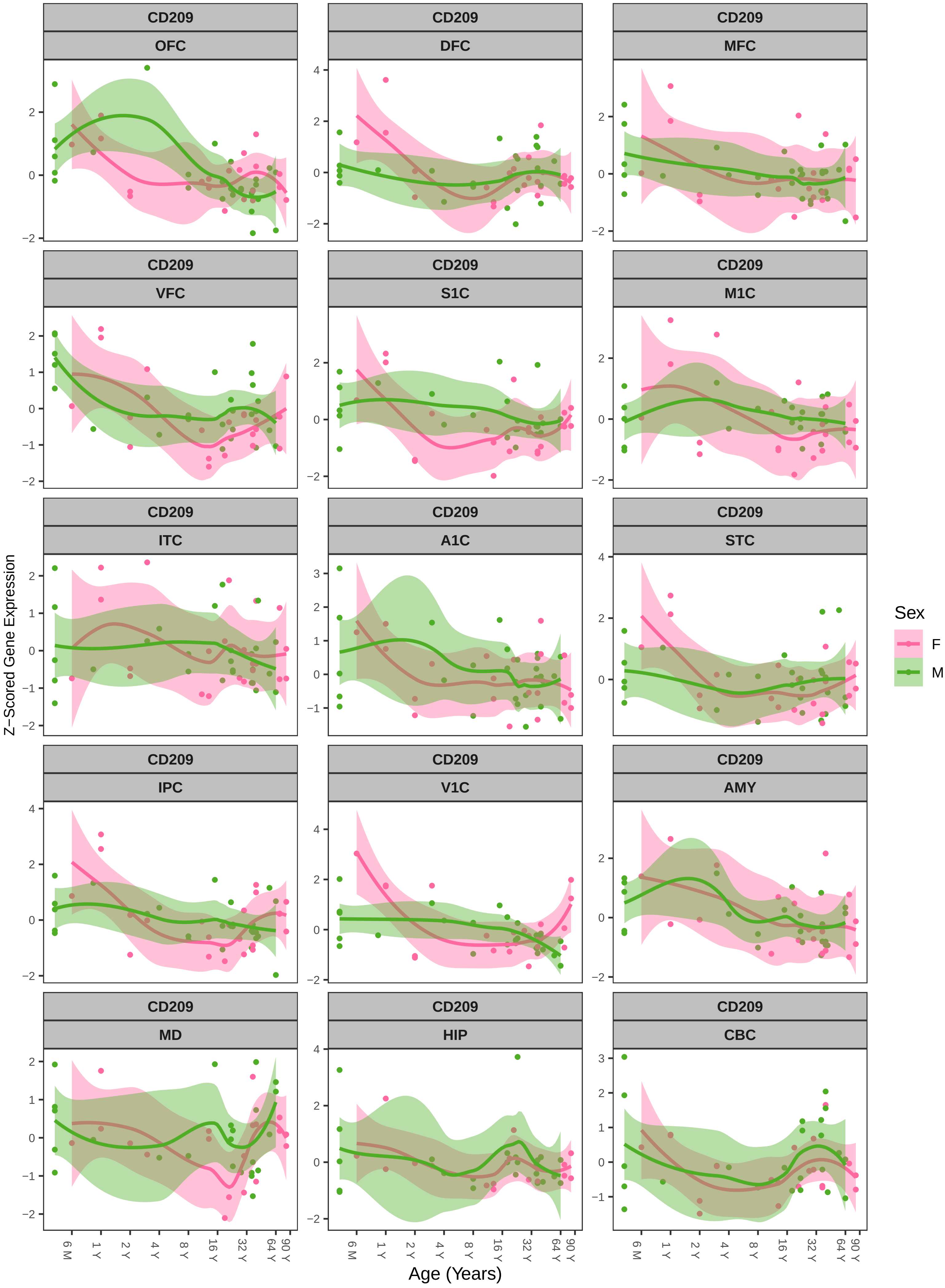
CD209
OFC
DFC
MFC
VFC
S1C
M1C
ITC
A1C
STC
IPC
V1C
AMY
MD
HIP
CBC
Z−Scored Gene Expression
Age (Years)
6 M
1 Y
2 Y
4 Y
8 Y
16 Y
32 Y
64 Y
90 Y
Sex
F
M

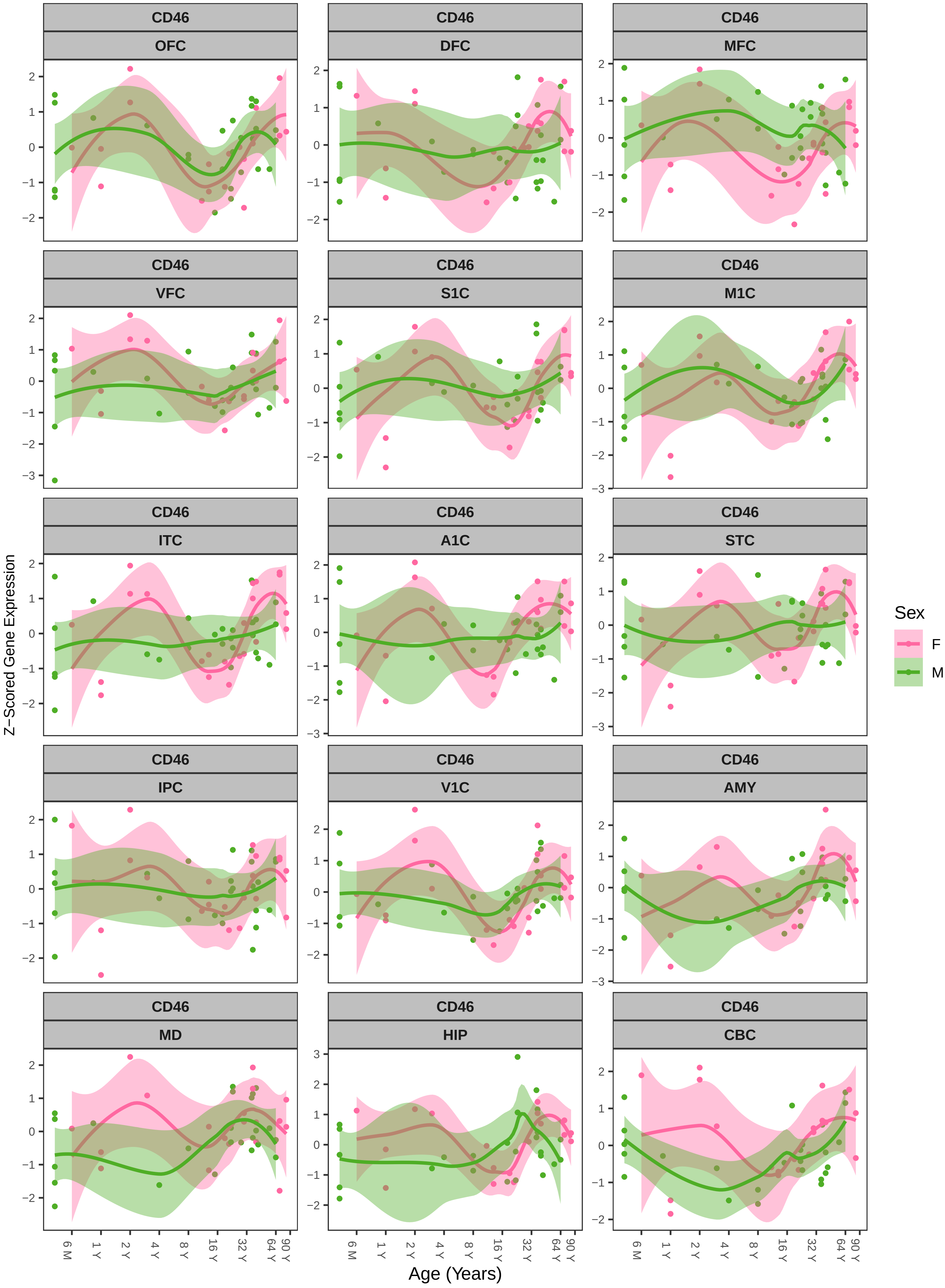
CD46
OFC
DFC
MFC
VFC
S1C
M1C
ITC
A1C
STC
IPC
V1C
AMY
MD
HIP
CBC
Z−Scored Gene Expression
Age (Years)
6 M
1 Y
2 Y
4 Y
8 Y
16 Y
32 Y
64 Y
90 Y
Sex
F
M

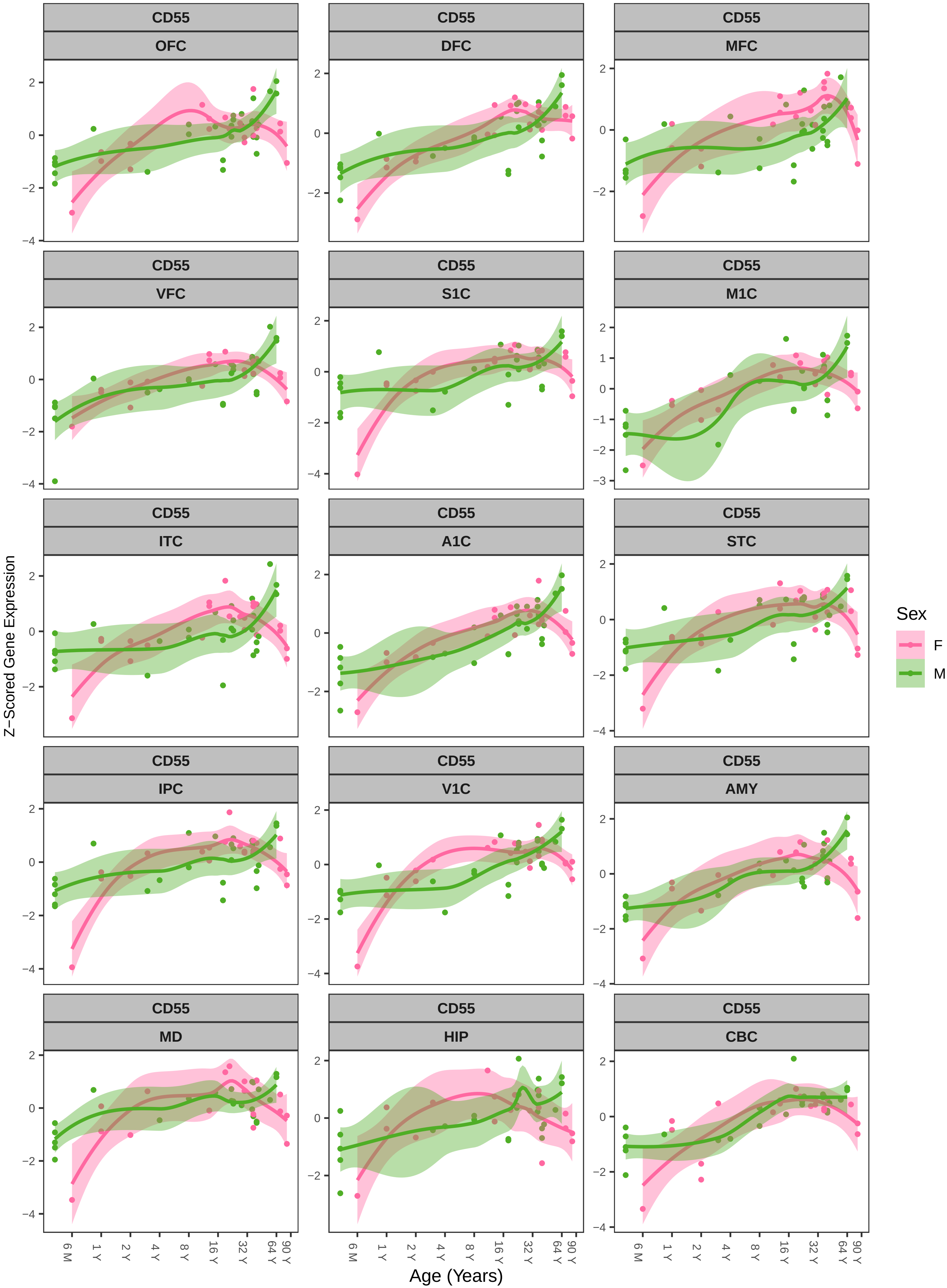
CD55
OFC
DFC
MFC
VFC
S1C
M1C
ITC
A1C
STC
IPC
V1C
AMY
MD
HIP
CBC
Z−Scored Gene Expression
Age (Years)
6 M
1 Y
2 Y
4 Y
8 Y
16 Y
32 Y
64 Y
90 Y
Sex
F
M

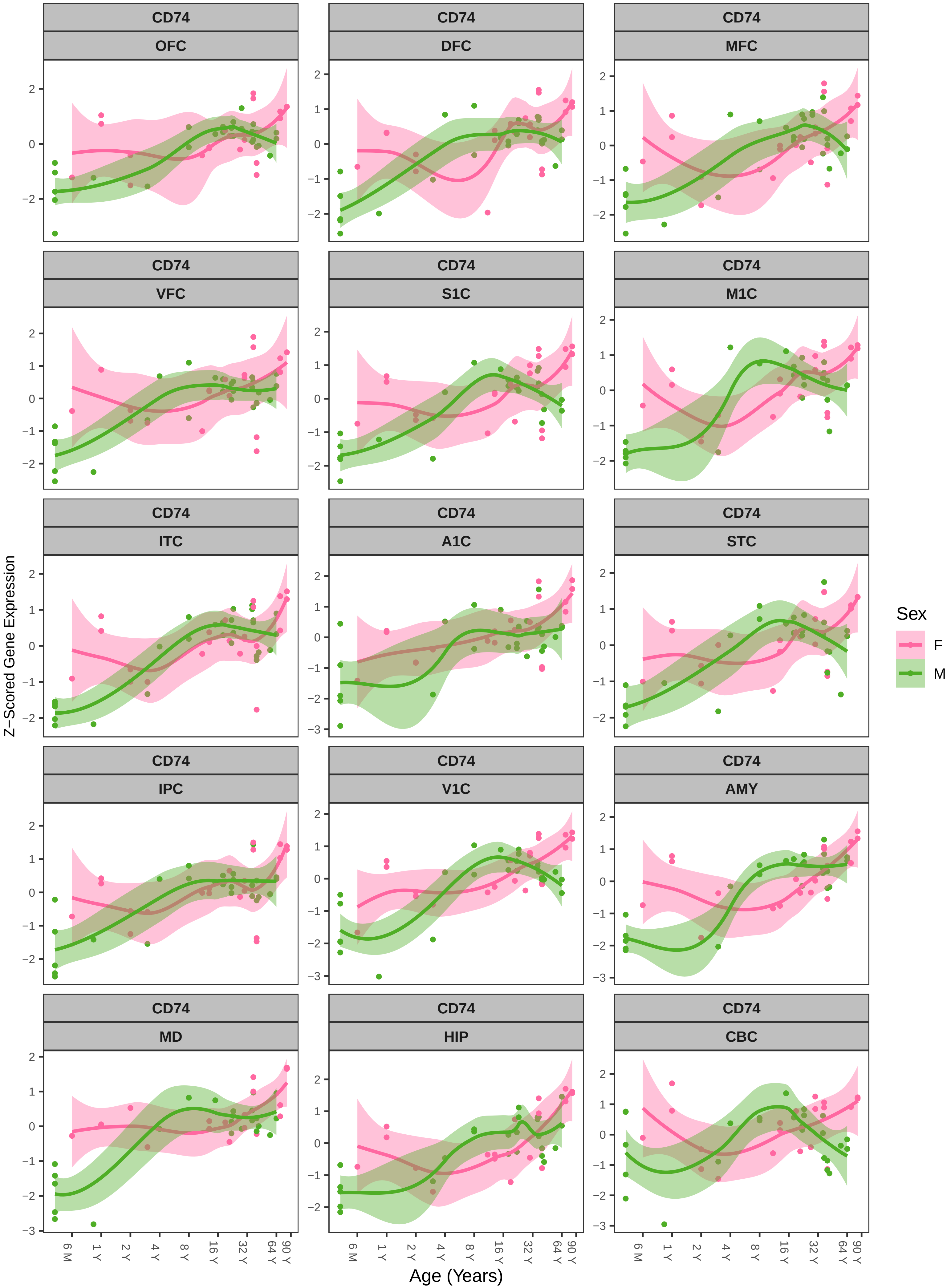
CD74
OFC
DFC
MFC
VFC
S1C
M1C
ITC
A1C
STC
IPC
V1C
AMY
MD
HIP
CBC
Z−Scored Gene Expression
Age (Years)
6 M
1 Y
2 Y
4 Y
8 Y
16 Y
32 Y
64 Y
90 Y
Sex
F
M

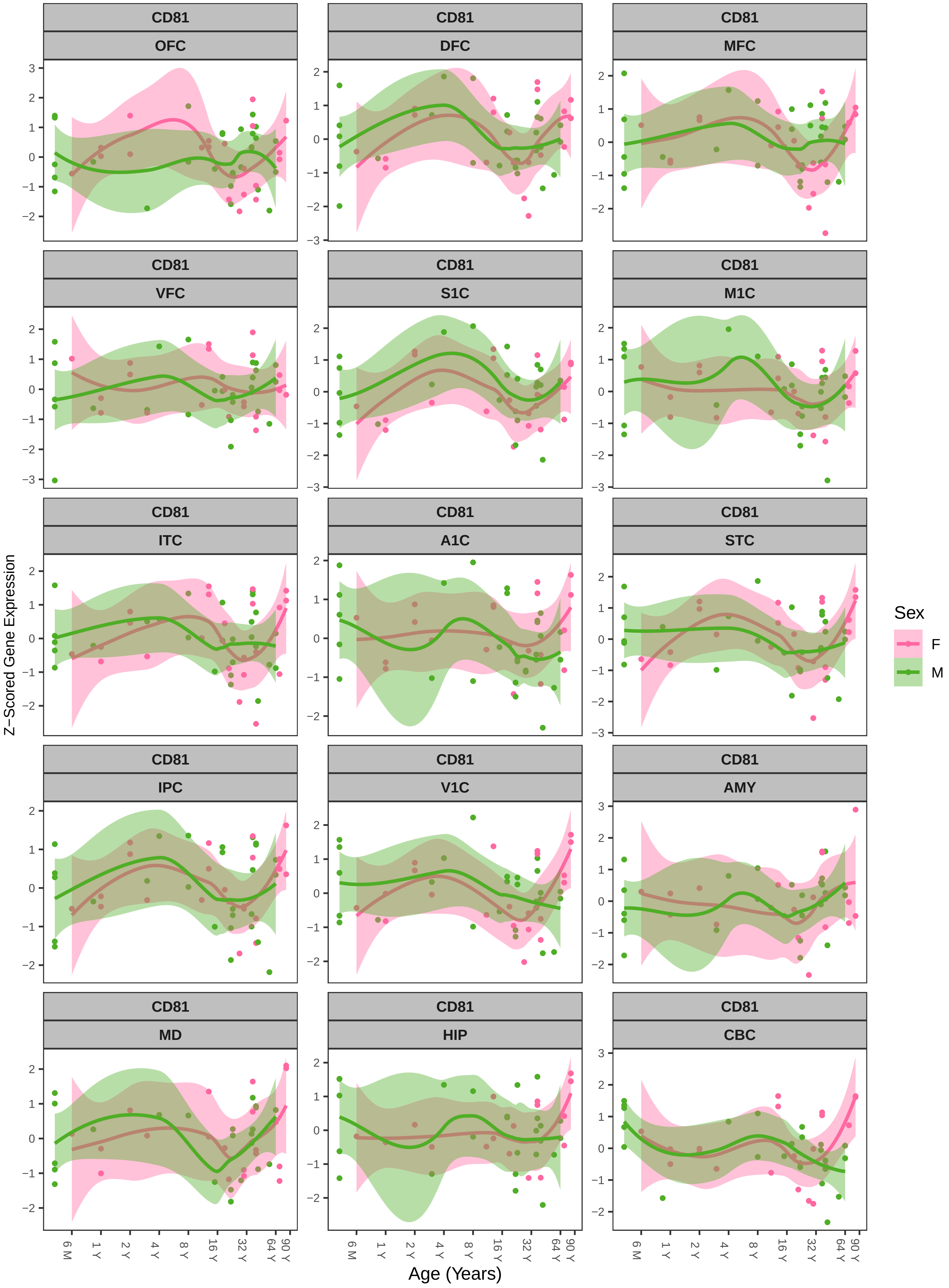
CD81
OFC
DFC
MFC
VFC
S1C
M1C
ITC
A1C
STC
IPC
V1C
AMY
MD
HIP
CBC
Z−Scored Gene Expression
Age (Years)
6 M
1 Y
2 Y
4 Y
8 Y
16 Y
32 Y
64 Y
90 Y
Sex
F
M

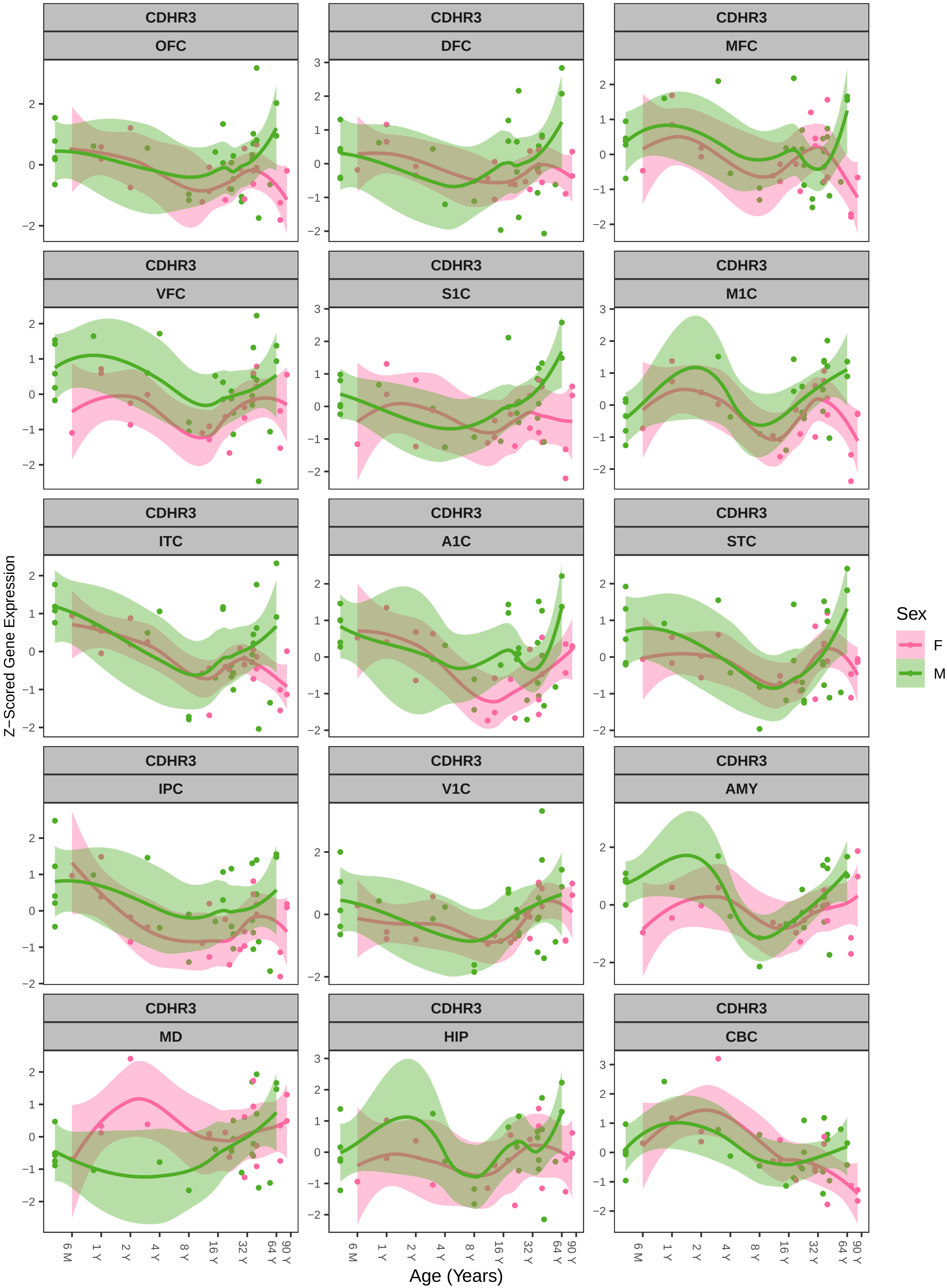

CDHR3
OFC
DFC
MFC
VFC
S1C
M1C
ITC
A1C
STC
IPC
V1C
AMY
MD
HIP
CBC
Z−Scored Gene Expression
Age (Years)
6 M
1 Y
2 Y
4 Y
8 Y
16 Y
32 Y
64 Y
90 Y
Sex
F
M

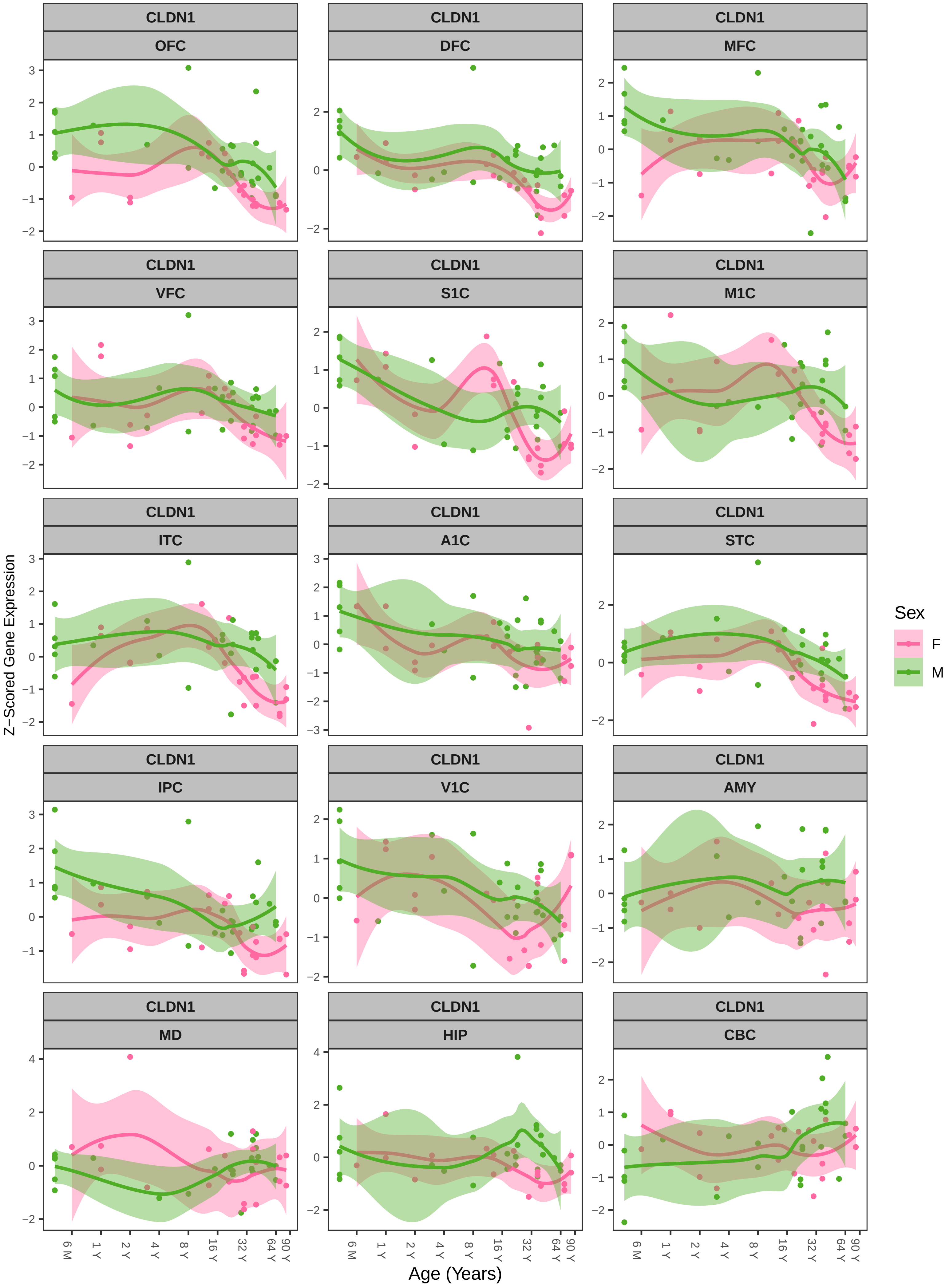
CLDN1
OFC
DFC
MFC
VFC
S1C
M1C
ITC
A1C
STC
IPC
V1C
AMY
MD
HIP
CBC
Z−Scored Gene Expression
Age (Years)
6 M
1 Y
2 Y
4 Y
8 Y
16 Y
32 Y
64 Y
90 Y
Sex
F
M

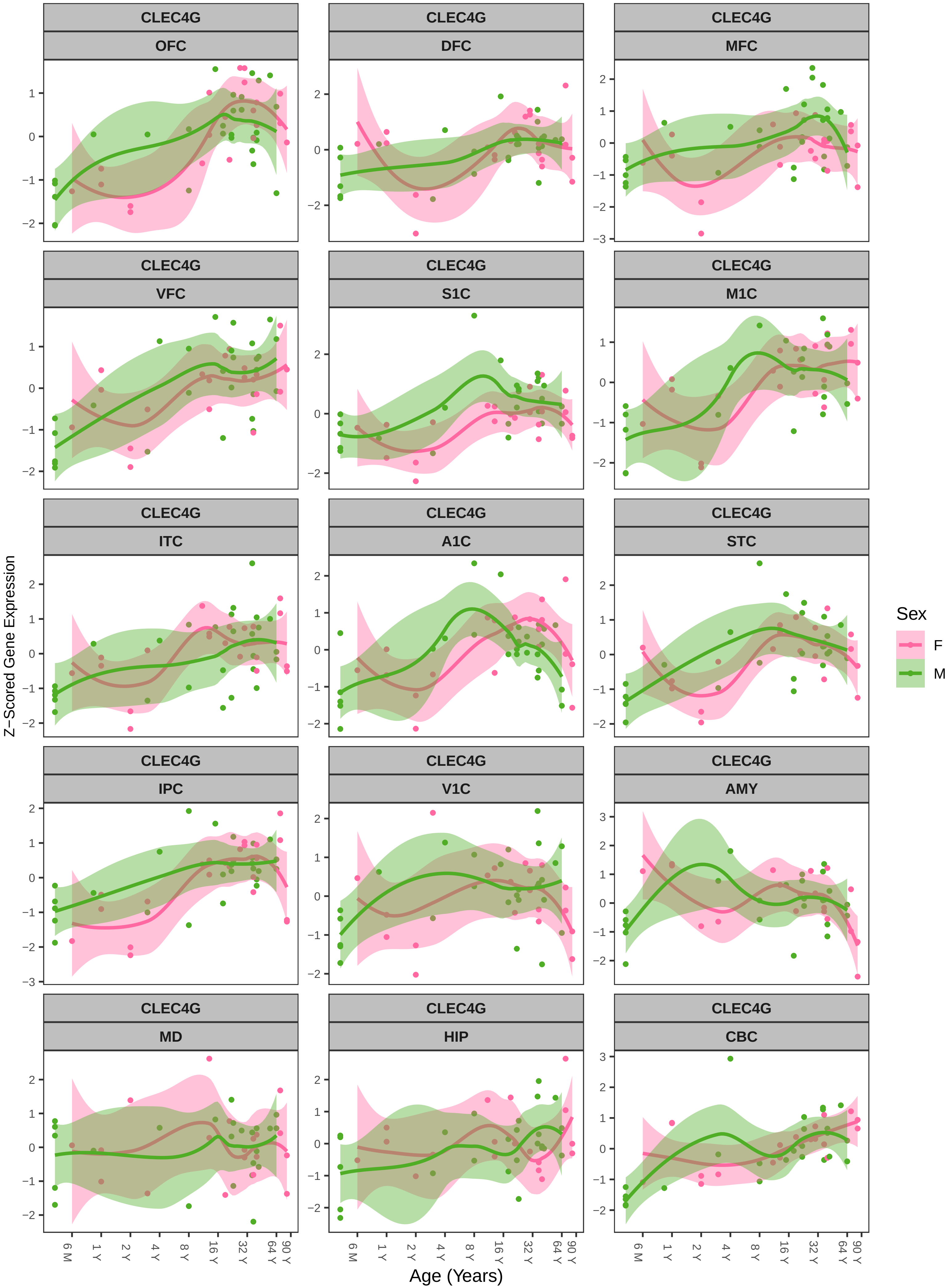
CLEC4G
OFC
DFC
MFC
VFC
S1C
M1C
ITC
A1C
STC
IPC
V1C
AMY
MD
HIP
CBC
Z−Scored Gene Expression
Age (Years)
6 M
1 Y
2 Y
4 Y
8 Y
16 Y
32 Y
64 Y
90 Y
Sex
F
M

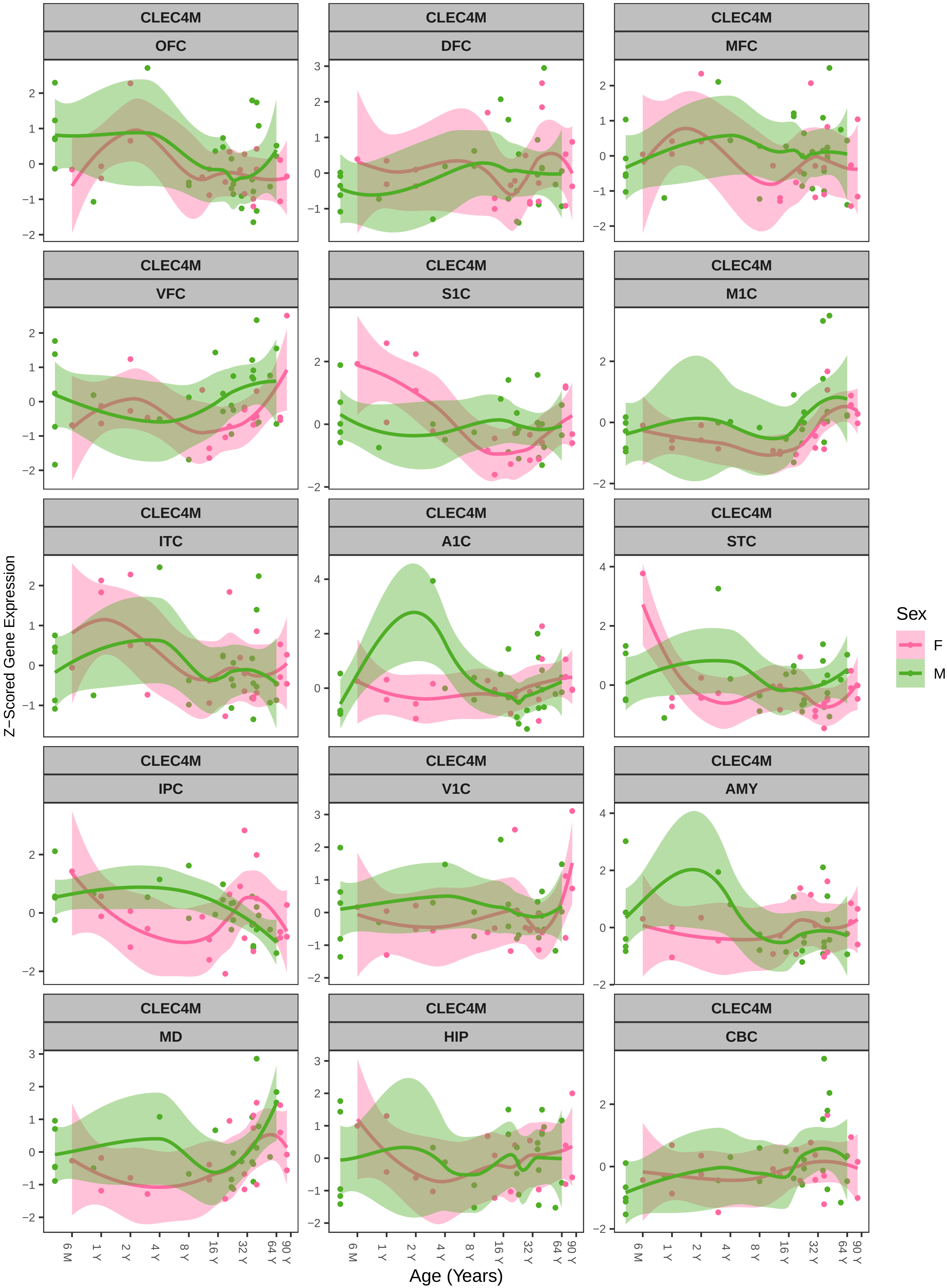
CLEC4M
OFC
DFC
MFC
VFC
S1C
M1C
ITC
A1C
STC
IPC
V1C
AMY
MD
HIP
CBC
Z−Scored Gene Expression
Age (Years)
6 M
1 Y
2 Y
4 Y
8 Y
16 Y
32 Y
64 Y
90 Y
Sex
F
M

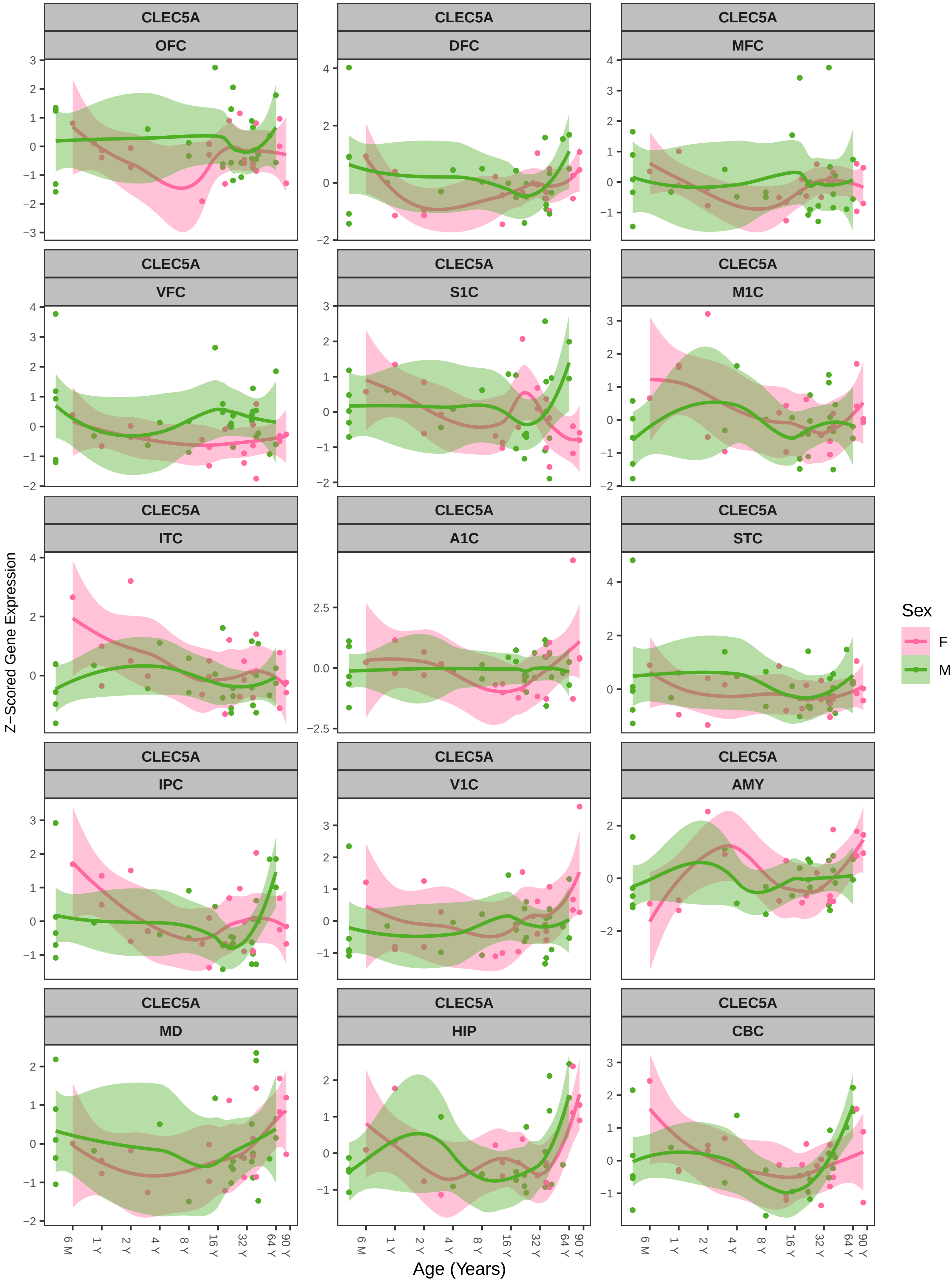
CLEC5A
OFC
DFC
MFC
VFC
S1C
M1C
ITC
A1C
STC
IPC
V1C
AMY
MD
HIP
CBC
Z−Scored Gene Expression
Age (Years)
6 M
1 Y
2 Y
4 Y
8 Y
16 Y
32 Y
64 Y
90 Y
Sex
F
M

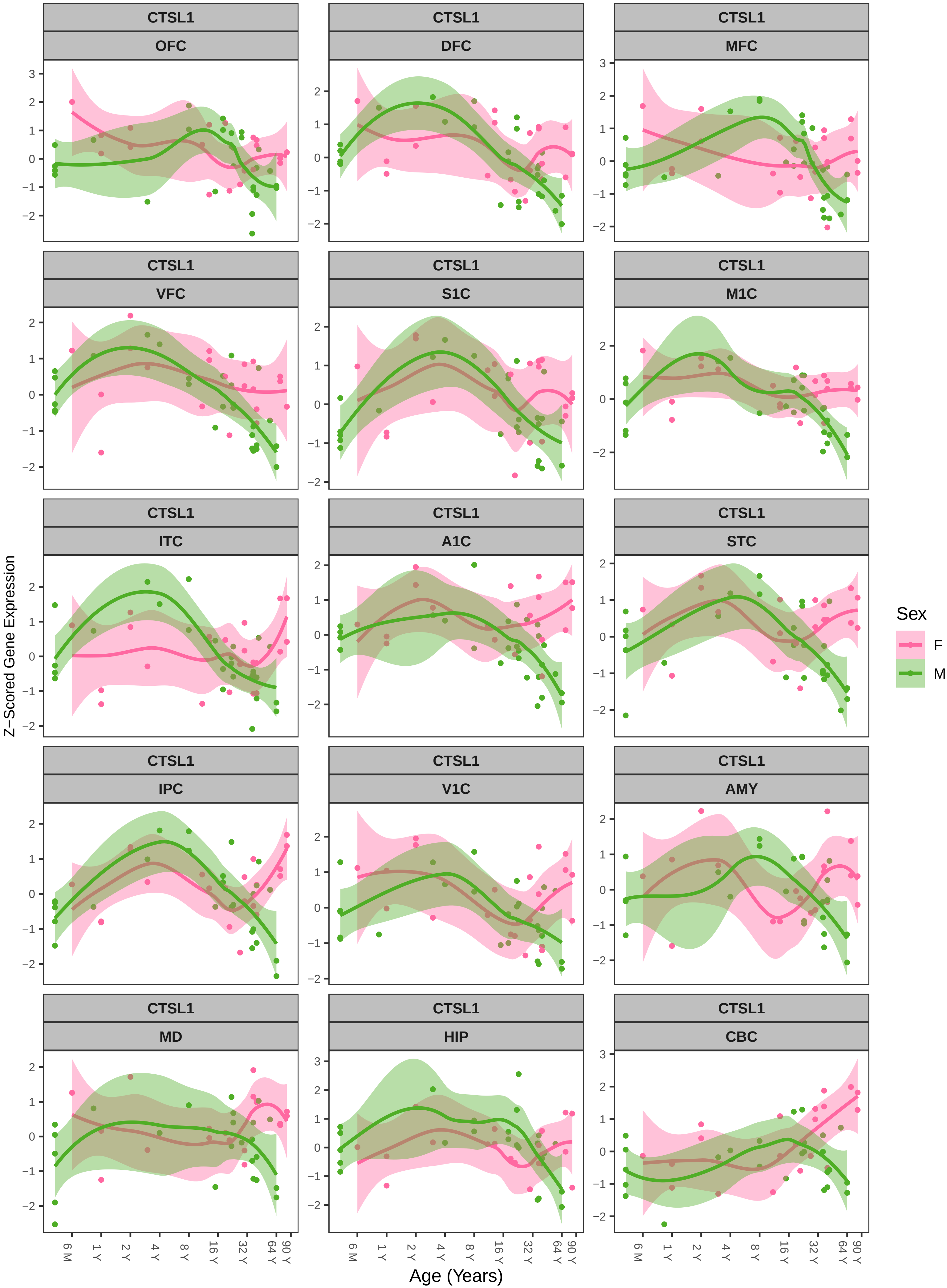

CTSL1
OFC
DFC
MFC
VFC
S1C
M1C
ITC
A1C
STC
IPC
V1C
AMY
MD
HIP
CBC
Z−Scored Gene Expression
Age (Years)
6 M
1 Y
2 Y
4 Y
8 Y
16 Y
32 Y
64 Y
90 Y
Sex
F
M

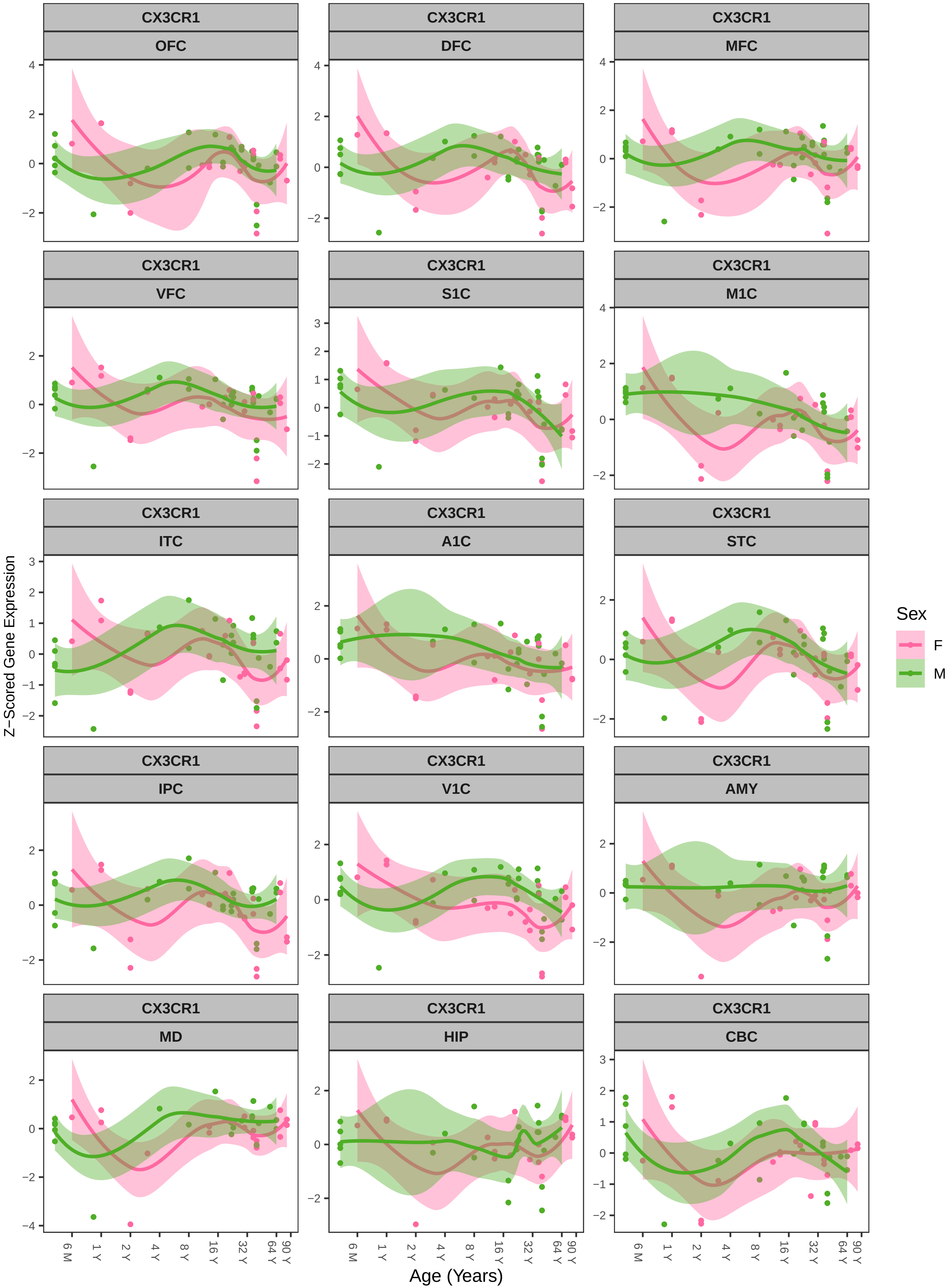

CX3CR1
OFC
DFC
MFC
VFC
S1C
M1C
ITC
A1C
STC
IPC
V1C
AMY
MD
HIP
CBC
Z−Scored Gene Expression
Age (Years)
6 M
1 Y
2 Y
4 Y
8 Y
16 Y
32 Y
64 Y
90 Y
Sex
F
M

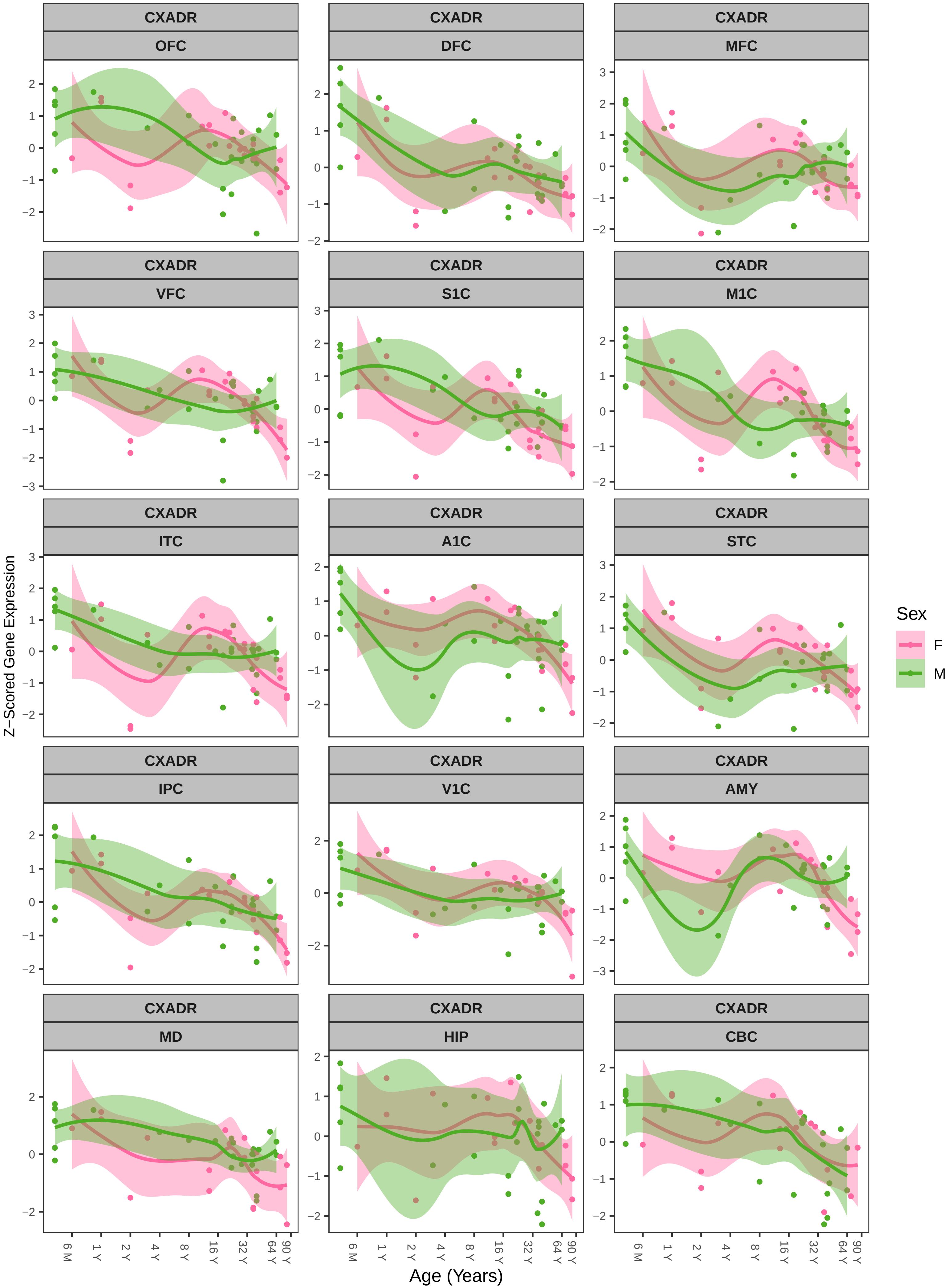
CXADR
OFC
DFC
MFC
VFC
S1C
M1C
ITC
A1C
STC
IPC
V1C
AMY
MD
HIP
CBC
Z−Scored Gene Expression
Age (Years)
6 M
1 Y
2 Y
4 Y
8 Y
16 Y
32 Y
64 Y
90 Y
Sex
F
M

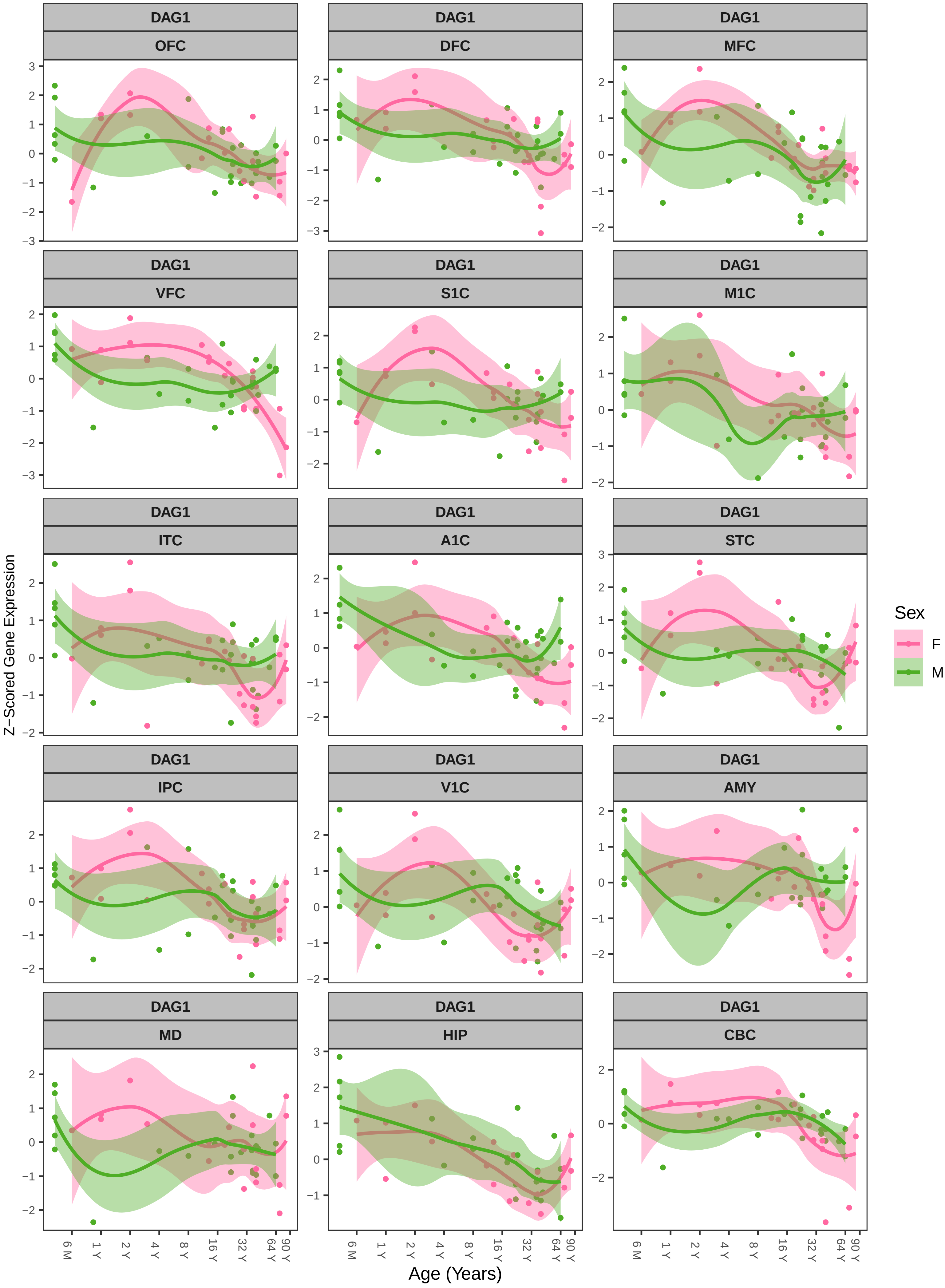
DAG1
OFC
DFC
MFC
VFC
S1C
M1C
ITC
A1C
STC
IPC
V1C
AMY
MD
HIP
CBC
Z−Scored Gene Expression
Age (Years)
6 M
1 Y
2 Y
4 Y
8 Y
16 Y
32 Y
64 Y
90 Y
Sex
F
M

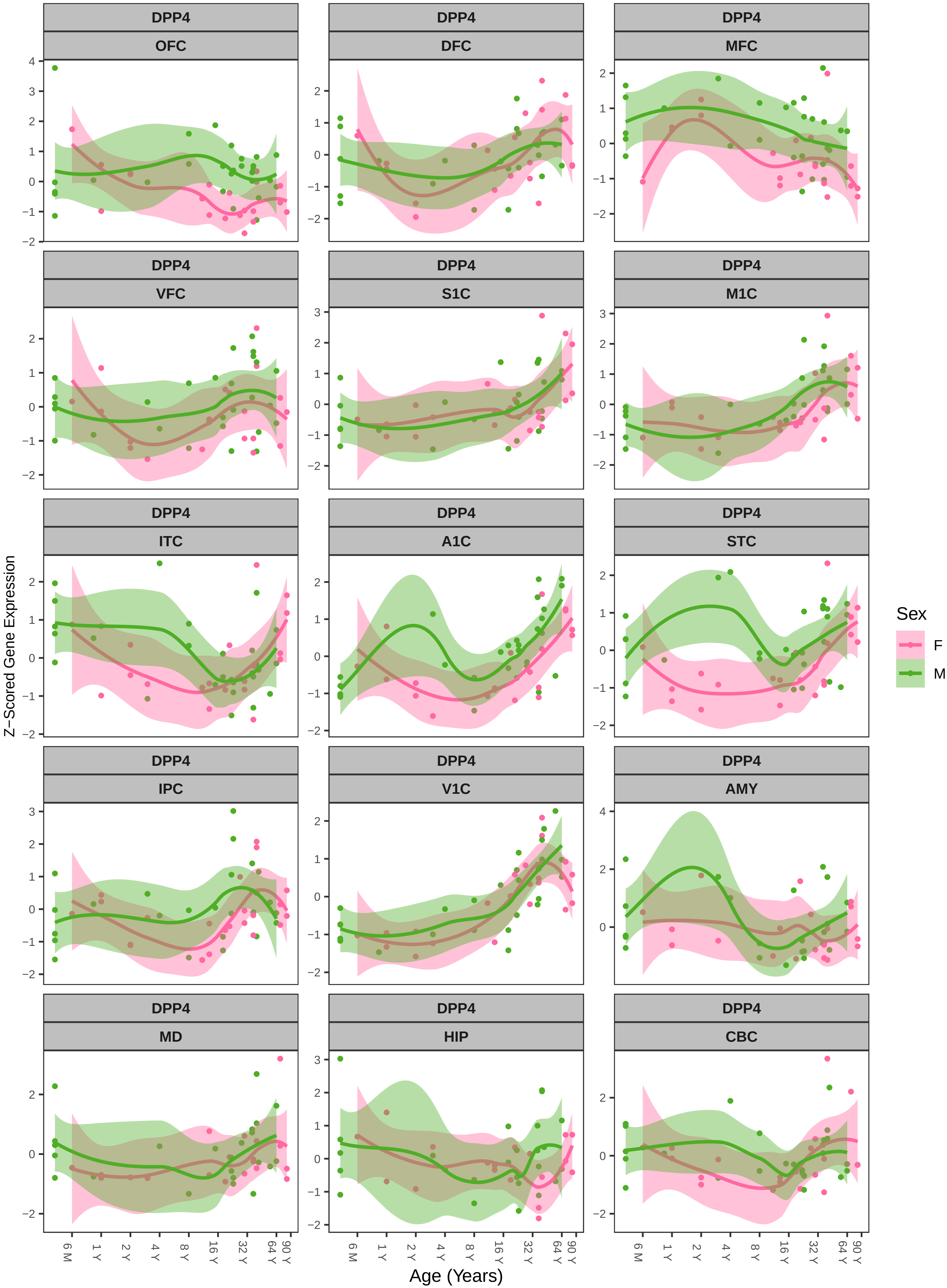

DPP4
OFC
DFC
MFC
VFC
S1C
M1C
ITC
A1C
STC
IPC
V1C
AMY
MD
HIP
CBC
Z−Scored Gene Expression
Age (Years)
6 M
1 Y
2 Y
4 Y
8 Y
16 Y
32 Y
64 Y
90 Y
Sex
F
M

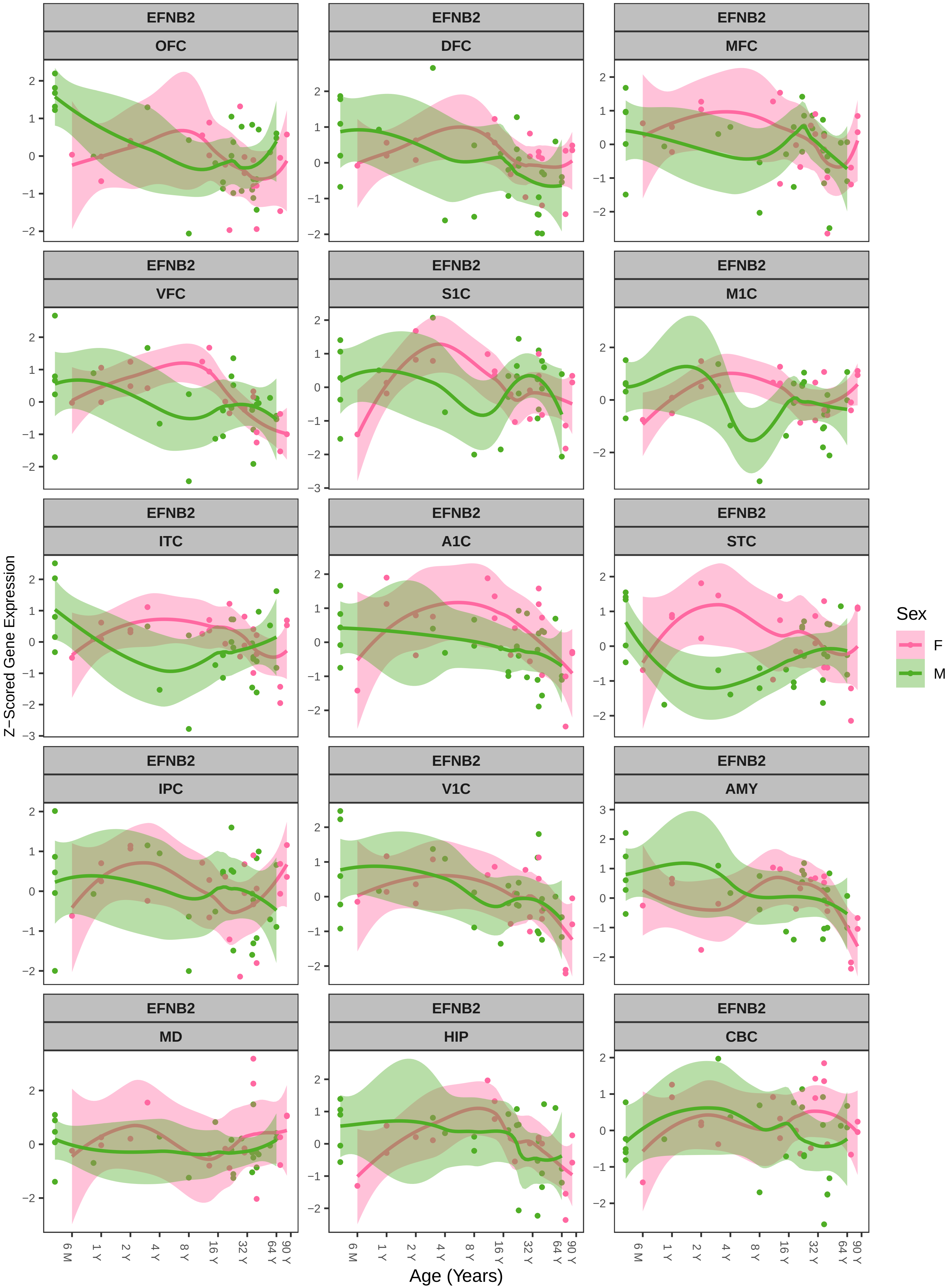

EFNB2
OFC
DFC
MFC
VFC
S1C
M1C
ITC
A1C
STC
IPC
V1C
AMY
MD
HIP
CBC
Z−Scored Gene Expression
Age (Years)
6 M
1 Y
2 Y
4 Y
8 Y
16 Y
32 Y
64 Y
90 Y
Sex
F
M

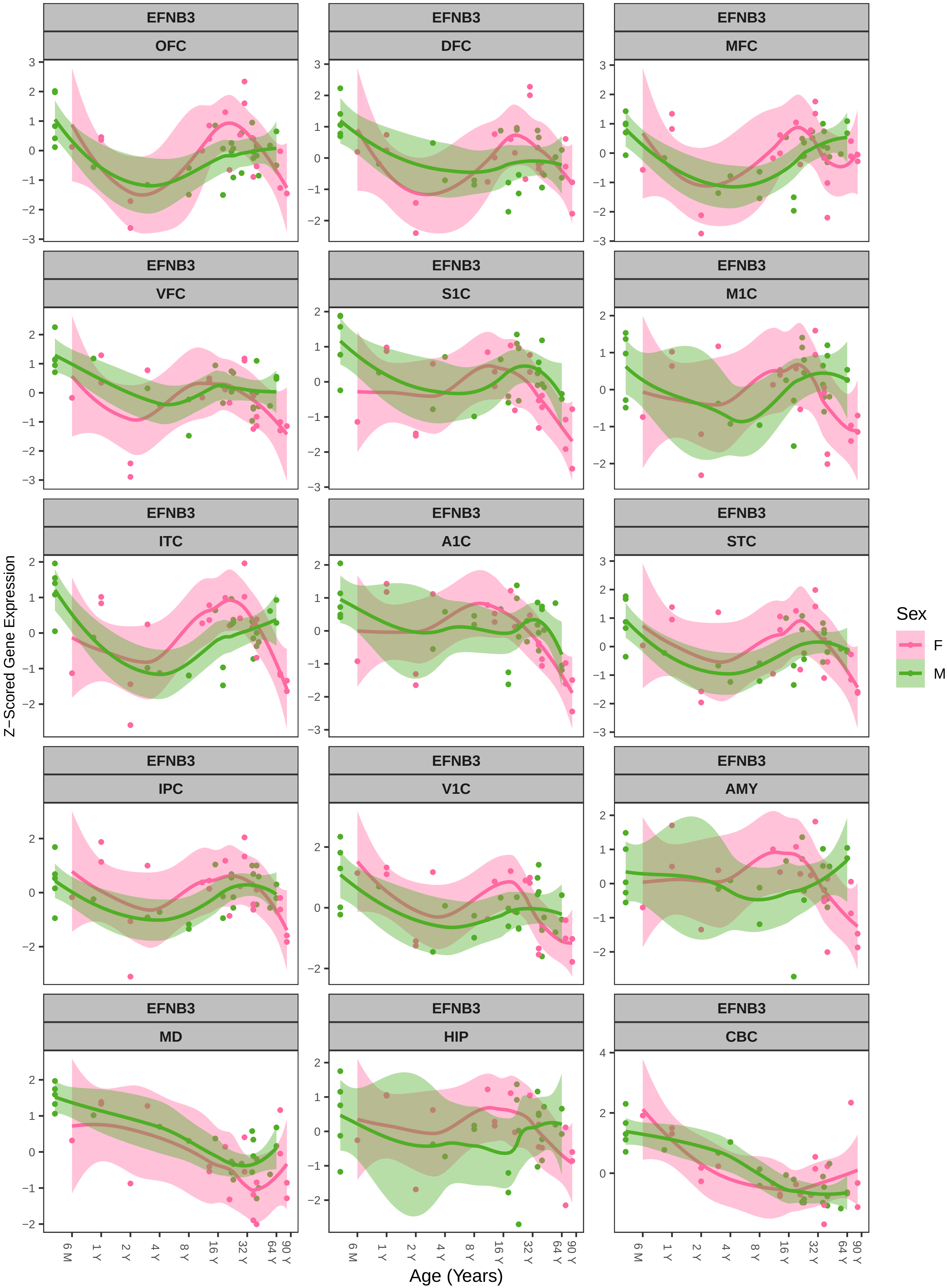
EFNB3
OFC
DFC
MFC
VFC
S1C
M1C
ITC
A1C
STC
IPC
V1C
AMY
MD
HIP
CBC
Z−Scored Gene Expression
Age (Years)
6 M
1 Y
2 Y
4 Y
8 Y
16 Y
32 Y
64 Y
90 Y
Sex
F
M

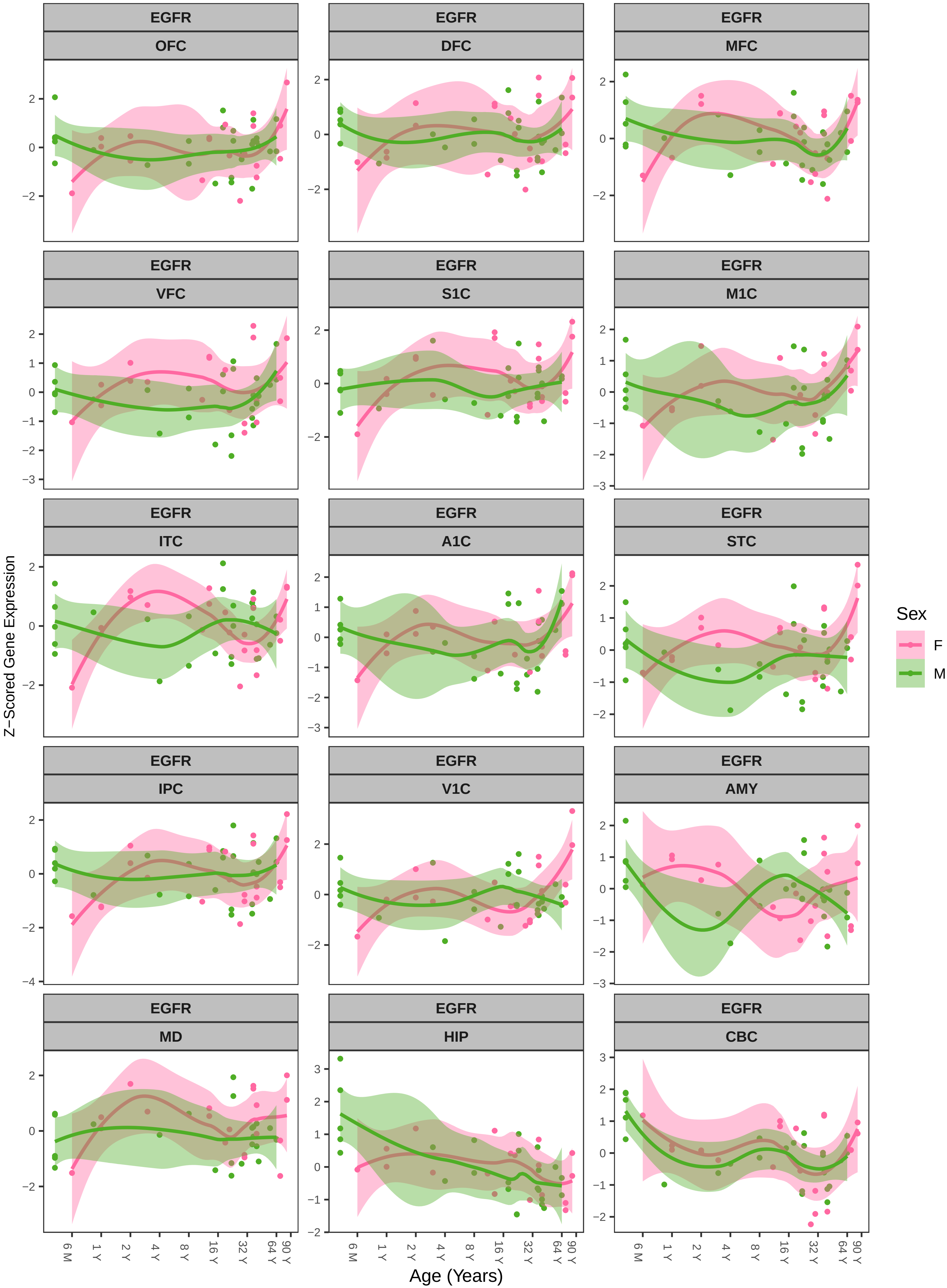

EGFR
OFC
DFC
MFC
VFC
S1C
M1C
ITC
A1C
STC
IPC
V1C
AMY
MD
HIP
CBC
Z−Scored Gene Expression
Age (Years)
6 M
1 Y
2 Y
4 Y
8 Y
16 Y
32 Y
64 Y
90 Y
Sex
F
M

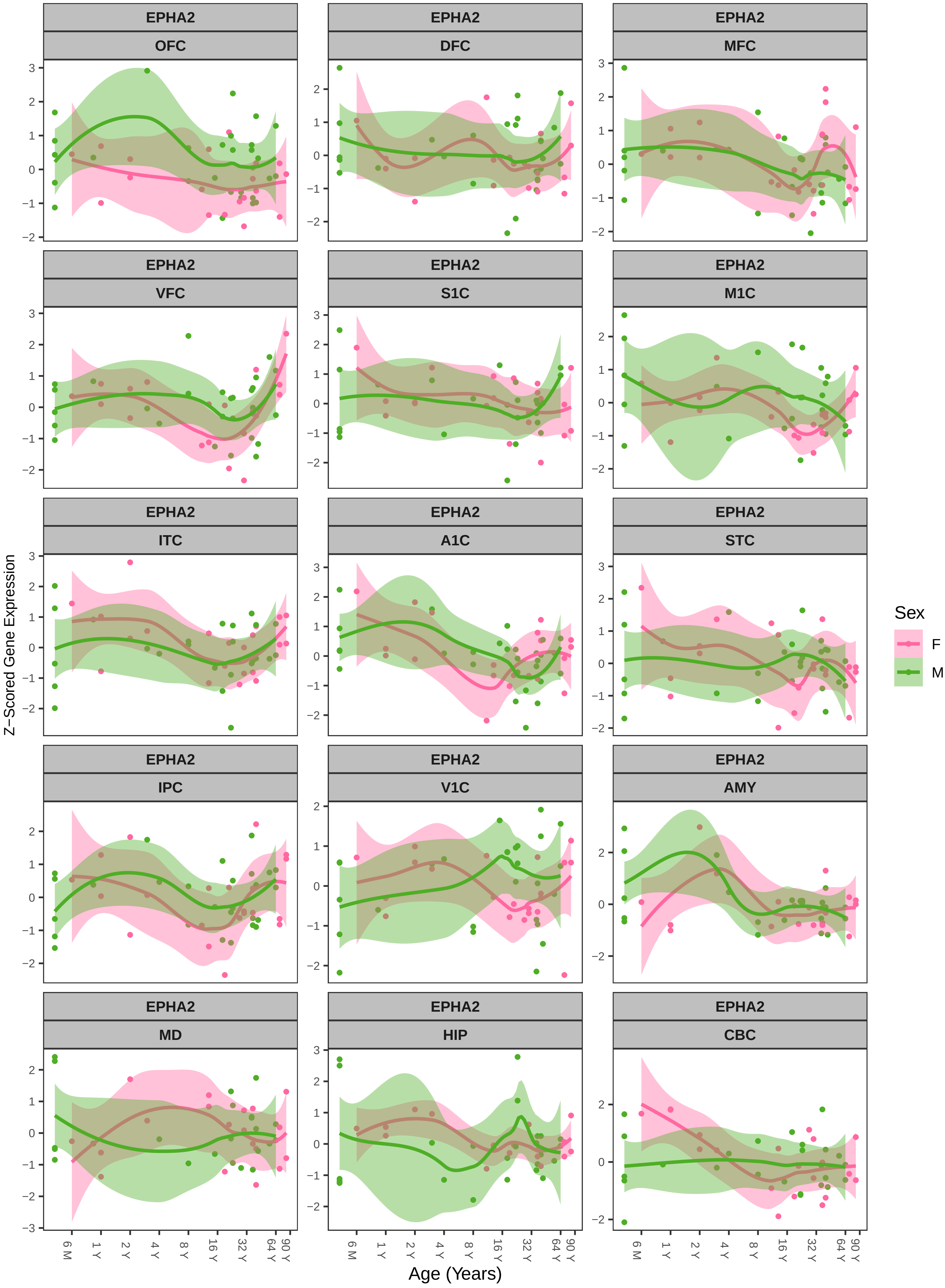

EPHA2
OFC
DFC
MFC
VFC
S1C
M1C
ITC
A1C
STC
IPC
V1C
AMY
MD
HIP
CBC
Z−Scored Gene Expression
Age (Years)
6 M
1 Y
2 Y
4 Y
8 Y
16 Y
32 Y
64 Y
90 Y
Sex
F
M

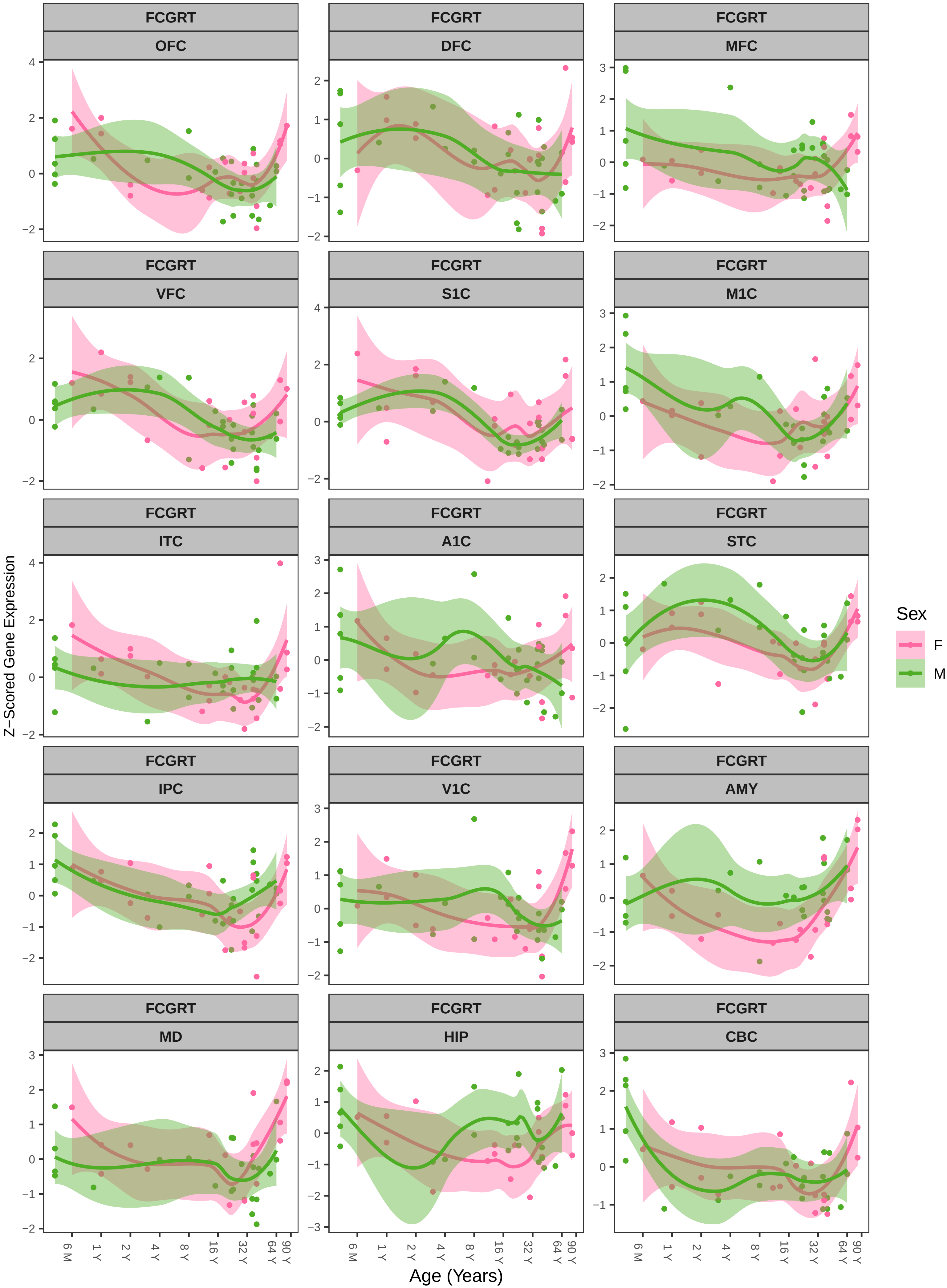

FCGRT
OFC
DFC
MFC
VFC
S1C
M1C
ITC
A1C
STC
IPC
V1C
AMY
MD
HIP
CBC
Z−Scored Gene Expression
Age (Years)
6 M
1 Y
2 Y
4 Y
8 Y
16 Y
32 Y
64 Y
90 Y
Sex
F
M

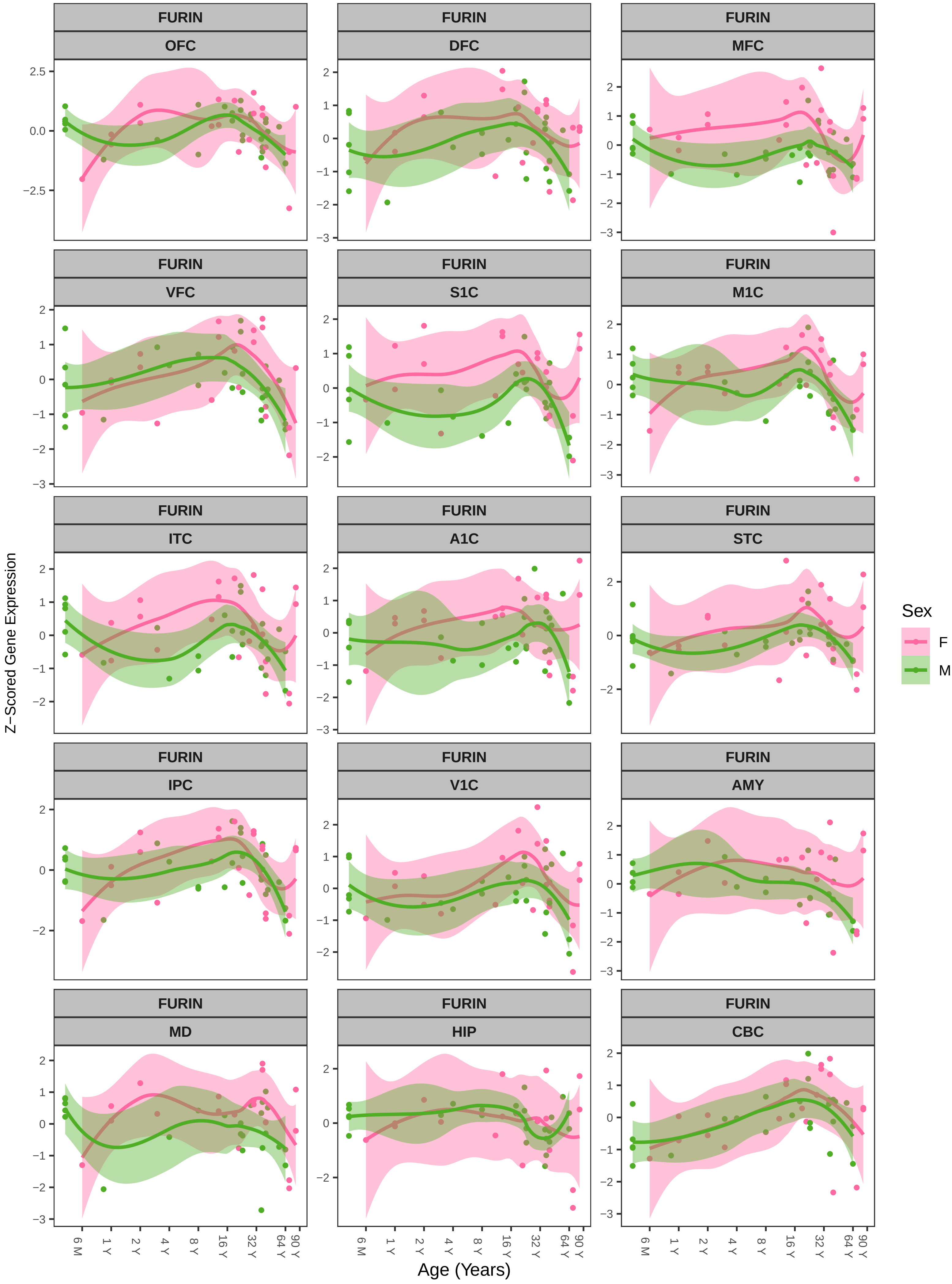

FURIN
OFC
DFC
MFC
VFC
S1C
M1C
ITC
A1C
STC
IPC
V1C
AMY
MD
HIP
CBC
Z−Scored Gene Expression
Age (Years)
6 M
1 Y
2 Y
4 Y
8 Y
16 Y
32 Y
64 Y
90 Y
Sex
F
M

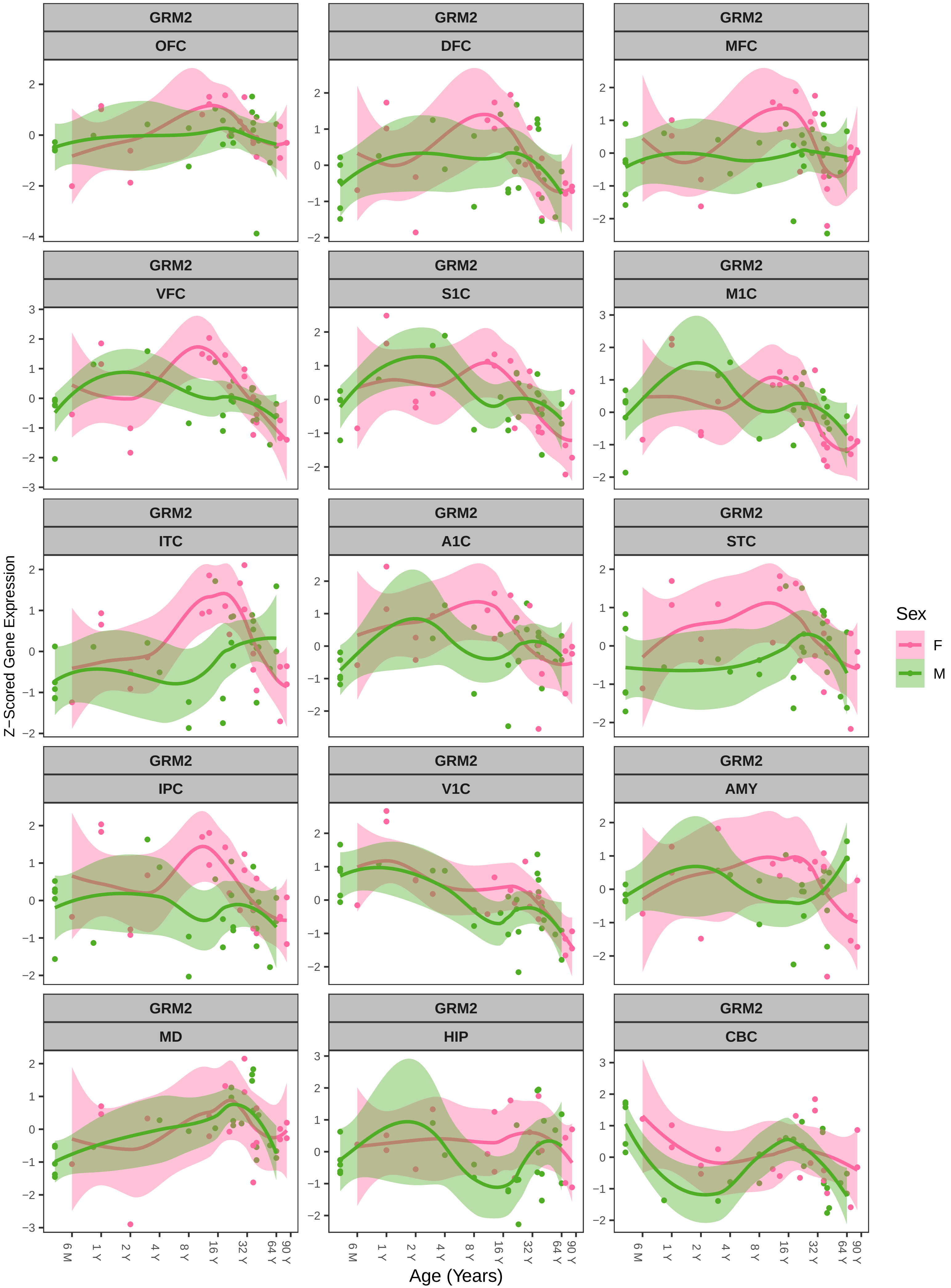

GRM2
OFC
DFC
MFC
VFC
S1C
M1C
ITC
A1C
STC
IPC
V1C
AMY
MD
HIP
CBC
Z−Scored Gene Expression
Age (Years)
6 M
1 Y
2 Y
4 Y
8 Y
16 Y
32 Y
64 Y
90 Y
Sex
F
M

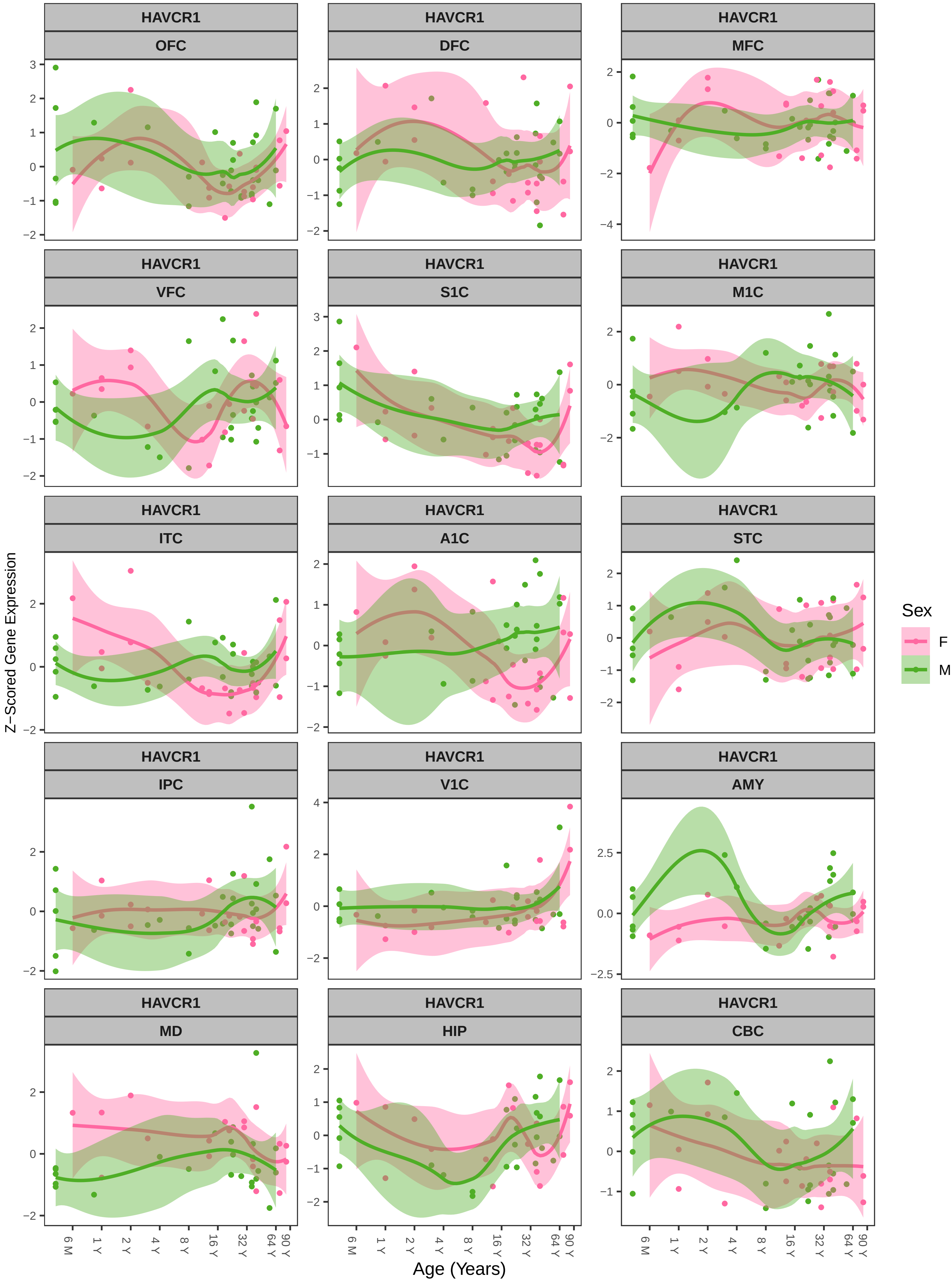

HAVCR1
OFC
DFC
MFC
VFC
S1C
M1C
ITC
A1C
STC
IPC
V1C
AMY
MD
HIP
CBC
Z−Scored Gene Expression
Age (Years)
6 M
1 Y
2 Y
4 Y
8 Y
16 Y
32 Y
64 Y
90 Y
Sex
F
M

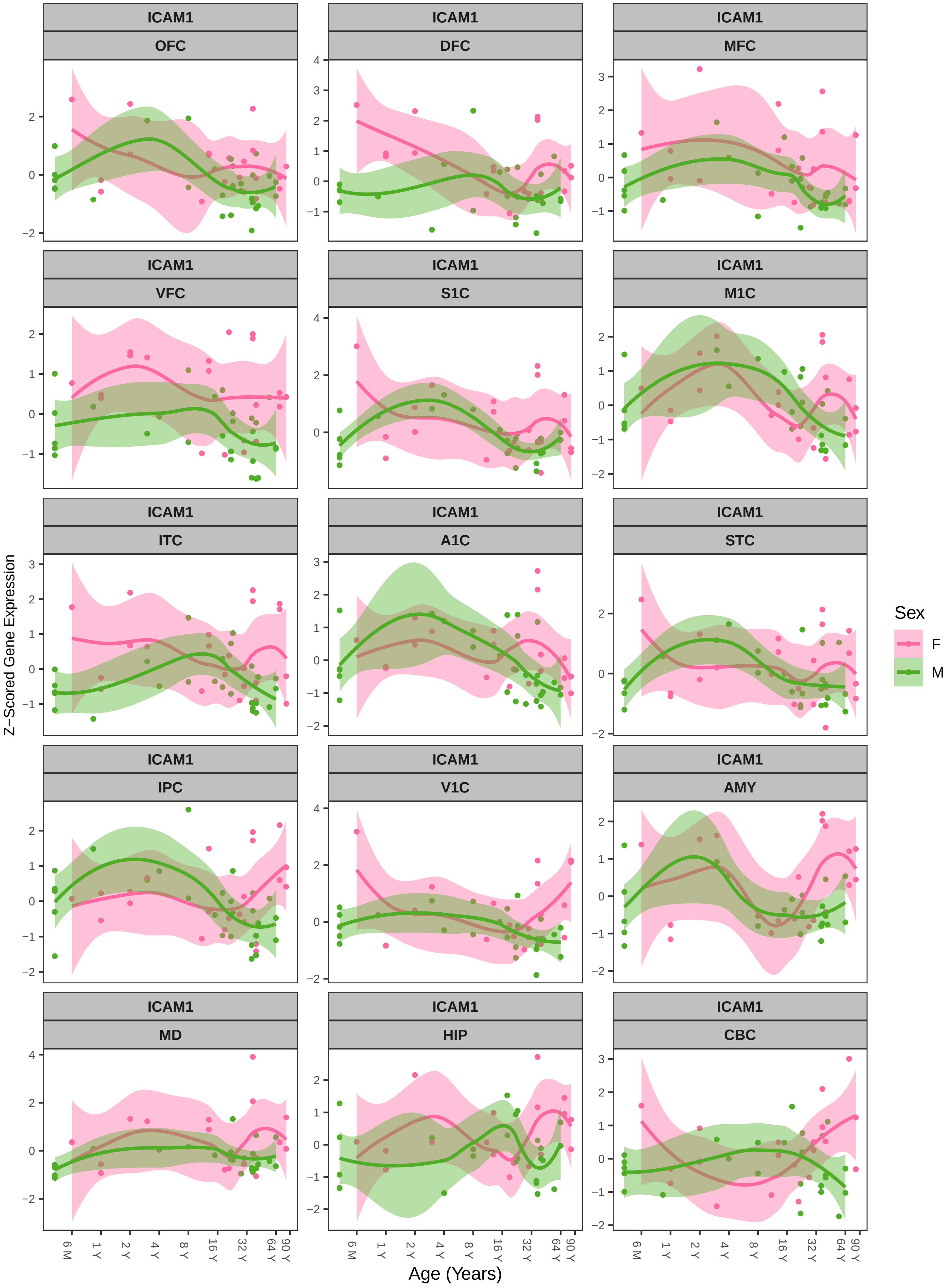
ICAM1
OFC
DFC
MFC
VFC
S1C
M1C
ITC
A1C
STC
IPC
V1C
AMY
MD
HIP
CBC
Z−Scored Gene Expression
Age (Years)
6 M
1 Y
2 Y
4 Y
8 Y
16 Y
32 Y
64 Y
90 Y
Sex
F
M

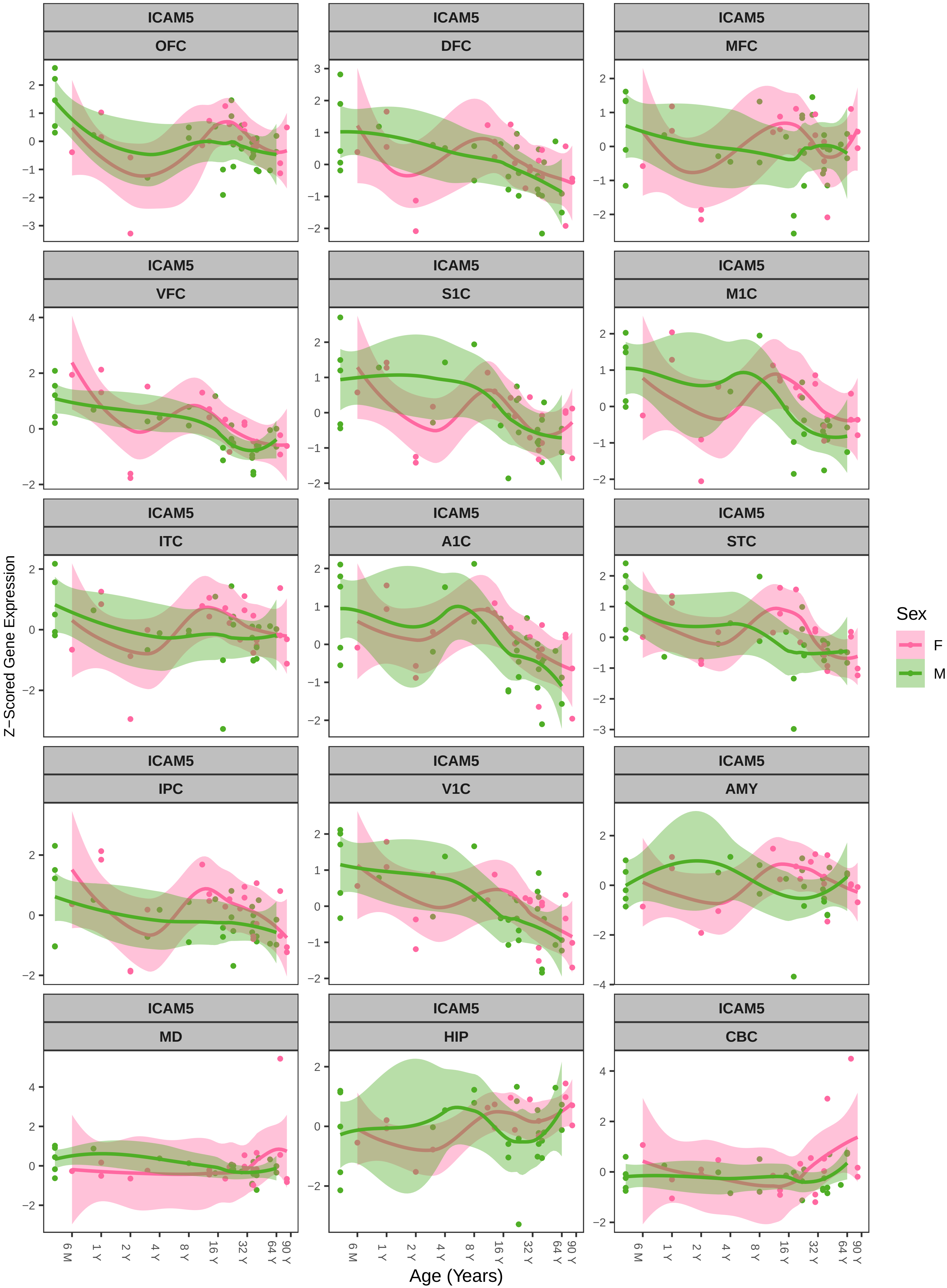
ICAM5
OFC
DFC
MFC
VFC
S1C
M1C
ITC
A1C
STC
IPC
V1C
AMY
MD
HIP
CBC
Z−Scored Gene Expression
Age (Years)
6 M
1 Y
2 Y
4 Y
8 Y
16 Y
32 Y
64 Y
90 Y
Sex
F
M

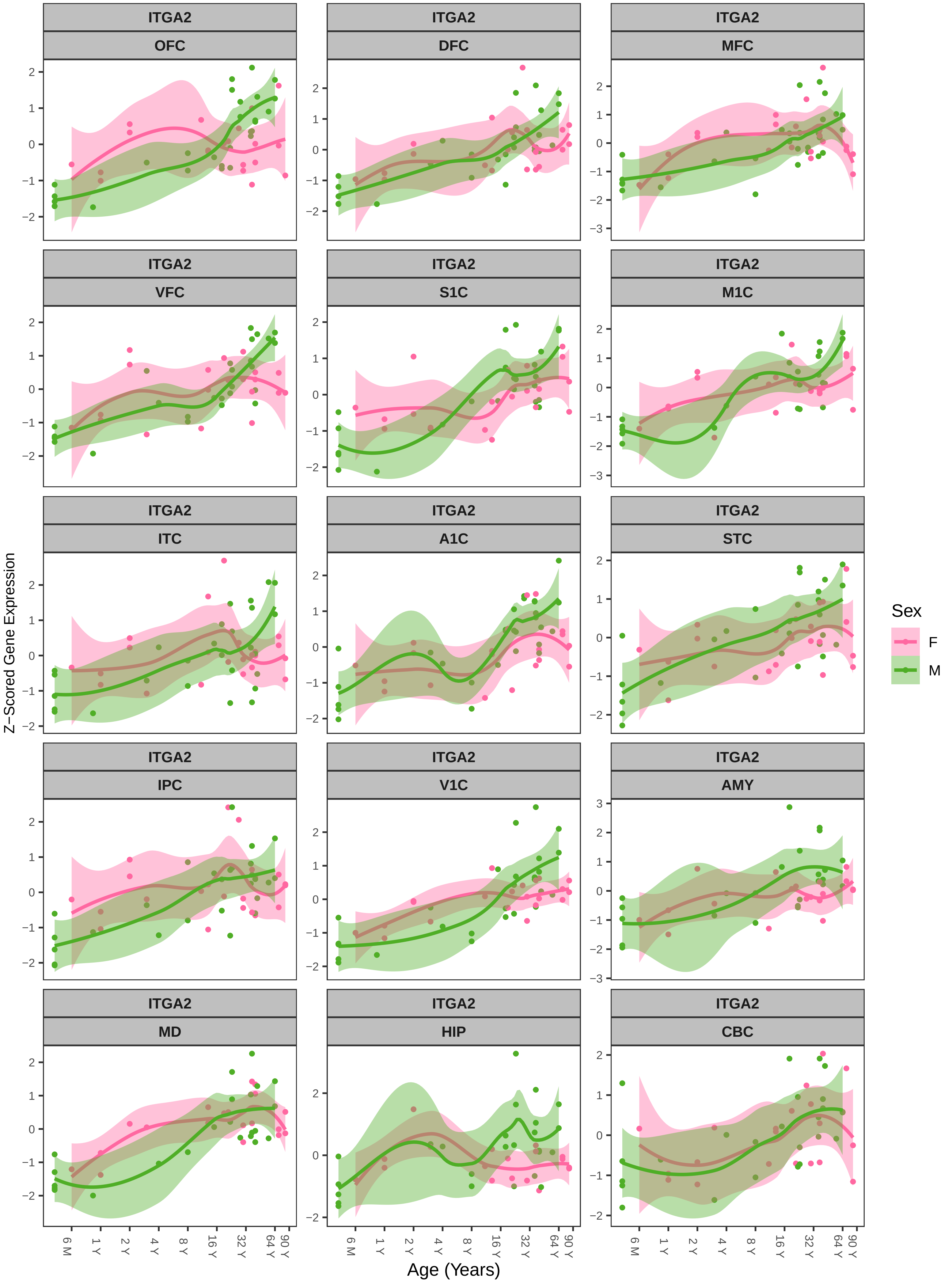
ITGA2
OFC
ITGA2
DFC
ITGA2
MFC
ITGA2
VFC
ITGA2
S1C
ITGA2
M1C
ITGA2
ITC
ITGA2
A1C
ITGA2
STC
ITGA2
IPC
ITGA2
V1C
ITGA2
AMY
ITGA2
MD
ITGA2
HIP
ITGA2
CBC
Z−Scored Gene Expression
Age (Years)
6 M
1 Y
2 Y
4 Y
8 Y
16 Y
32 Y
64 Y
90 Y
Sex
F
M

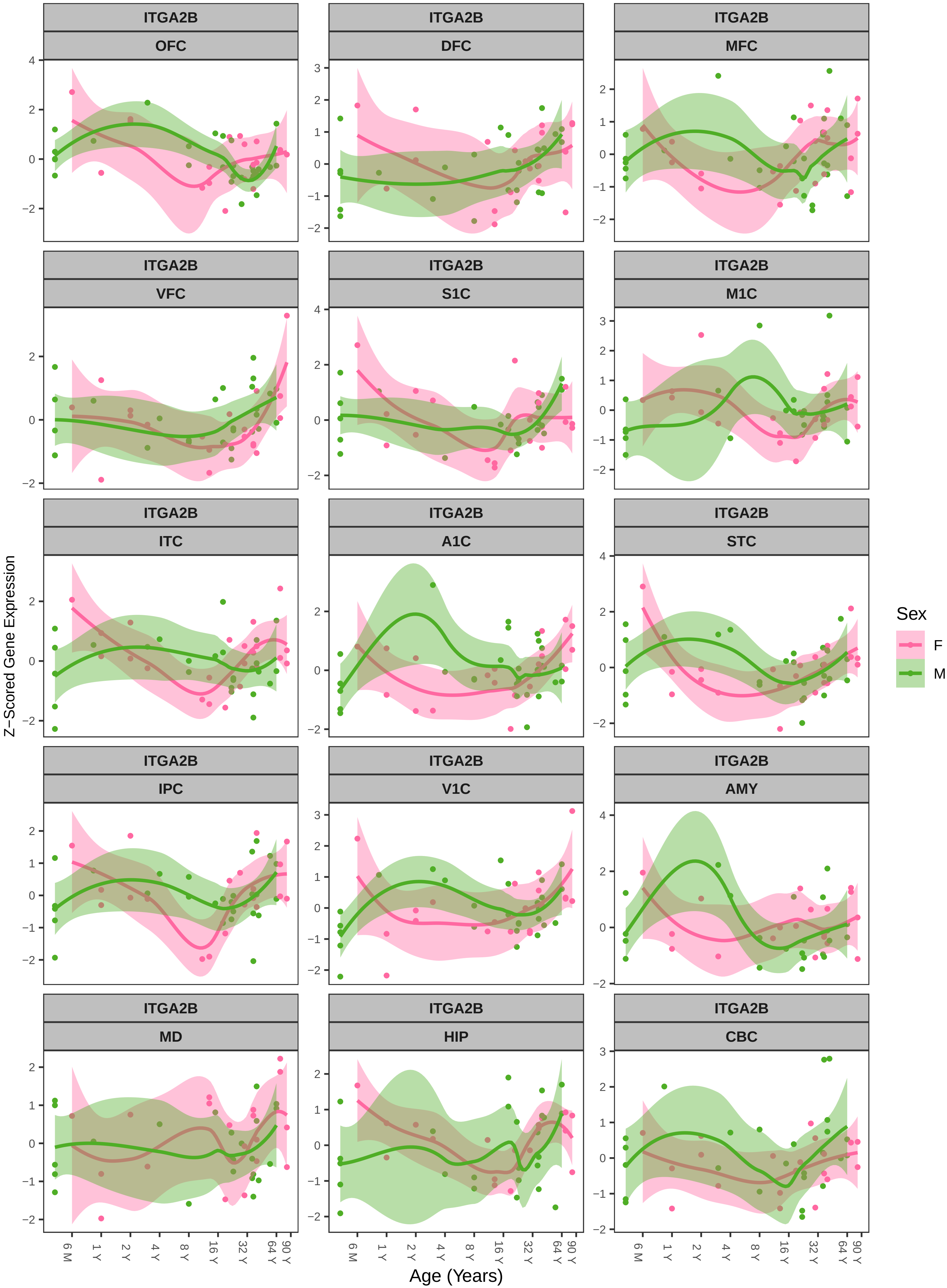
ITGA2B
OFC
DFC
MFC
VFC
S1C
M1C
ITC
A1C
STC
IPC
V1C
AMY
MD
HIP
CBC
Z−Scored Gene Expression
Age (Years)
6 M
1 Y
2 Y
4 Y
8 Y
16 Y
32 Y
64 Y
90 Y
Sex
F
M

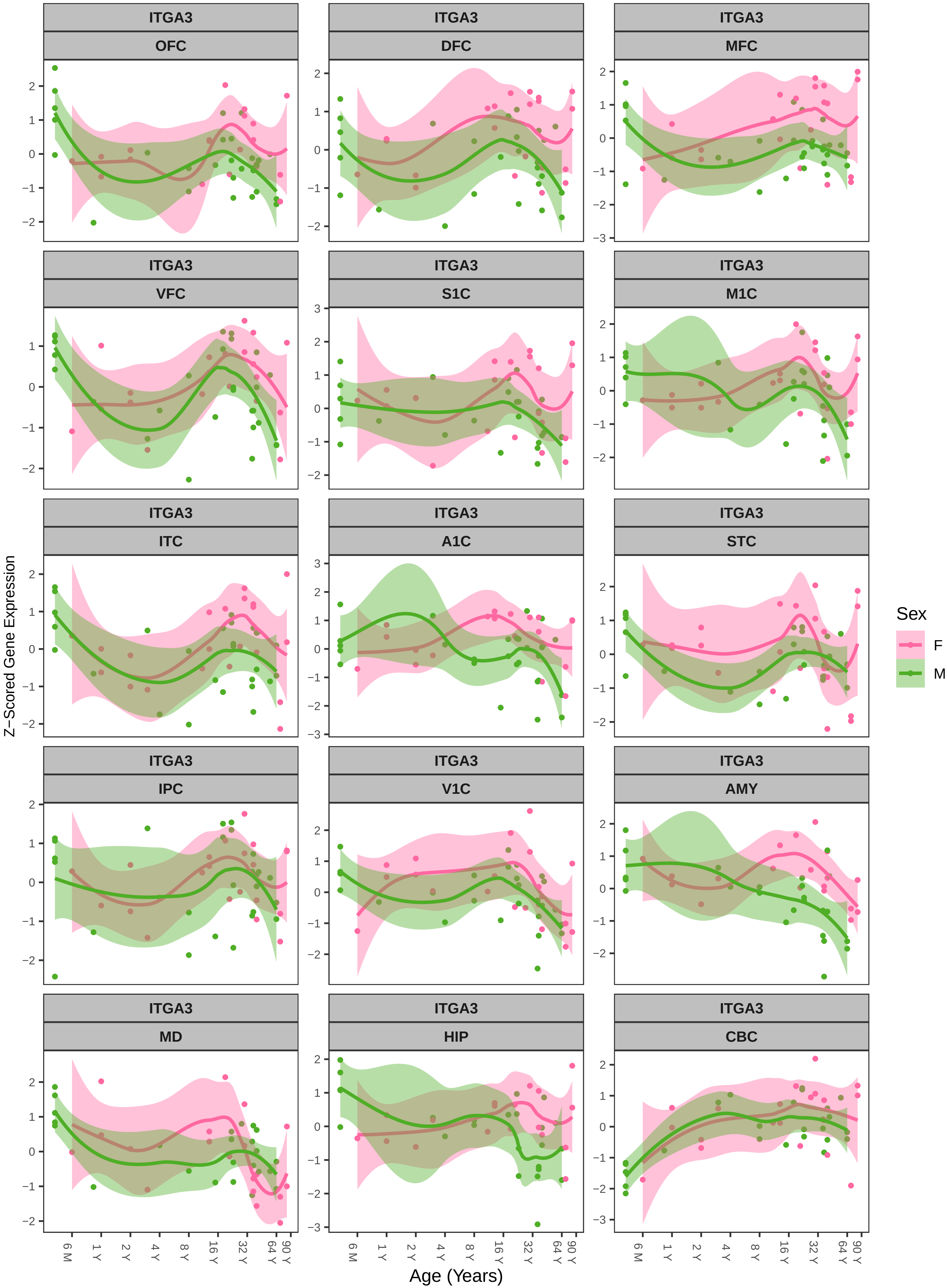

ITGA3
OFC
DFC
MFC
VFC
S1C
M1C
ITC
A1C
STC
IPC
V1C
AMY
MD
HIP
CBC
Z−Scored Gene Expression
Age (Years)
6 M
1 Y
2 Y
4 Y
8 Y
16 Y
32 Y
64 Y
90 Y
Sex
F
M

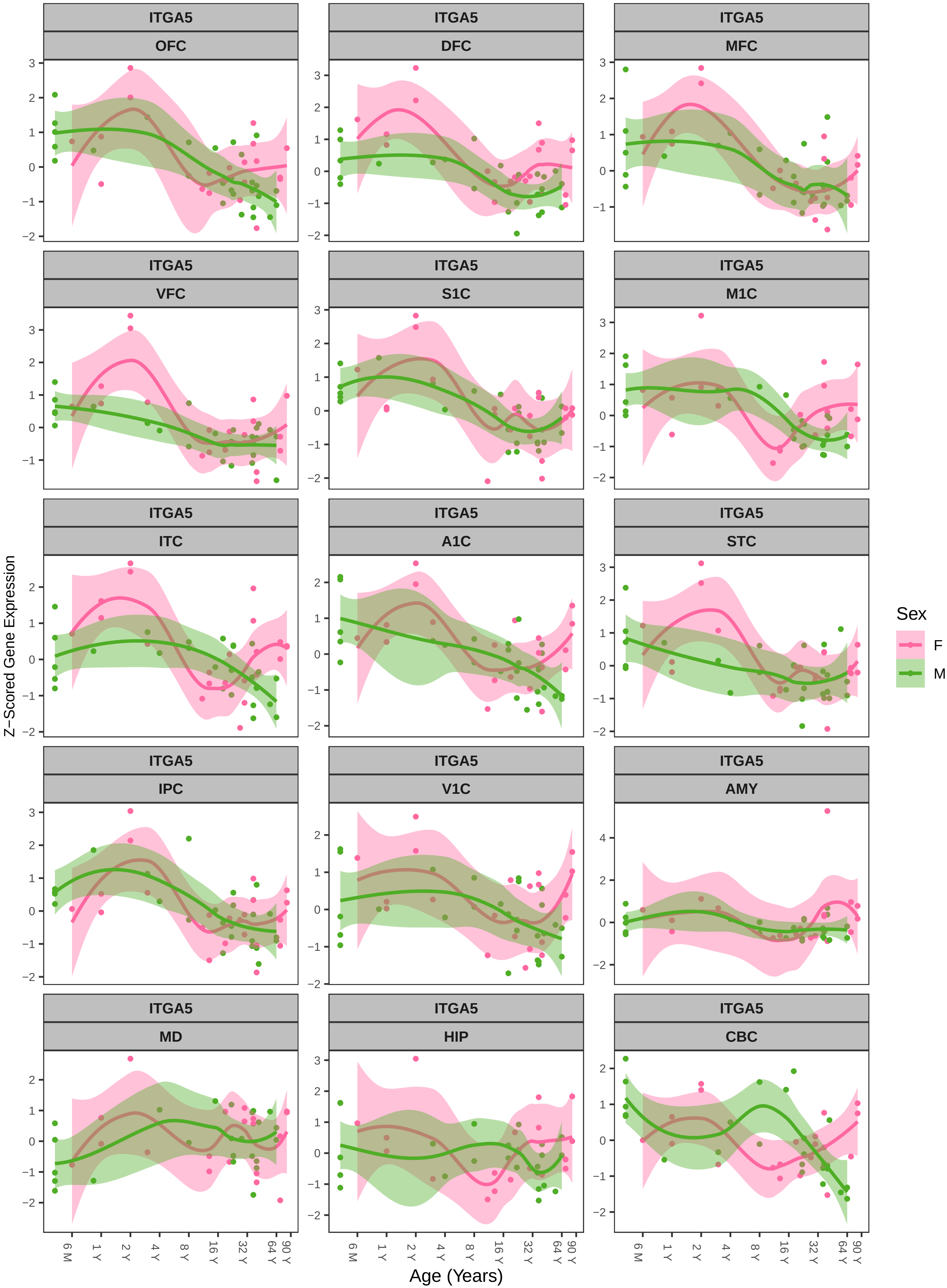
ITGA5
OFC
DFC
MFC
VFC
S1C
M1C
ITC
A1C
STC
IPC
V1C
AMY
MD
HIP
CBC
Z−Scored Gene Expression
Age (Years)
6 M
1 Y
2 Y
4 Y
8 Y
16 Y
32 Y
64 Y
90 Y
Sex
F
M

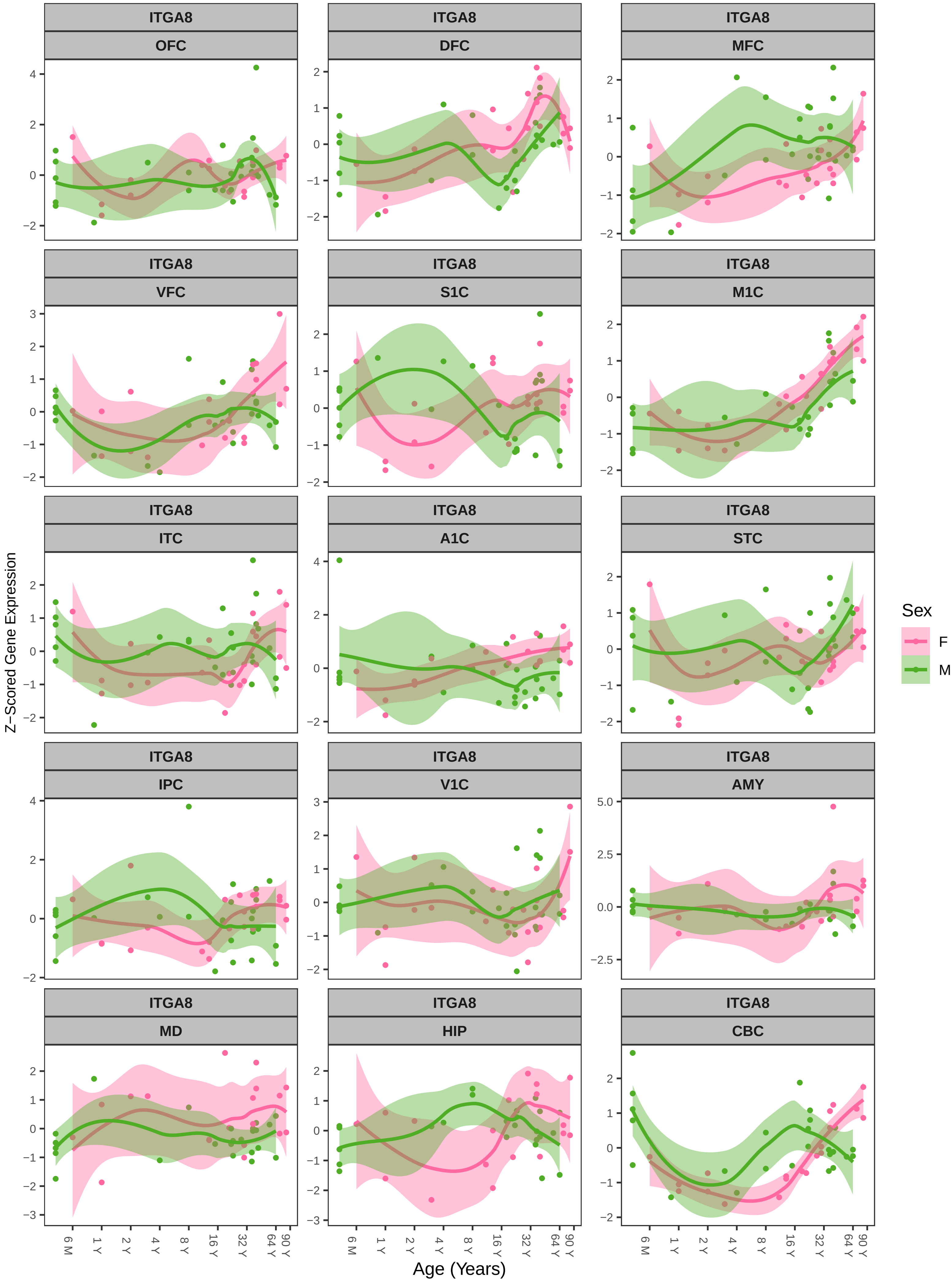
ITGA8
OFC
DFC
MFC
VFC
S1C
M1C
ITC
A1C
STC
IPC
V1C
AMY
MD
HIP
CBC
Z−Scored Gene Expression
Age (Years)
6 M
1 Y
2 Y
4 Y
8 Y
16 Y
32 Y
64 Y
90 Y
Sex
F
M

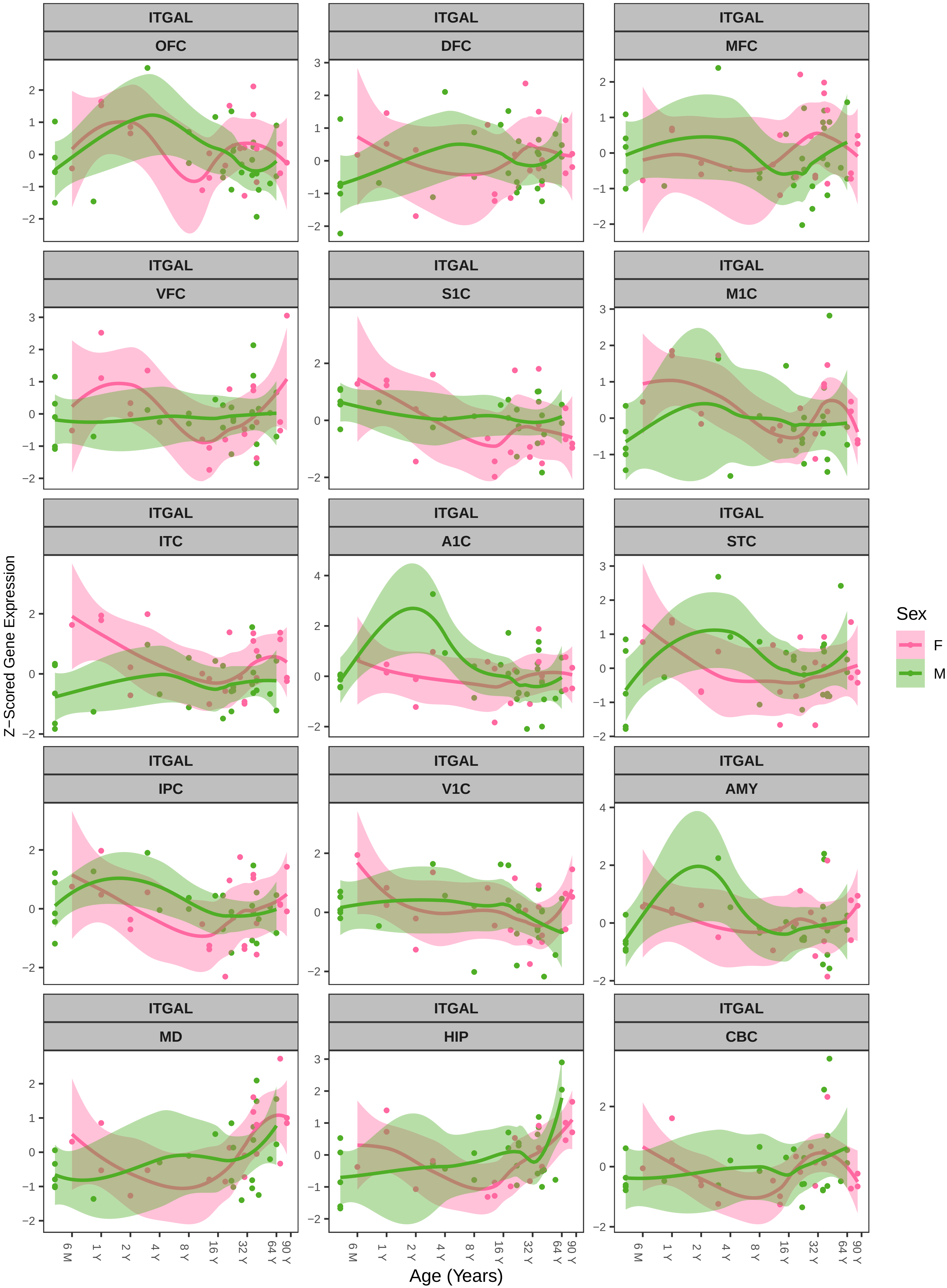

ITGAL
OFC
DFC
MFC
VFC
S1C
M1C
ITC
A1C
STC
IPC
V1C
AMY
MD
HIP
CBC
Z−Scored Gene Expression
Age (Years)
6 M
1 Y
2 Y
4 Y
8 Y
16 Y
32 Y
64 Y
90 Y
Sex
F
M

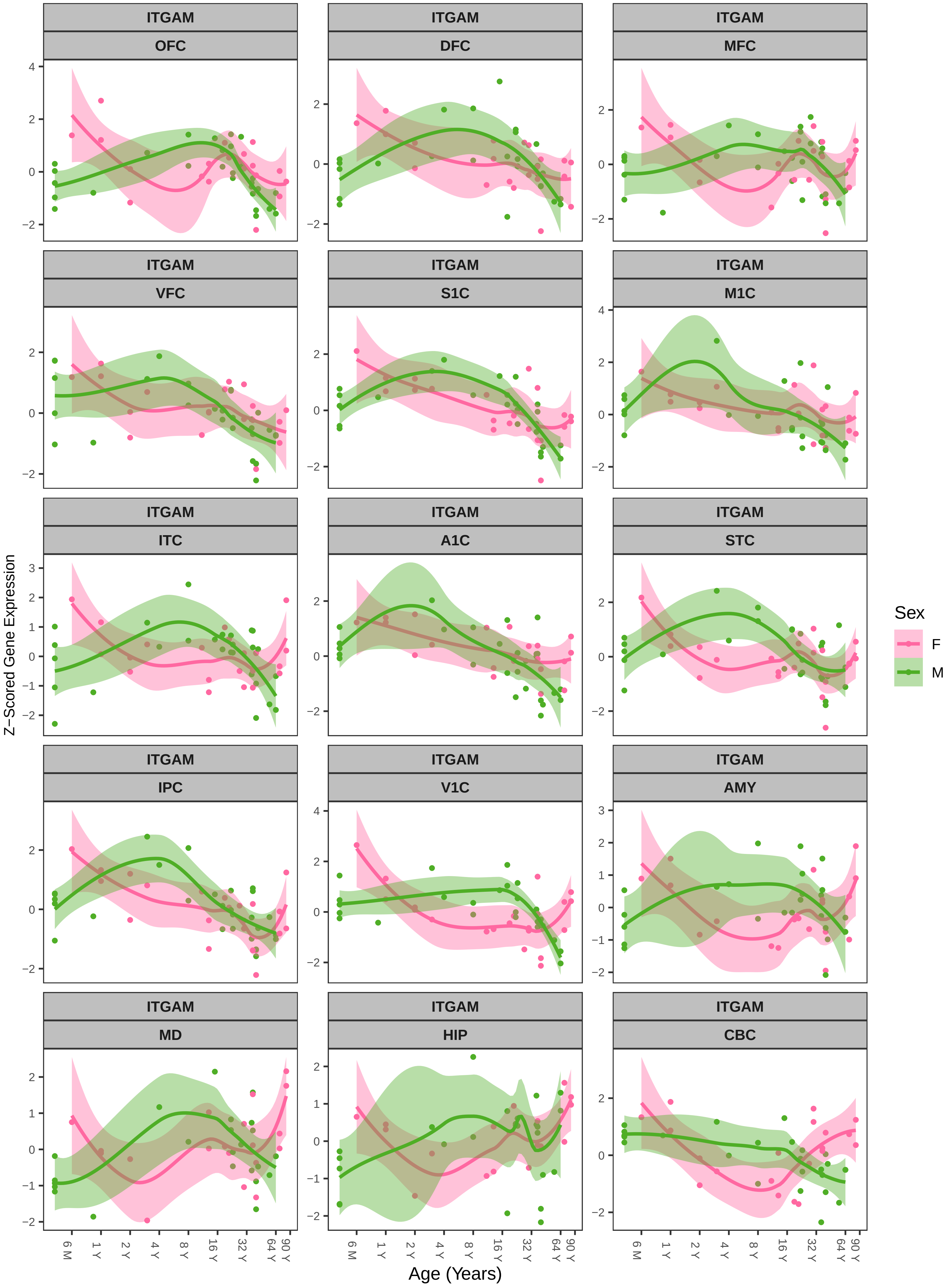
ITGAM
OFC
DFC
MFC
VFC
S1C
M1C
ITC
A1C
STC
IPC
V1C
AMY
MD
HIP
CBC
Z−Scored Gene Expression
Age (Years)
6 M
1 Y
2 Y
4 Y
8 Y
16 Y
32 Y
64 Y
90 Y
Sex
F
M

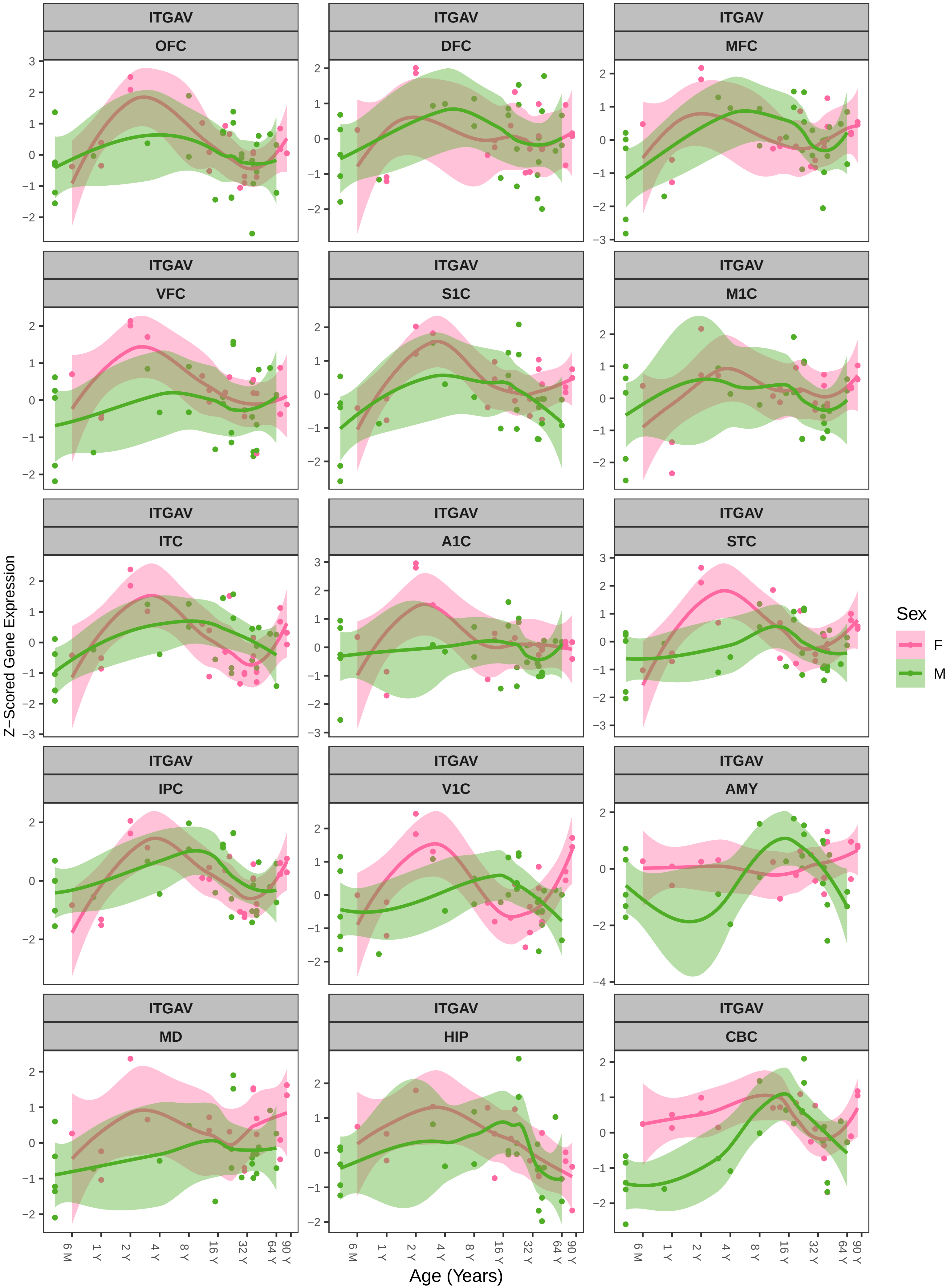
ITGAV
OFC
DFC
MFC
VFC
S1C
M1C
ITC
A1C
STC
IPC
V1C
AMY
MD
HIP
CBC
Z−Scored Gene Expression
Age (Years)
6 M
1 Y
2 Y
4 Y
8 Y
16 Y
32 Y
64 Y
90 Y
Sex
F
M

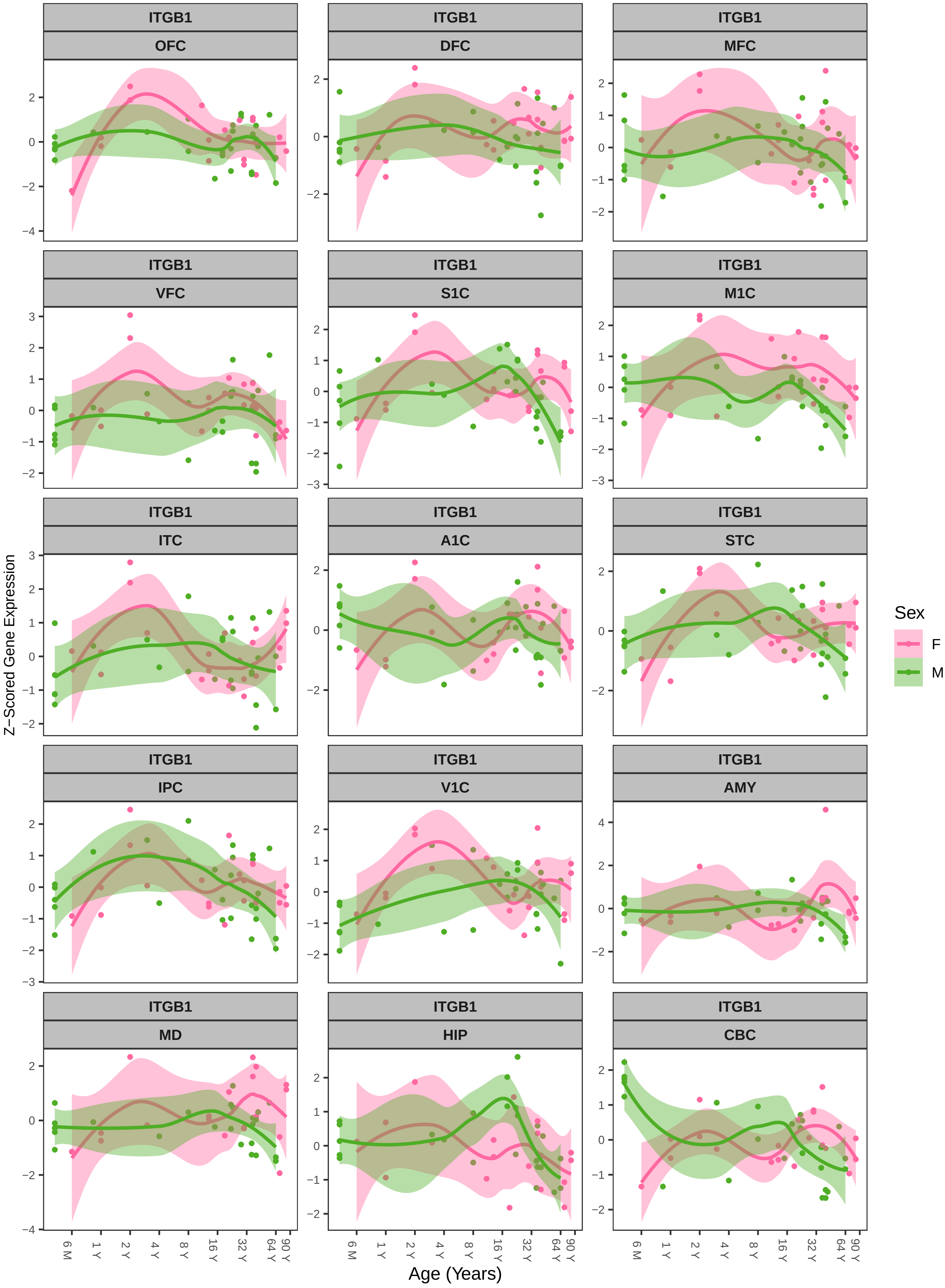
ITGB1
OFC
DFC
MFC
VFC
S1C
M1C
ITC
A1C
STC
IPC
V1C
AMY
MD
HIP
CBC
Z−Scored Gene Expression
Age (Years)
6 M
1 Y
2 Y
4 Y
8 Y
16 Y
32 Y
64 Y
90 Y
Sex
F
M

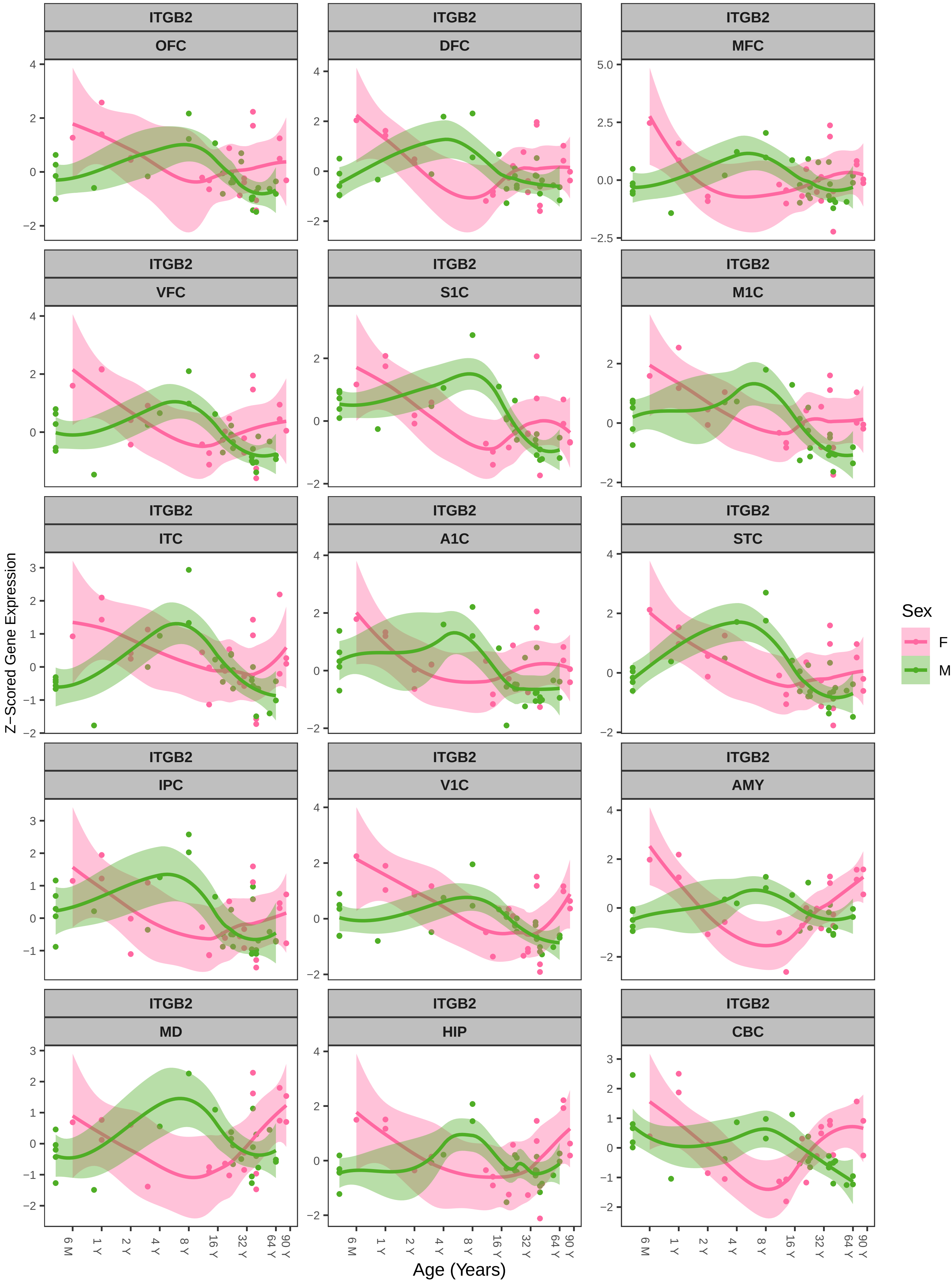
ITGB2
OFC
DFC
MFC
VFC
S1C
M1C
ITC
A1C
STC
IPC
V1C
AMY
MD
HIP
CBC
Z−Scored Gene Expression
Age (Years)
6 M
1 Y
2 Y
4 Y
8 Y
16 Y
32 Y
64 Y
90 Y
Sex
F
M

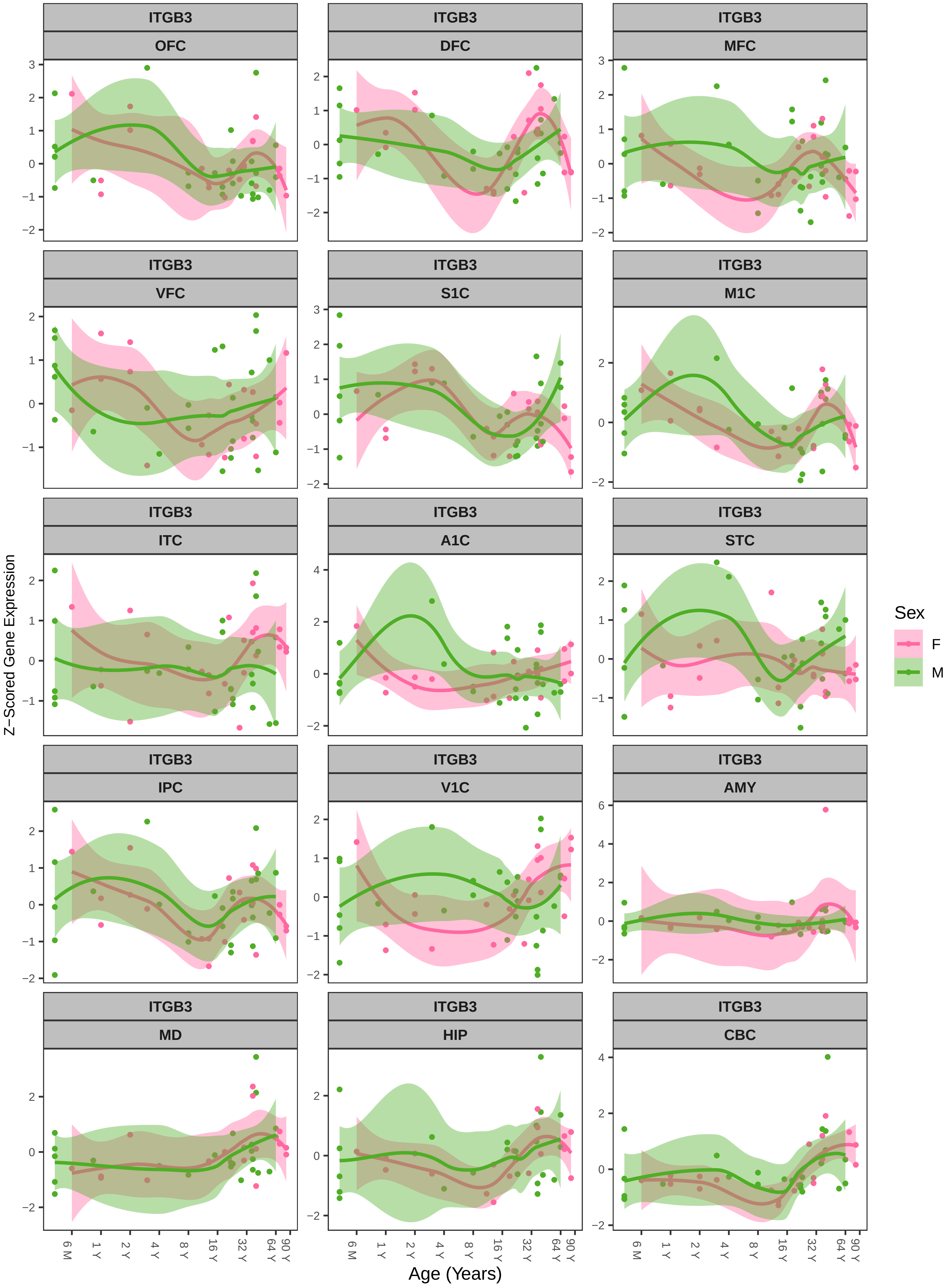

ITGB3
OFC
DFC
MFC
VFC
S1C
M1C
ITC
A1C
STC
IPC
V1C
AMY
MD
HIP
CBC
Z−Scored Gene Expression
Age (Years)
6 M
1 Y
2 Y
4 Y
8 Y
16 Y
32 Y
64 Y
90 Y
Sex
F
M

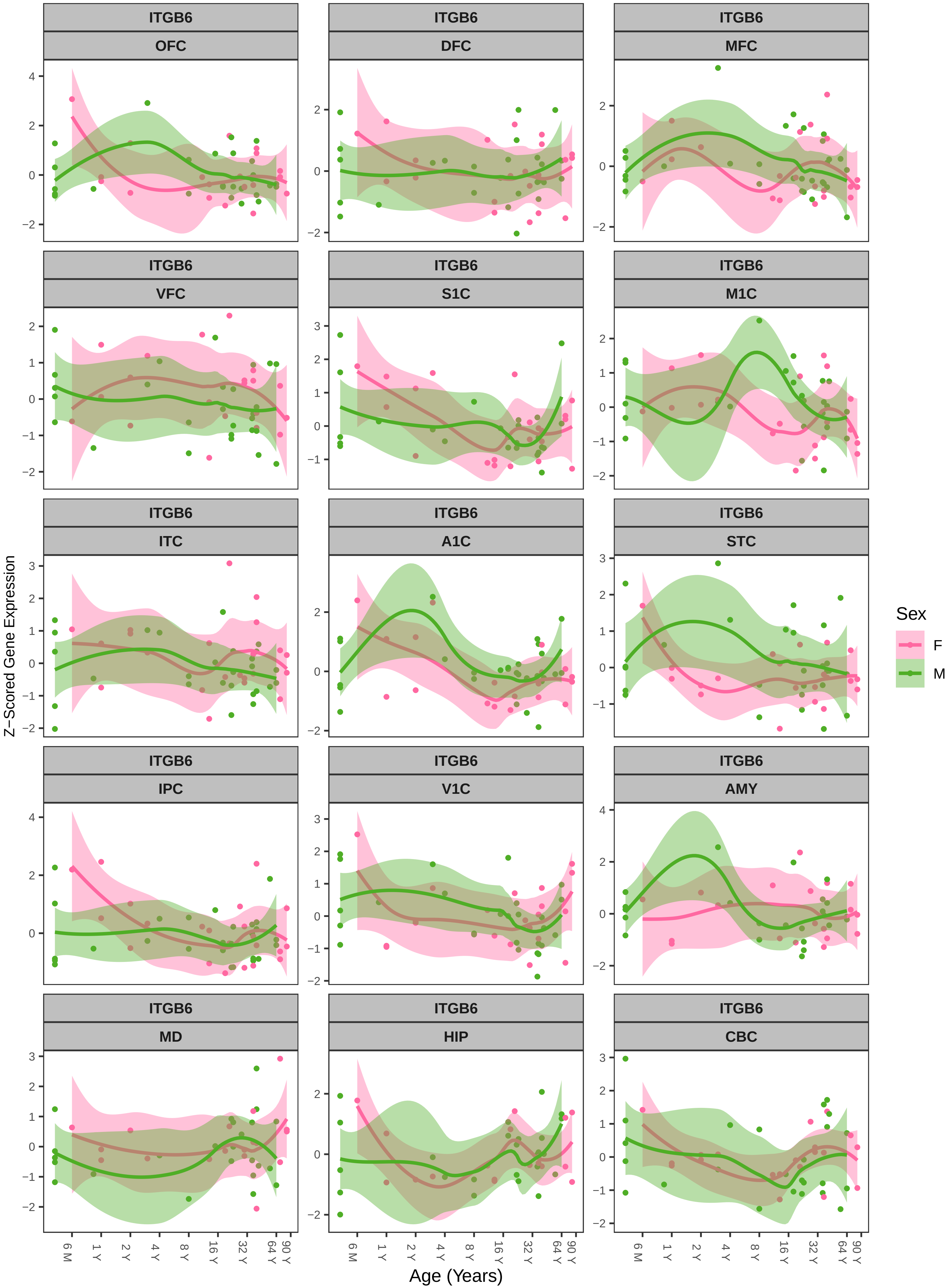
ITGB6
OFC
DFC
MFC
VFC
S1C
M1C
ITC
A1C
STC
IPC
V1C
AMY
MD
HIP
CBC
Z−Scored Gene Expression
Age (Years)
6 M
1 Y
2 Y
4 Y
8 Y
16 Y
32 Y
64 Y
90 Y
Sex
F
M

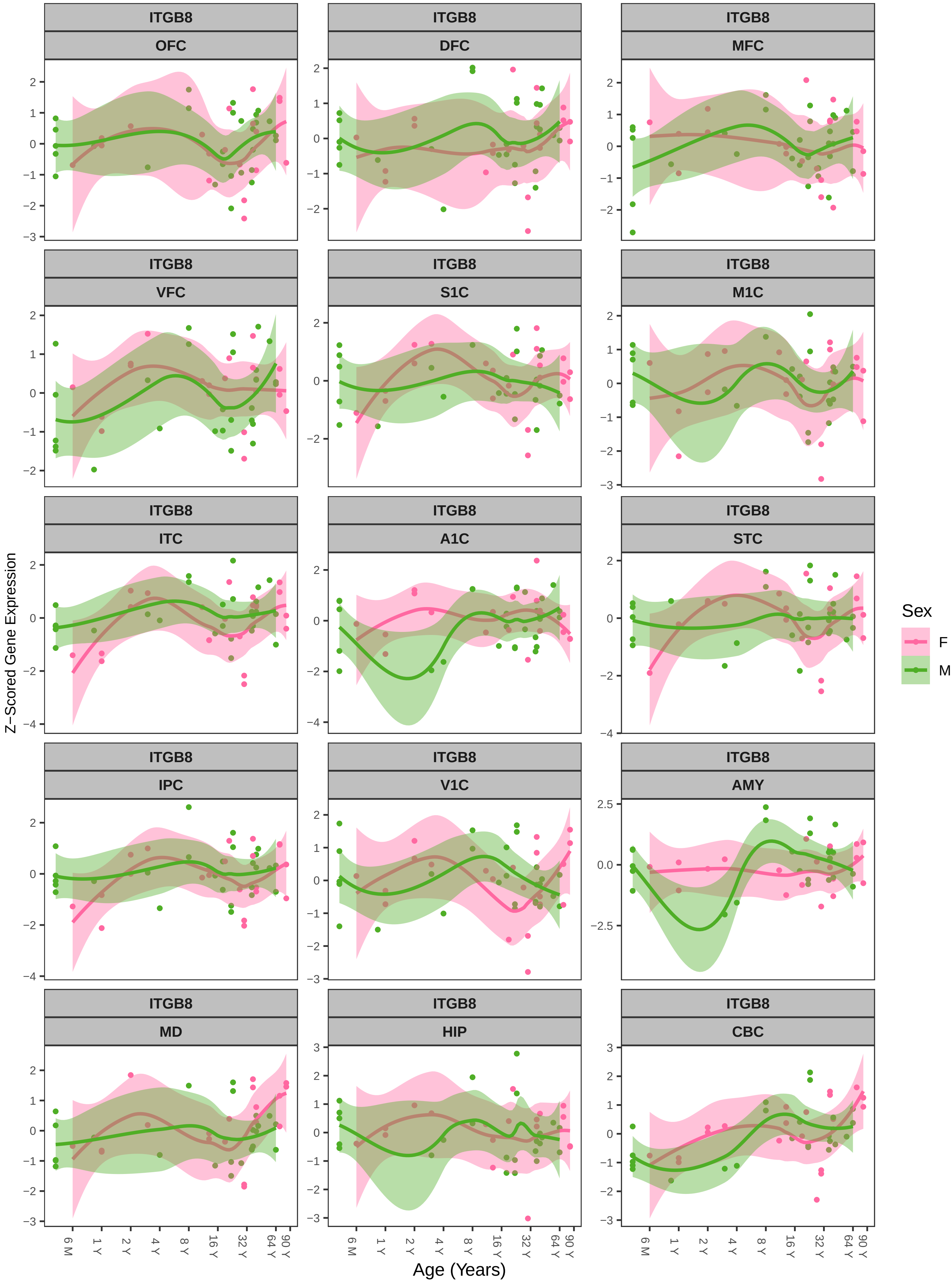
ITGB8
OFC
DFC
MFC
VFC
S1C
M1C
ITC
A1C
STC
IPC
V1C
AMY
MD
HIP
CBC
Z−Scored Gene Expression
Age (Years)
6 M
1 Y
2 Y
4 Y
8 Y
16 Y
32 Y
64 Y
90 Y
Sex
F
M

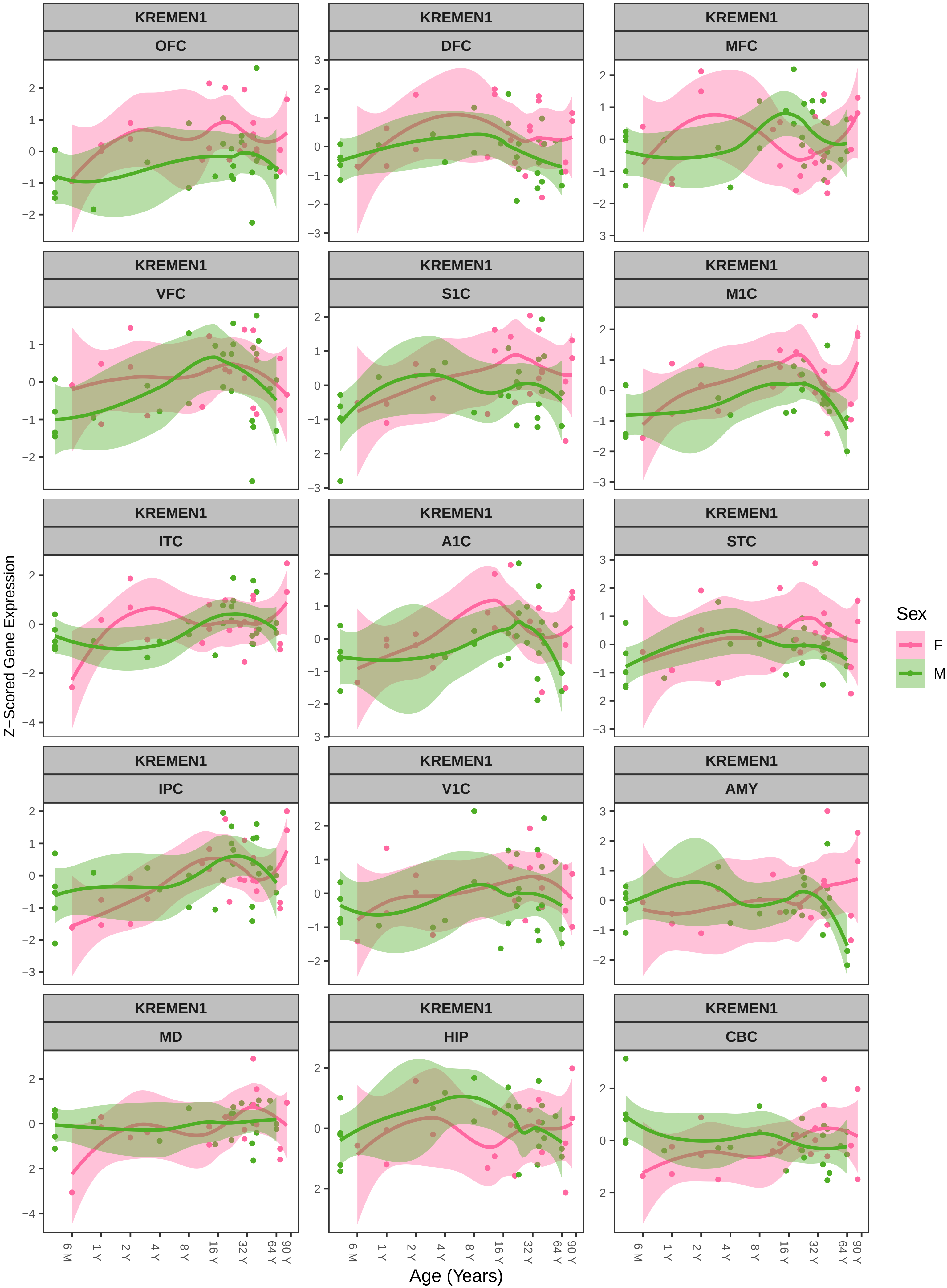
KREMEN1
OFC
DFC
MFC
VFC
S1C
M1C
ITC
A1C
STC
IPC
V1C
AMY
MD
HIP
CBC
Z−Scored Gene Expression
Age (Years)
6 M
1 Y
2 Y
4 Y
8 Y
16 Y
32 Y
64 Y
90 Y
Sex
F
M

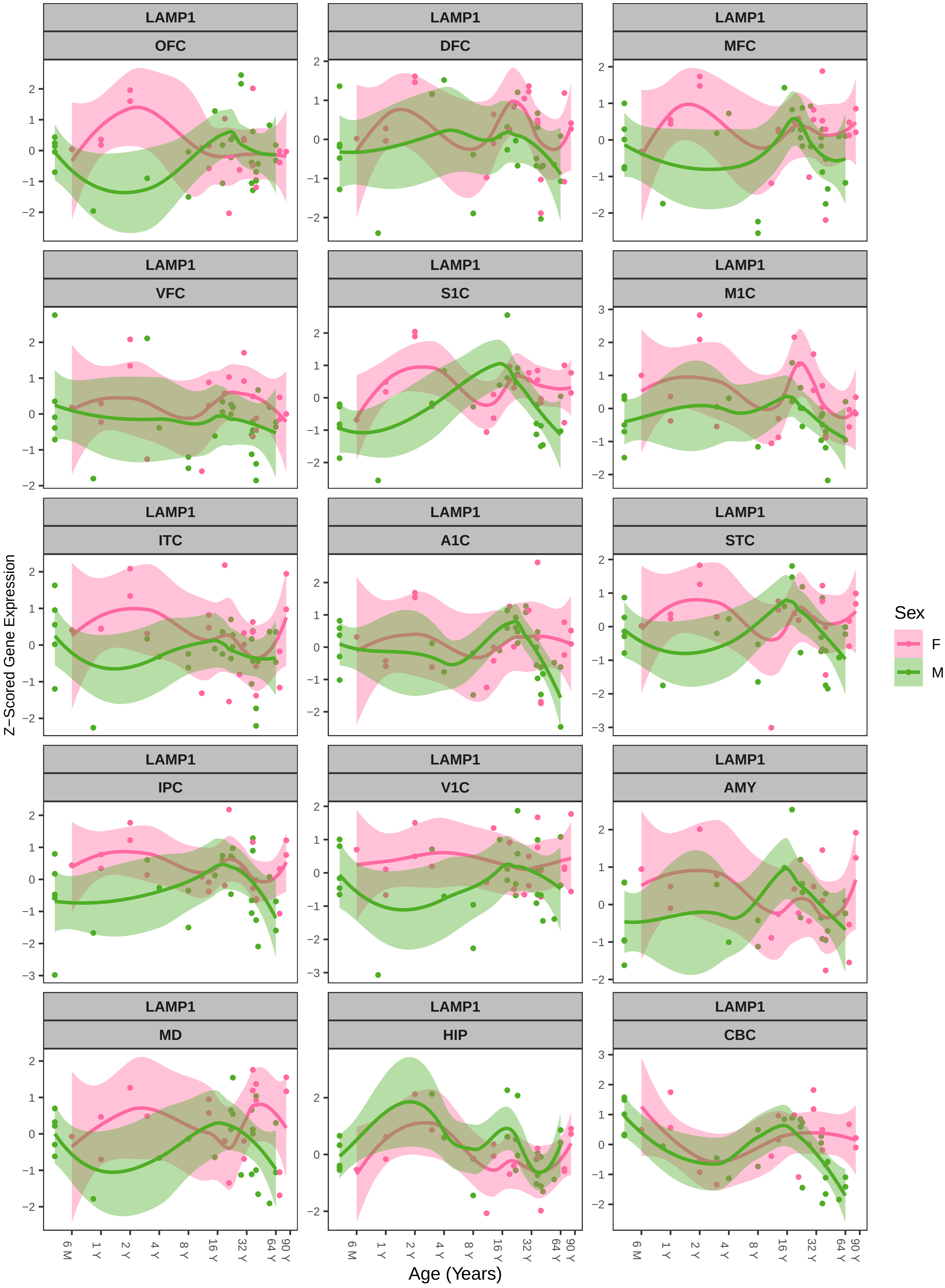
LAMP1
OFC
DFC
MFC
VFC
S1C
M1C
ITC
A1C
STC
IPC
V1C
AMY
MD
HIP
CBC
Z−Scored Gene Expression
Age (Years)
6 M
1 Y
2 Y
4 Y
8 Y
16 Y
32 Y
64 Y
90 Y
Sex
F
M

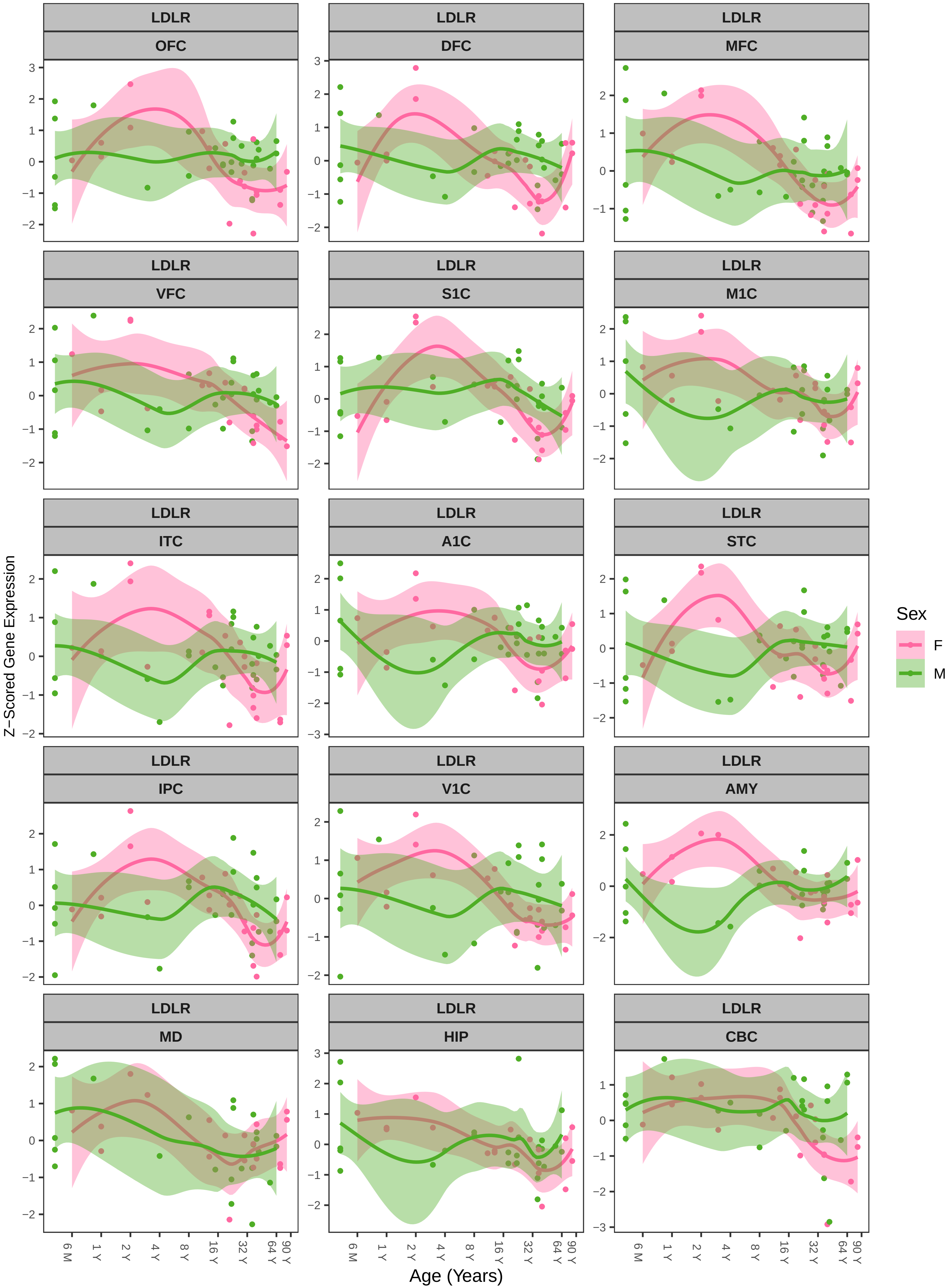
LDLR
OFC
DFC
MFC
VFC
S1C
M1C
ITC
A1C
STC
IPC
V1C
AMY
MD
HIP
CBC
Z−Scored Gene Expression
Age (Years)
6 M
1 Y
2 Y
4 Y
8 Y
16 Y
32 Y
64 Y
90 Y
Sex
F
M

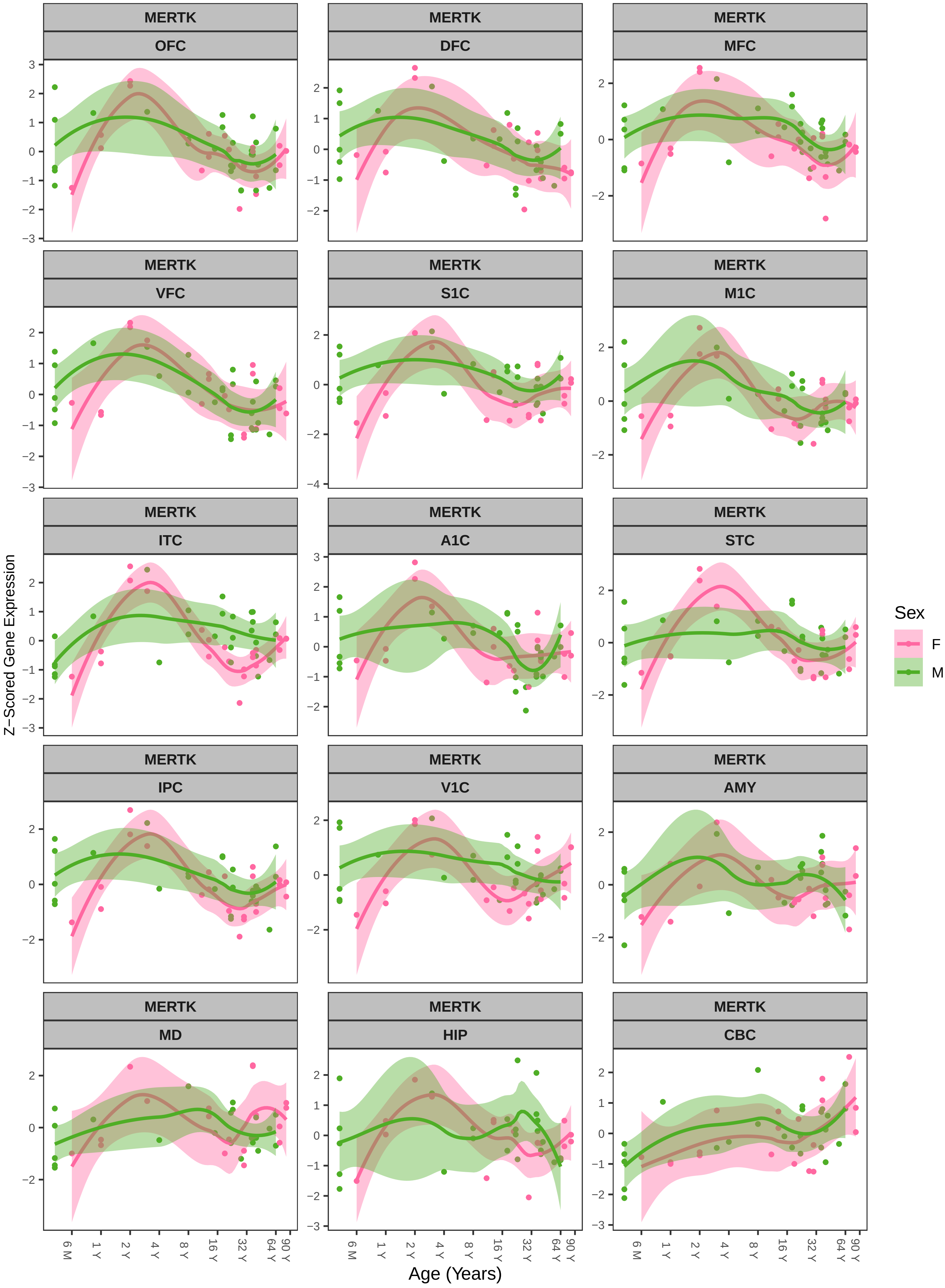
MERTK
OFC
DFC
MFC
VFC
S1C
M1C
ITC
A1C
STC
IPC
V1C
AMY
MD
HIP
CBC
Z−Scored Gene Expression
Age (Years)
6 M
1 Y
2 Y
4 Y
8 Y
16 Y
32 Y
64 Y
90 Y
Sex
F
M

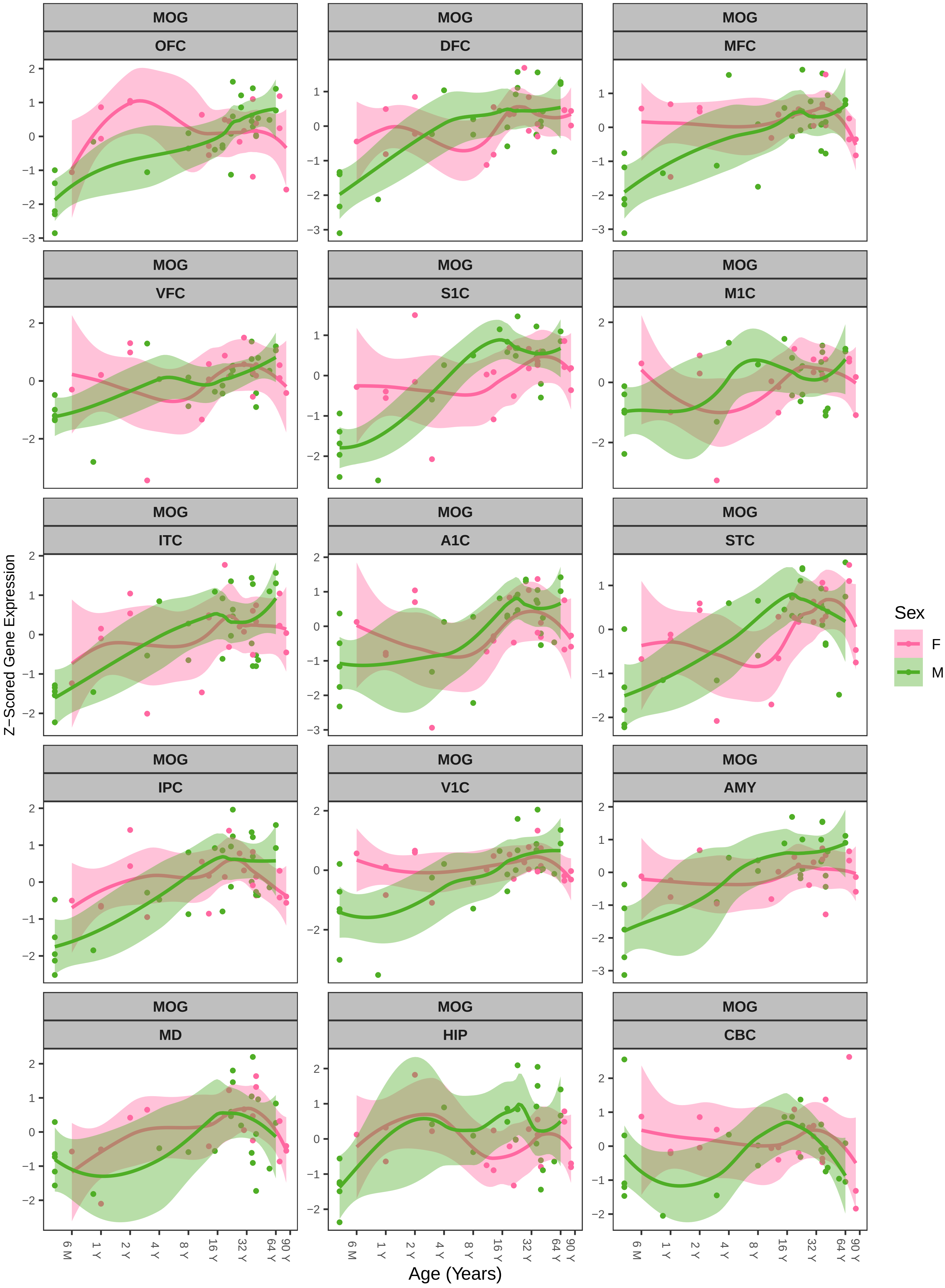

MOG
OFC
DFC
MFC
VFC
S1C
M1C
ITC
A1C
STC
IPC
V1C
AMY
MD
HIP
CBC
Z−Scored Gene Expression
Age (Years)
6 M
1 Y
2 Y
4 Y
8 Y
16 Y
32 Y
64 Y
90 Y
Sex
F
M

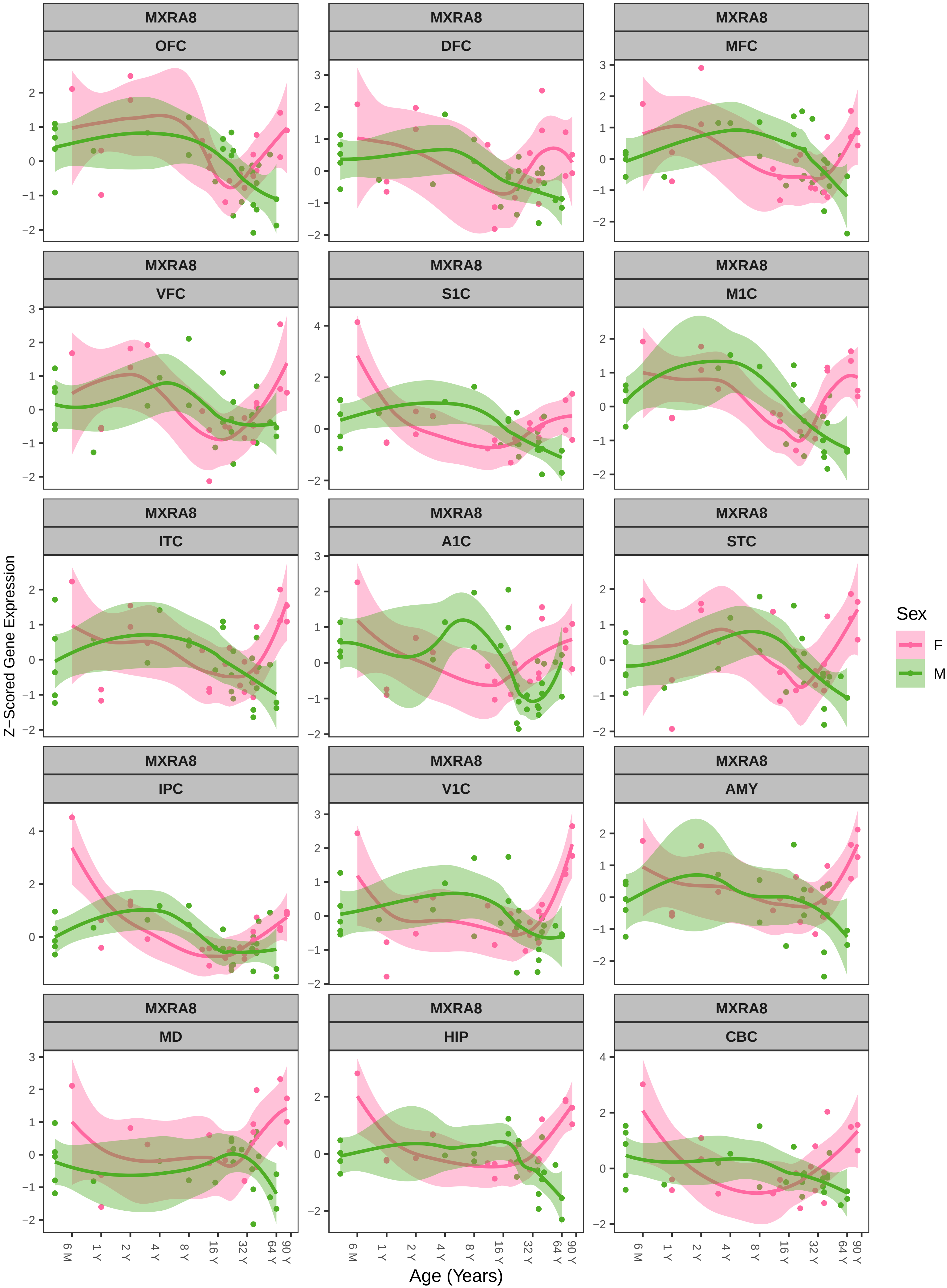
MXRA8
OFC
DFC
MFC
VFC
S1C
M1C
ITC
A1C
STC
IPC
V1C
AMY
MD
HIP
CBC
Z−Scored Gene Expression
Age (Years)
6 M
1 Y
2 Y
4 Y
8 Y
16 Y
32 Y
64 Y
90 Y
Sex
F
M

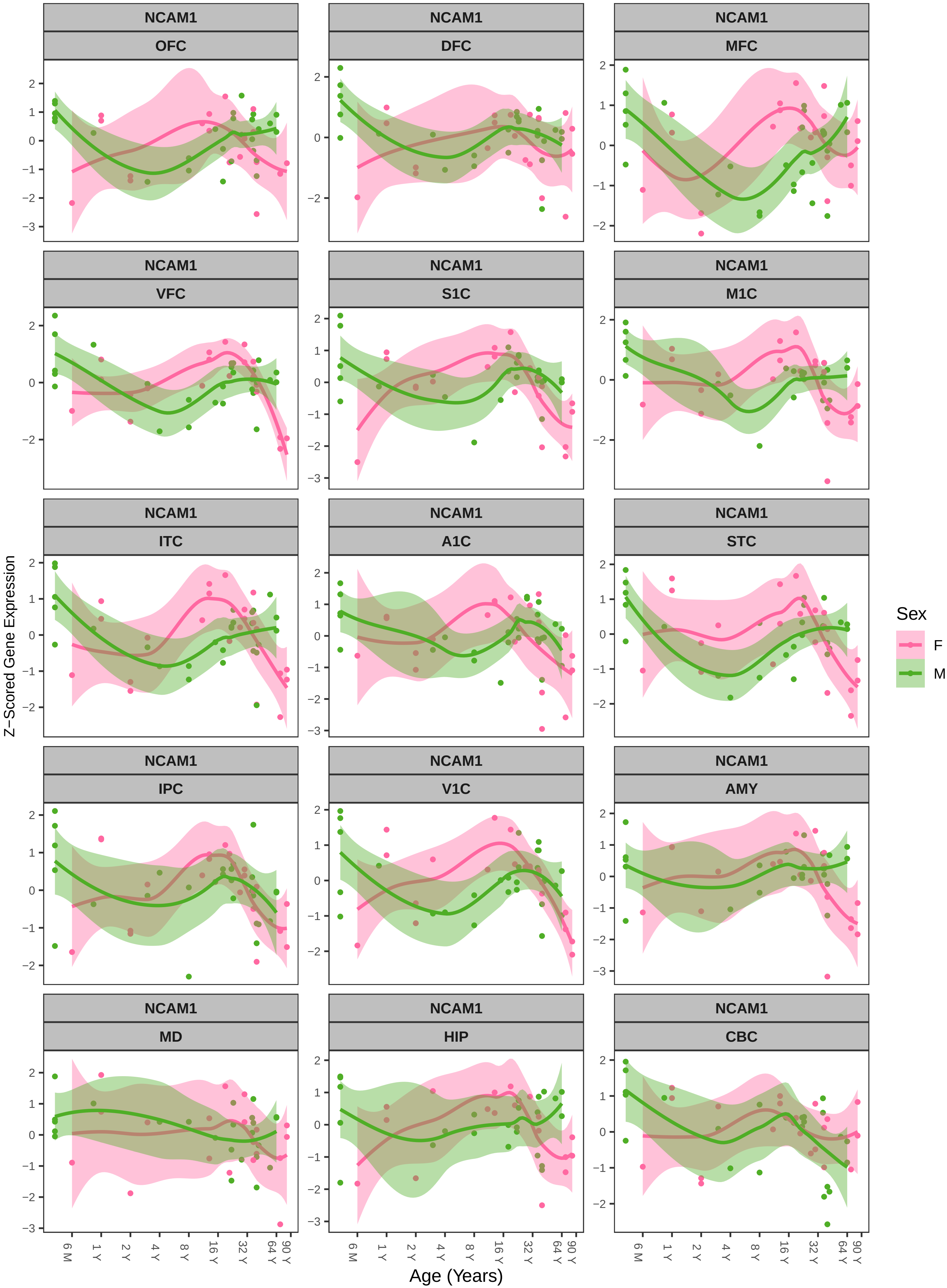

NCAM1
OFC
DFC
MFC
VFC
S1C
M1C
ITC
A1C
STC
IPC
V1C
AMY
MD
HIP
CBC
Z−Scored Gene Expression
Age (Years)
6 M
1 Y
2 Y
4 Y
8 Y
16 Y
32 Y
64 Y
90 Y
Sex
F
M

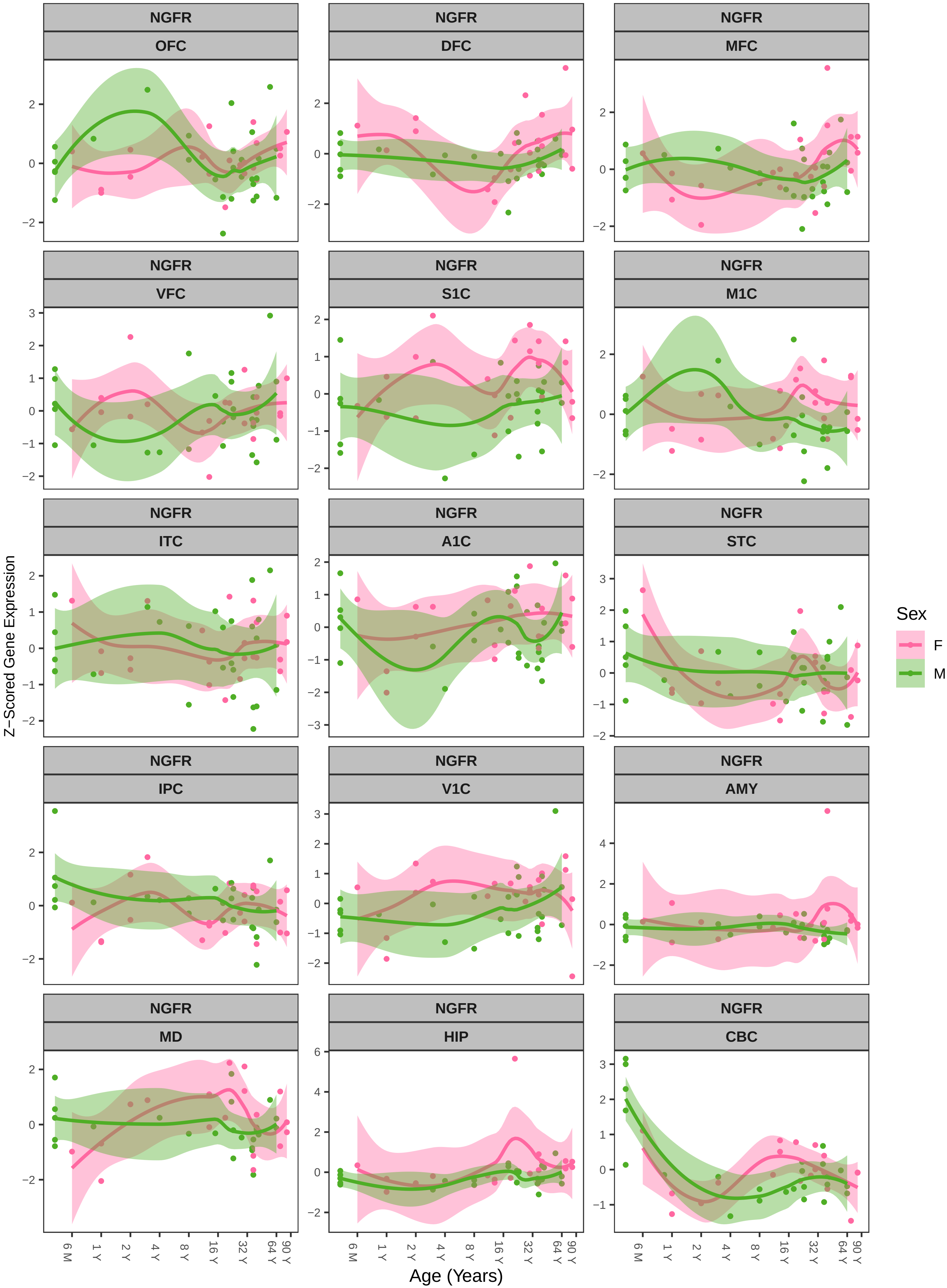
NGFR
OFC
DFC
MFC
VFC
S1C
M1C
ITC
A1C
STC
IPC
V1C
AMY
MD
HIP
CBC
Z−Scored Gene Expression
Age (Years)
6 M
1 Y
2 Y
4 Y
8 Y
16 Y
32 Y
64 Y
90 Y
Sex
F
M

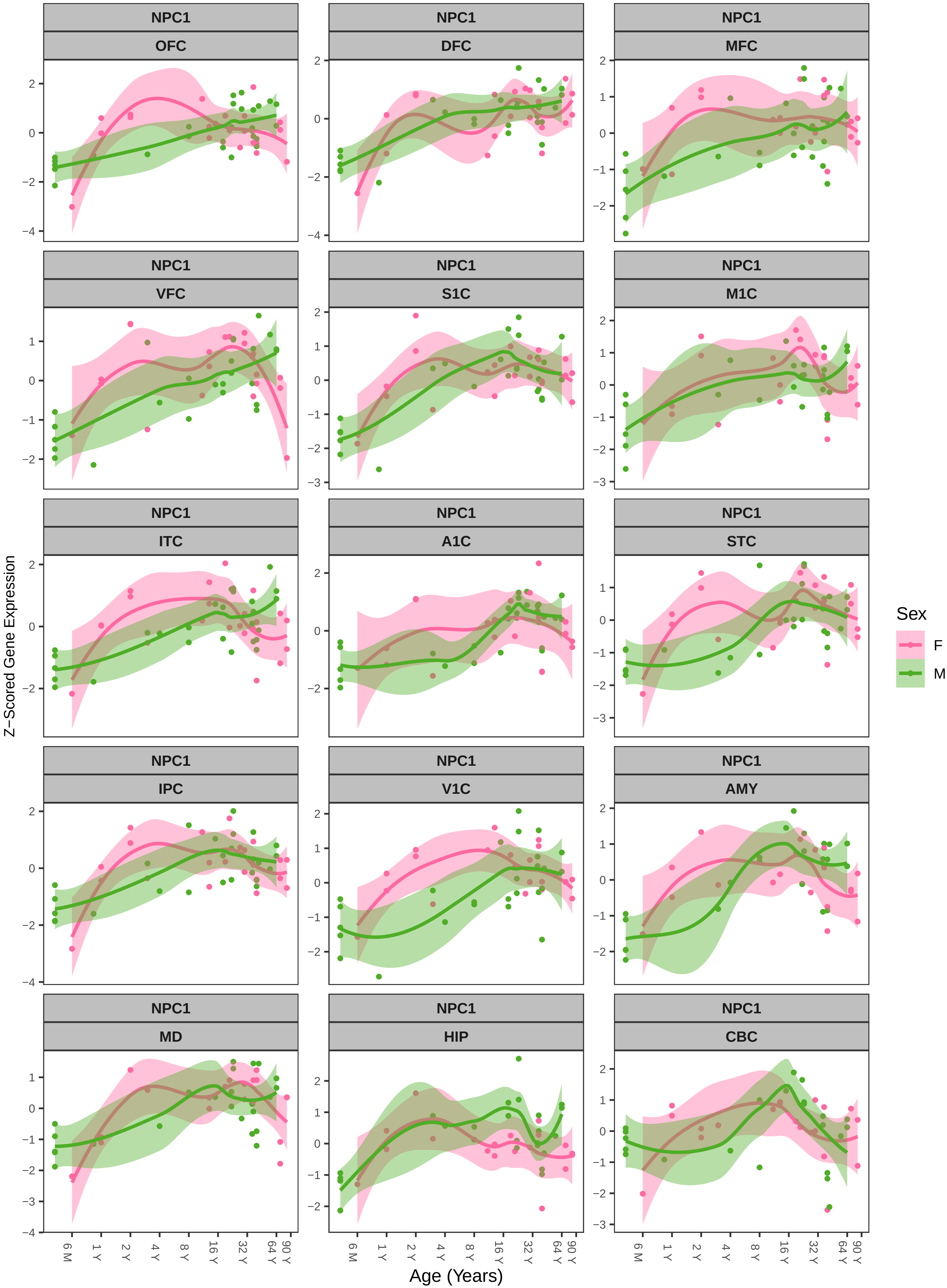
NPC1
OFC
DFC
MFC
VFC
S1C
M1C
ITC
A1C
STC
IPC
V1C
AMY
MD
HIP
CBC
Z−Scored Gene Expression
Age (Years)
6 M
1 Y
2 Y
4 Y
8 Y
16 Y
32 Y
64 Y
90 Y
Sex
F
M

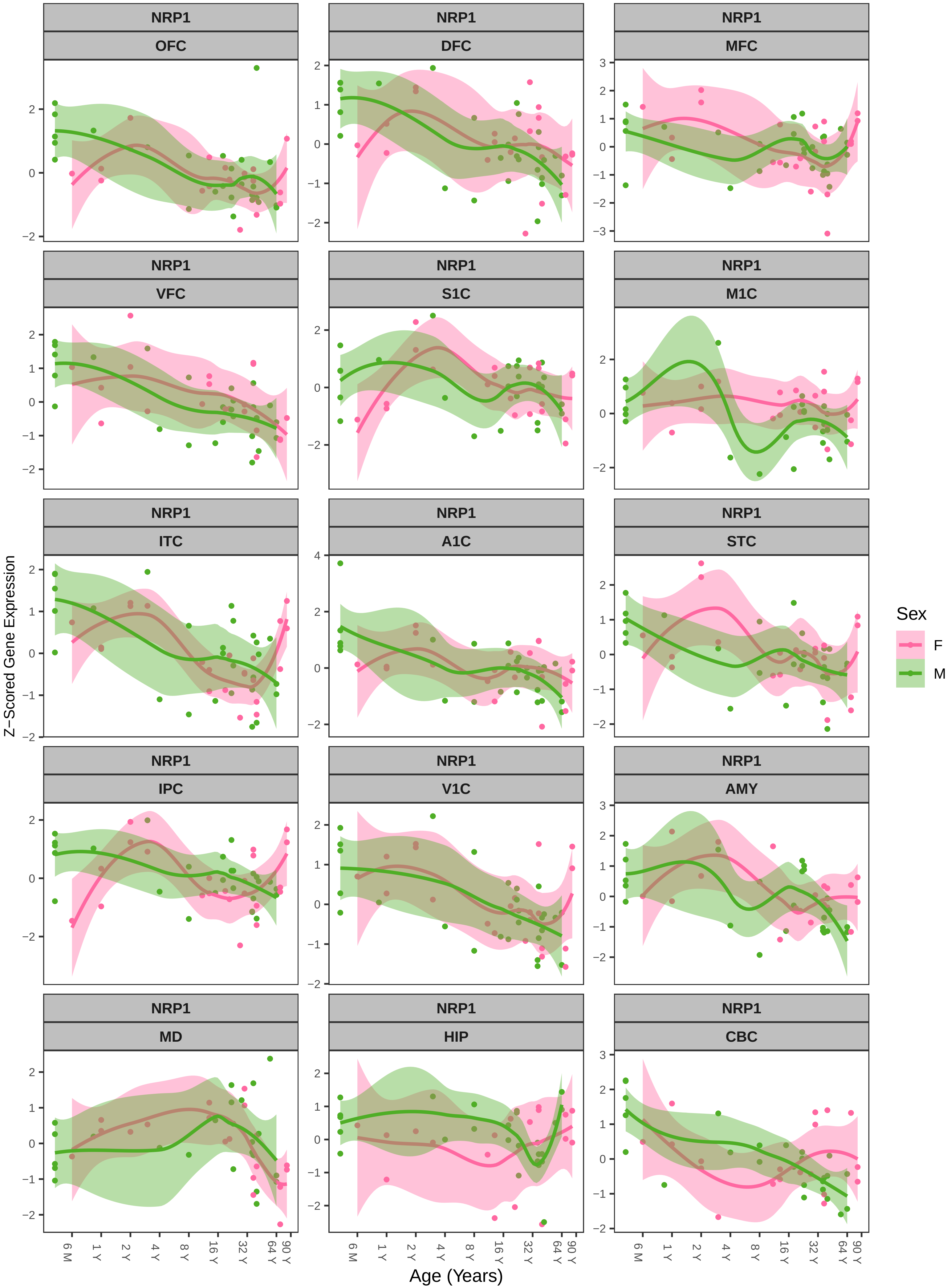

NRP1
OFC
DFC
MFC
VFC
S1C
M1C
ITC
A1C
STC
IPC
V1C
AMY
MD
HIP
CBC
Z−Scored Gene Expression
Age (Years)
6 M
1 Y
2 Y
4 Y
8 Y
16 Y
32 Y
64 Y
90 Y
Sex
F
M

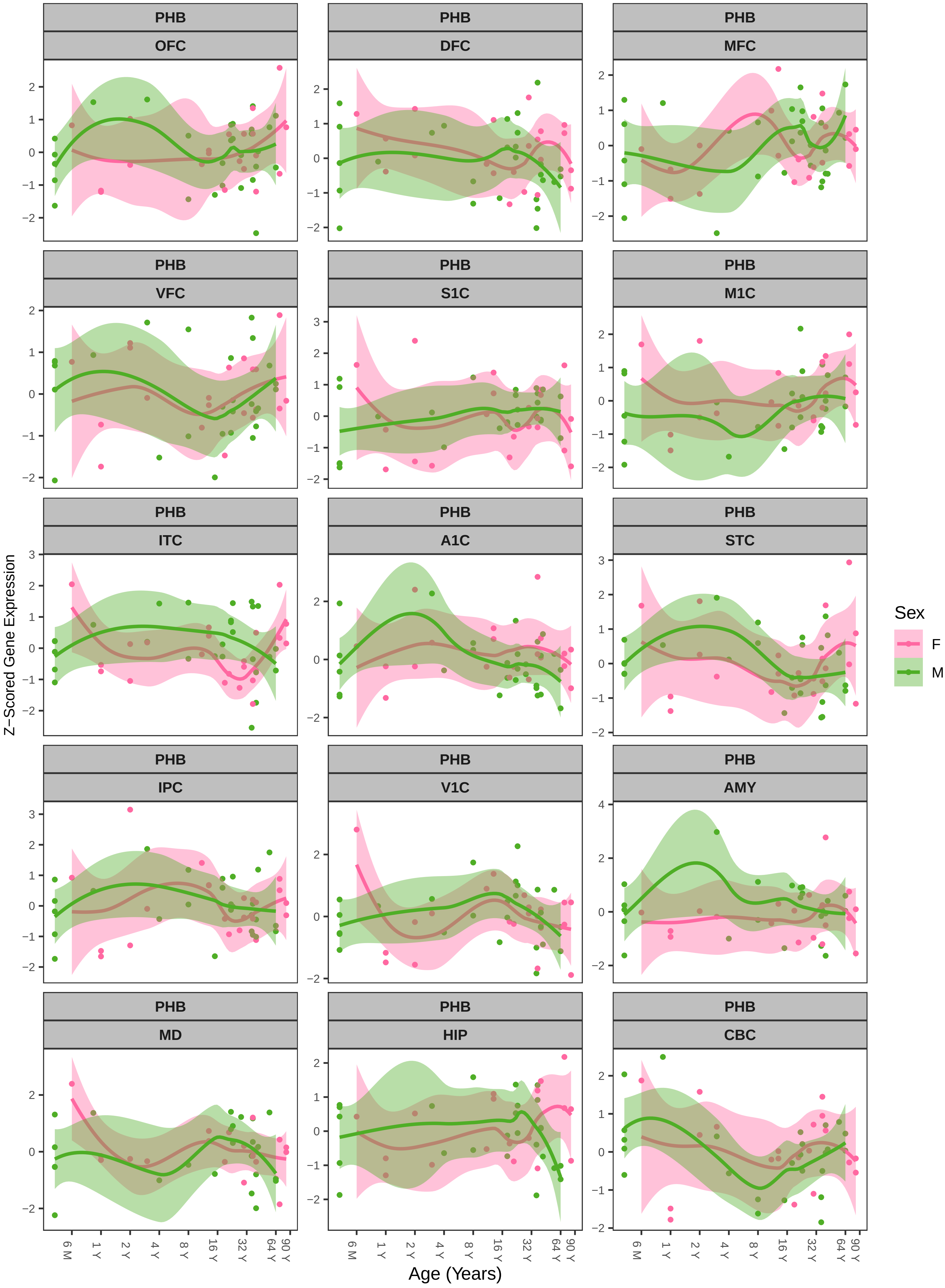
PHB
OFC
DFC
MFC
VFC
S1C
M1C
ITC
A1C
STC
IPC
V1C
AMY
MD
HIP
CBC
Z−Scored Gene Expression
Age (Years)
6 M
1 Y
2 Y
4 Y
8 Y
16 Y
32 Y
64 Y
90 Y
Sex
F
M

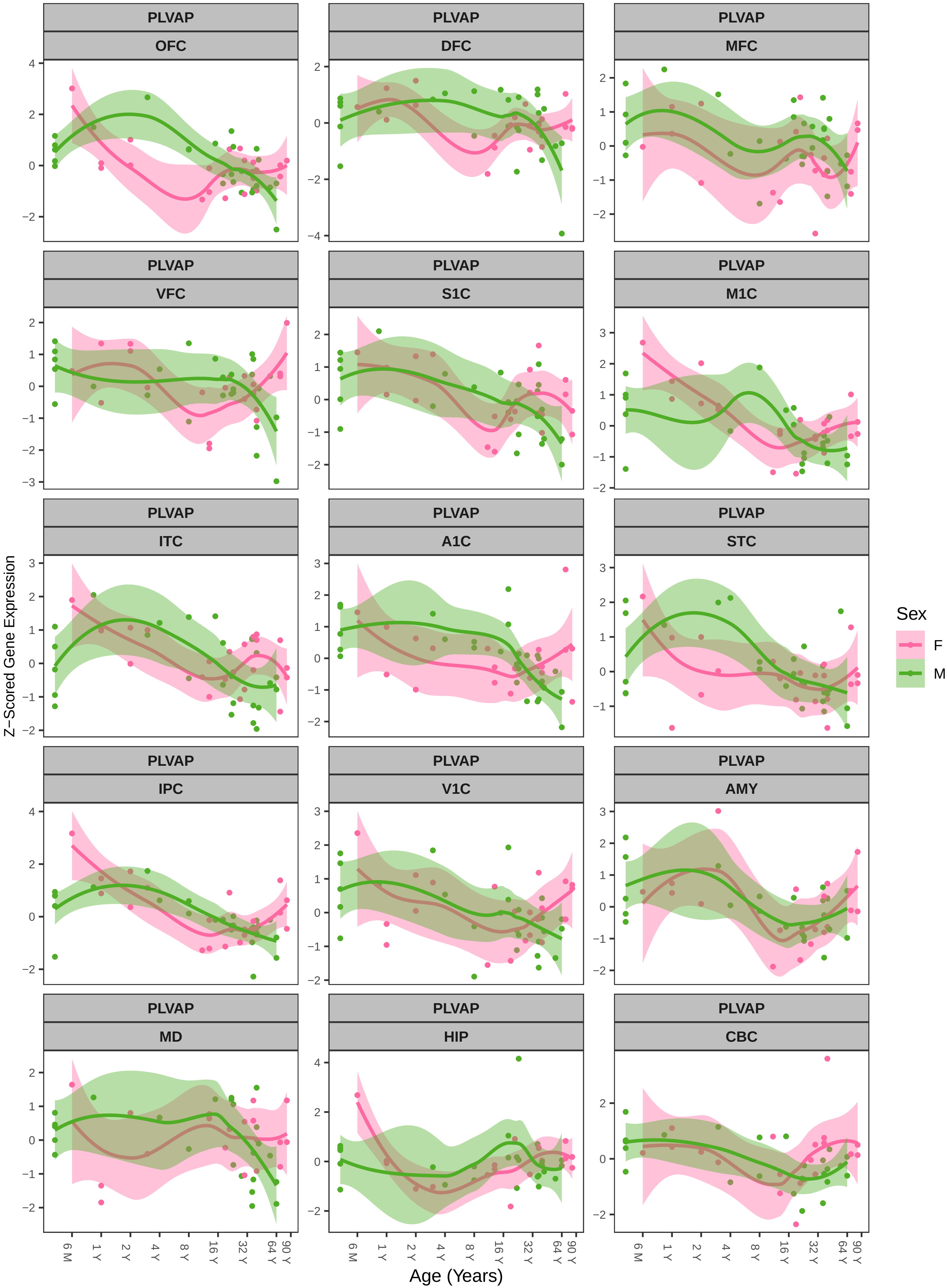
PLVAP
OFC
DFC
MFC
VFC
S1C
M1C
ITC
A1C
STC
IPC
V1C
AMY
MD
HIP
CBC
Z−Scored Gene Expression
Age (Years)
6 M
1 Y
2 Y
4 Y
8 Y
16 Y
32 Y
64 Y
90 Y
Sex
F
M

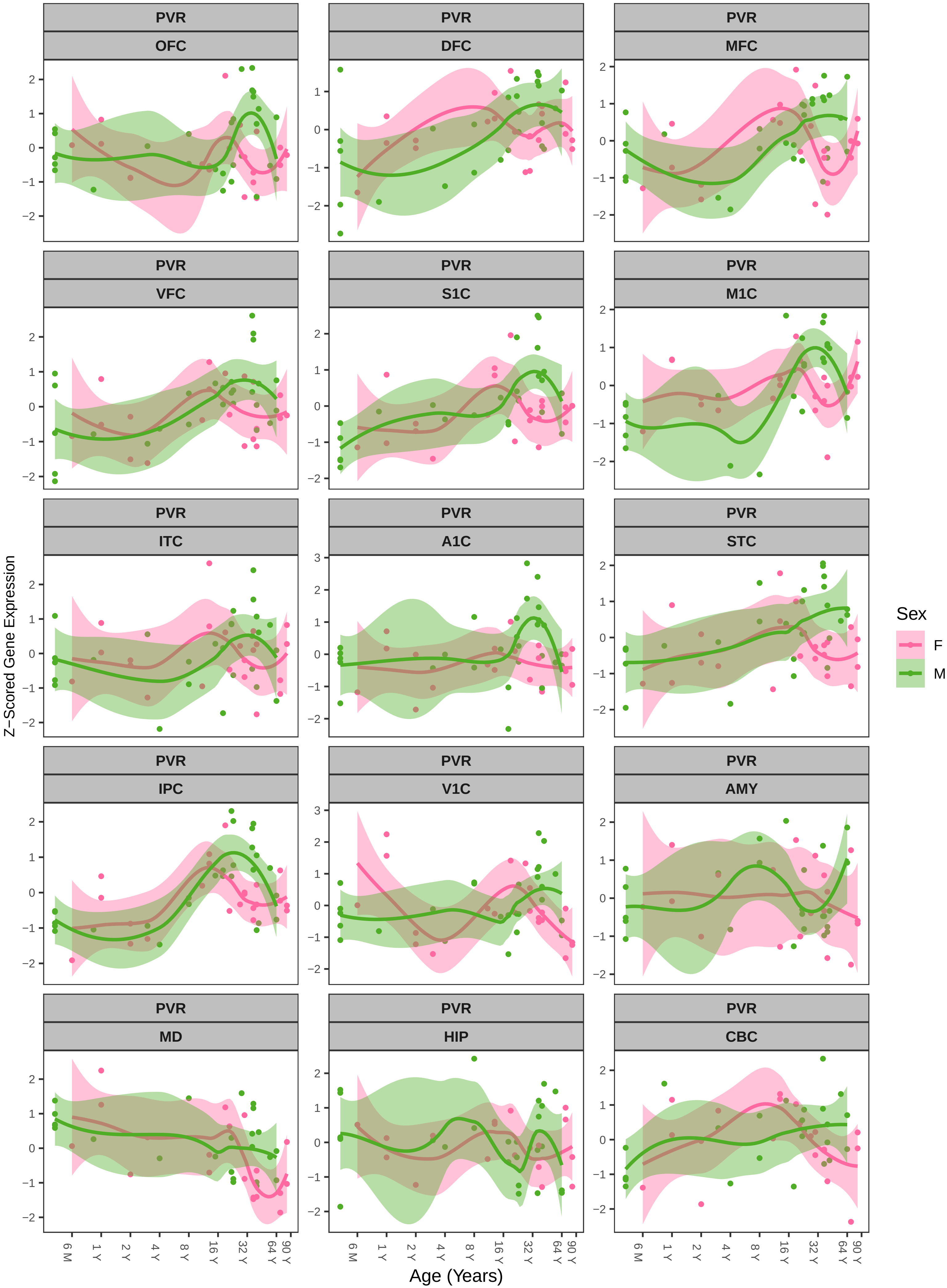
PVR
OFC
DFC
MFC
VFC
S1C
M1C
ITC
A1C
STC
IPC
V1C
AMY
MD
HIP
CBC
Z−Scored Gene Expression
Age (Years)
6 M
1 Y
2 Y
4 Y
8 Y
16 Y
32 Y
64 Y
90 Y
Sex
F
M

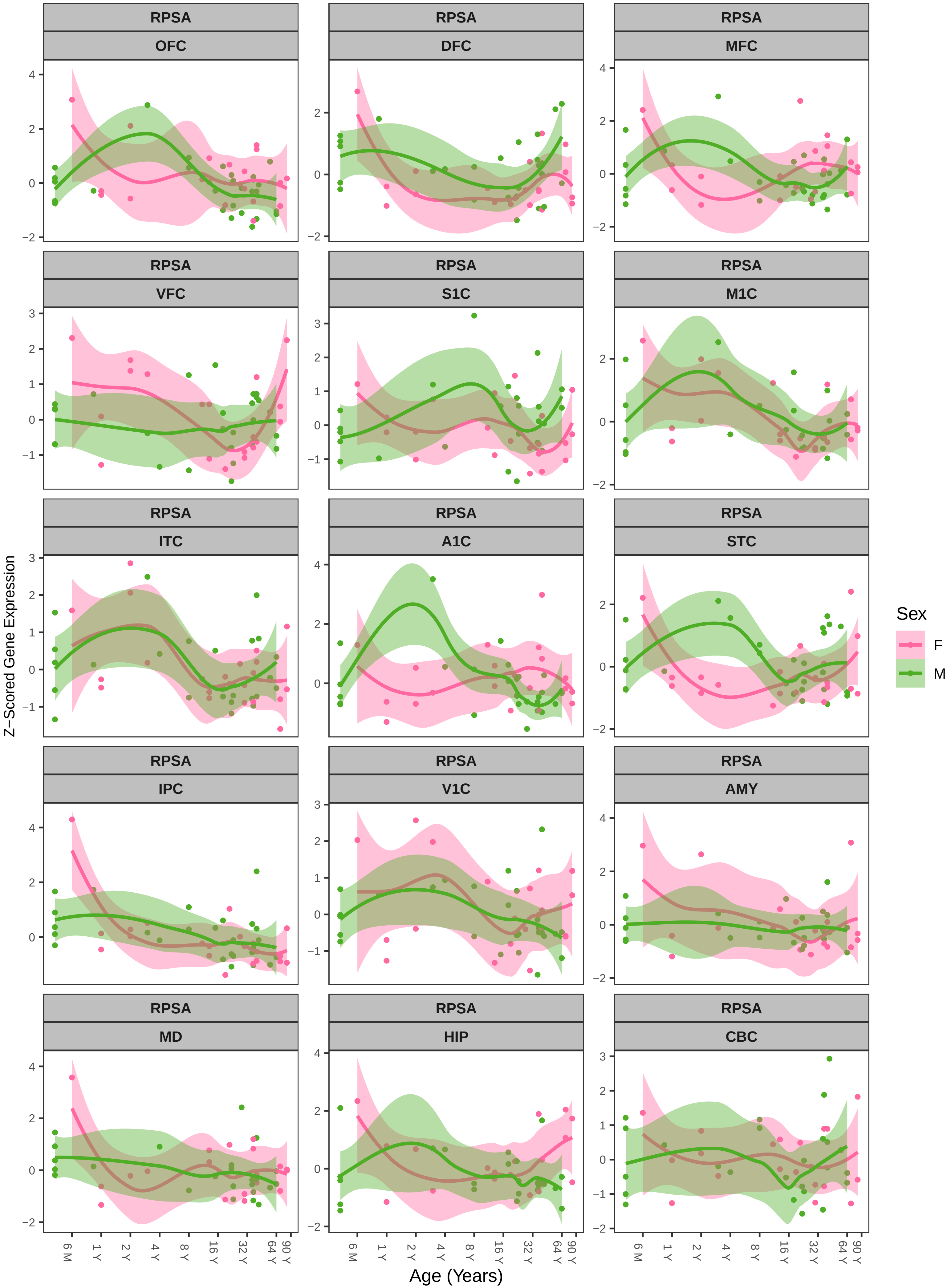
RPSA
OFC
DFC
MFC
VFC
S1C
M1C
ITC
A1C
STC
IPC
V1C
AMY
MD
HIP
CBC
Z−Scored Gene Expression
Age (Years)
6 M
1 Y
2 Y
4 Y
8 Y
16 Y
32 Y
64 Y
90 Y
Sex
F
M

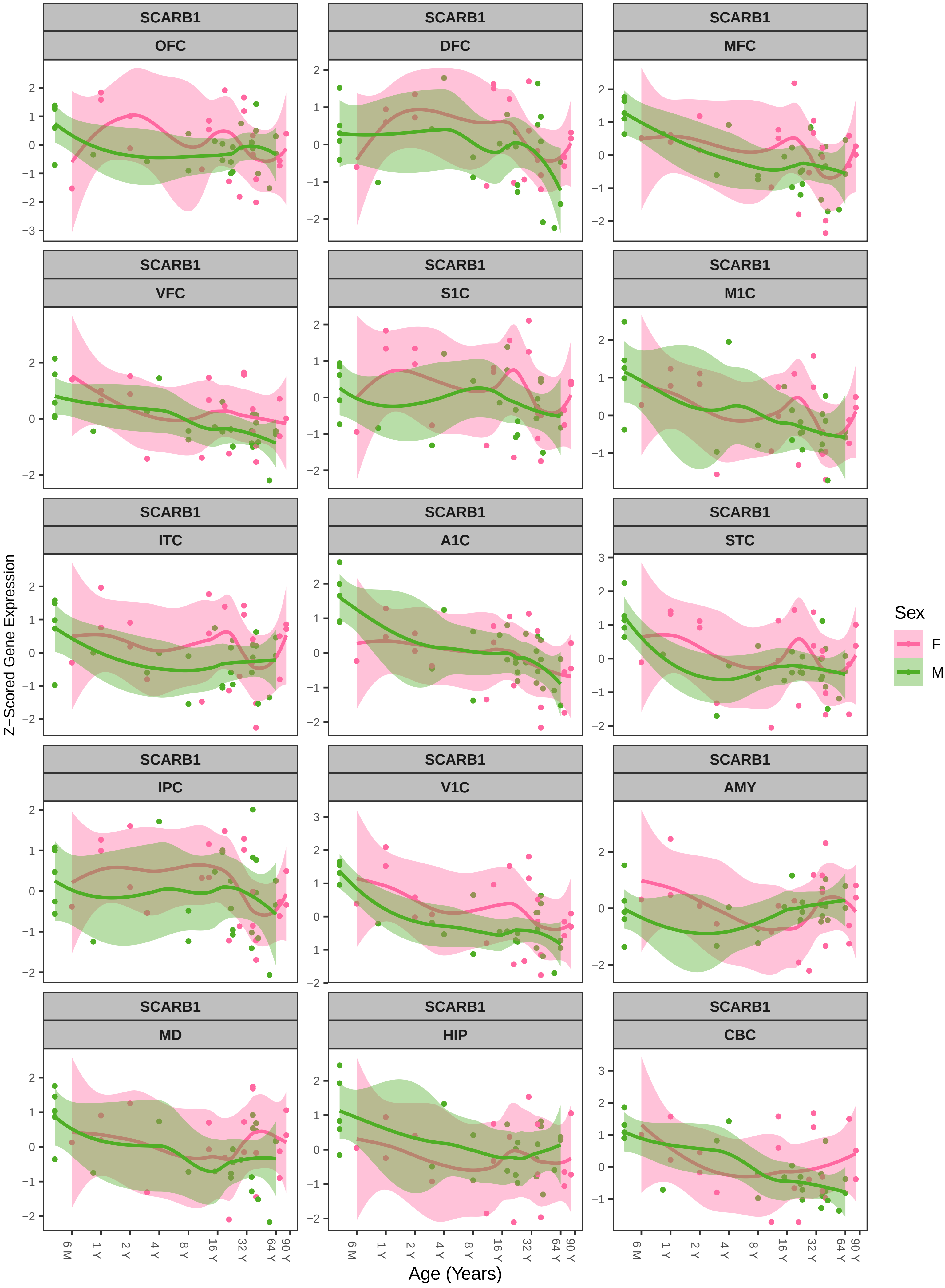

SCARB1
OFC
DFC
MFC
VFC
S1C
M1C
ITC
A1C
STC
IPC
V1C
AMY
MD
HIP
CBC
Z−Scored Gene Expression
Age (Years)
6 M
1 Y
2 Y
4 Y
8 Y
16 Y
32 Y
64 Y
90 Y
Sex
F
M

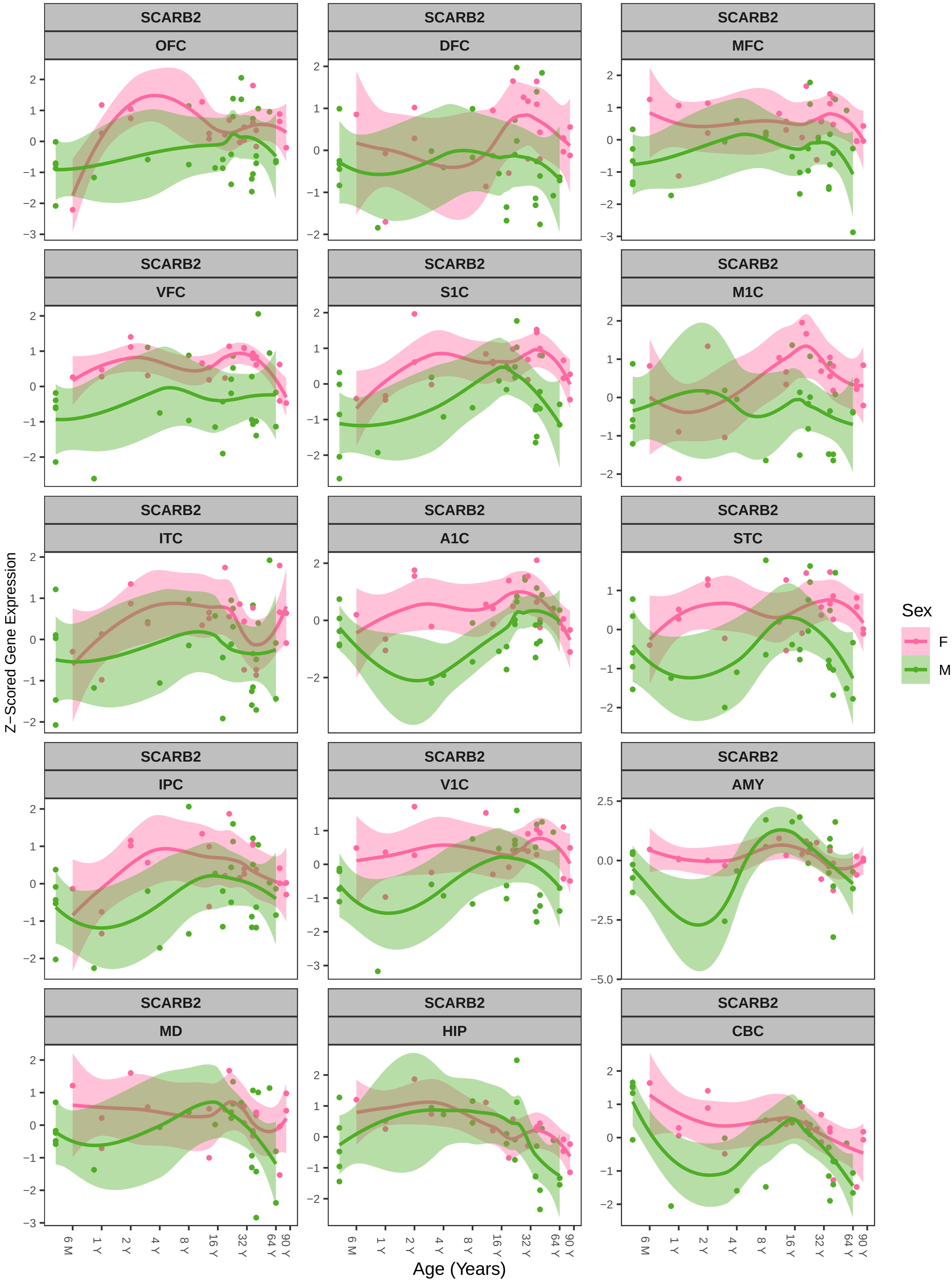

SCARB2
OFC
DFC
MFC
VFC
S1C
M1C
ITC
A1C
STC
IPC
V1C
AMY
MD
HIP
CBC
Z−Scored Gene Expression
Age (Years)
6 M
1 Y
2 Y
4 Y
8 Y
16 Y
32 Y
64 Y
90 Y
Sex
F
M

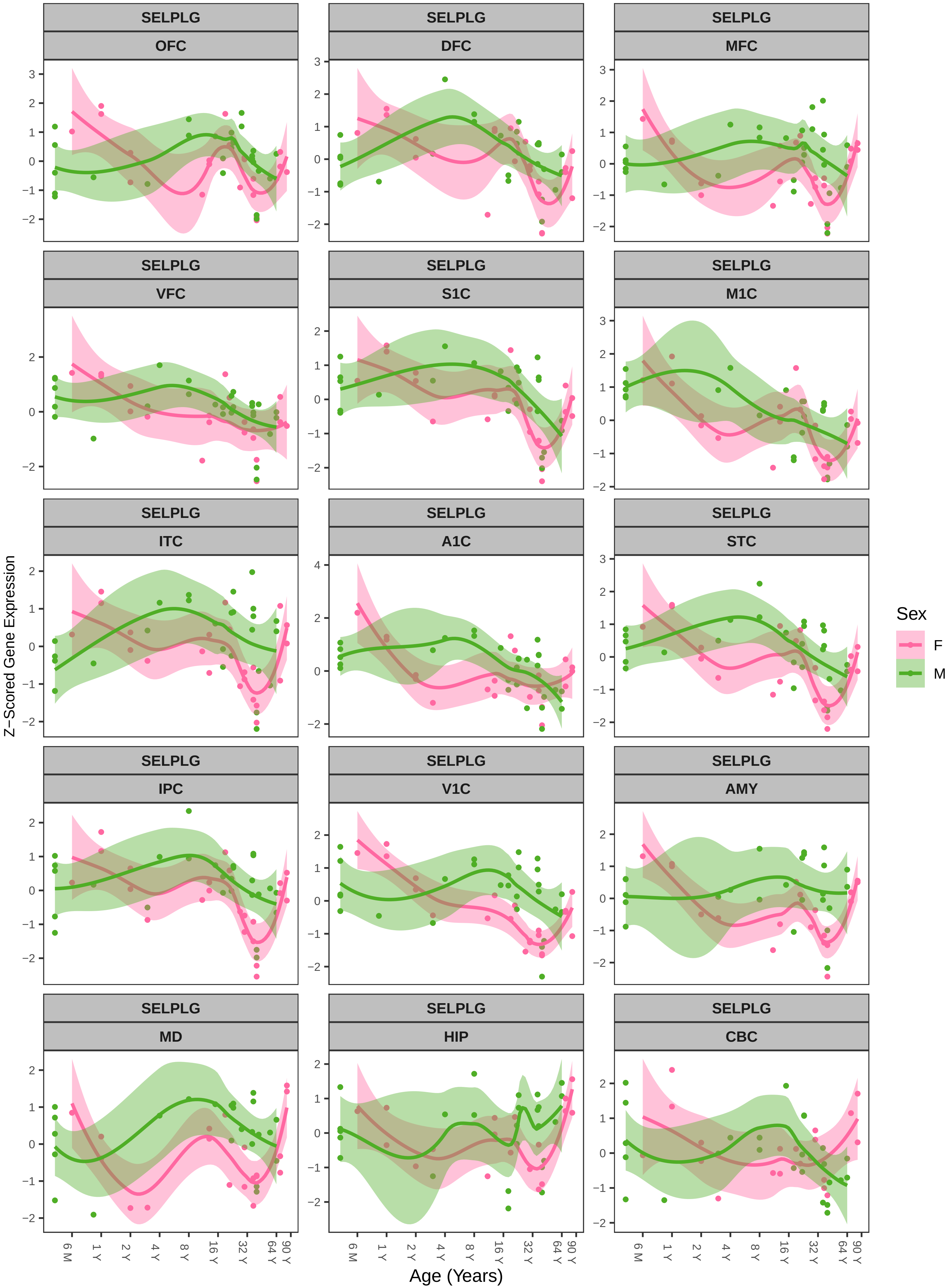

SELPLG
OFC
DFC
MFC
VFC
S1C
M1C
ITC
A1C
STC
IPC
V1C
AMY
MD
HIP
CBC
Z−Scored Gene Expression
Age (Years)
6 M
1 Y
2 Y
4 Y
8 Y
16 Y
32 Y
64 Y
90 Y
Sex
F
M

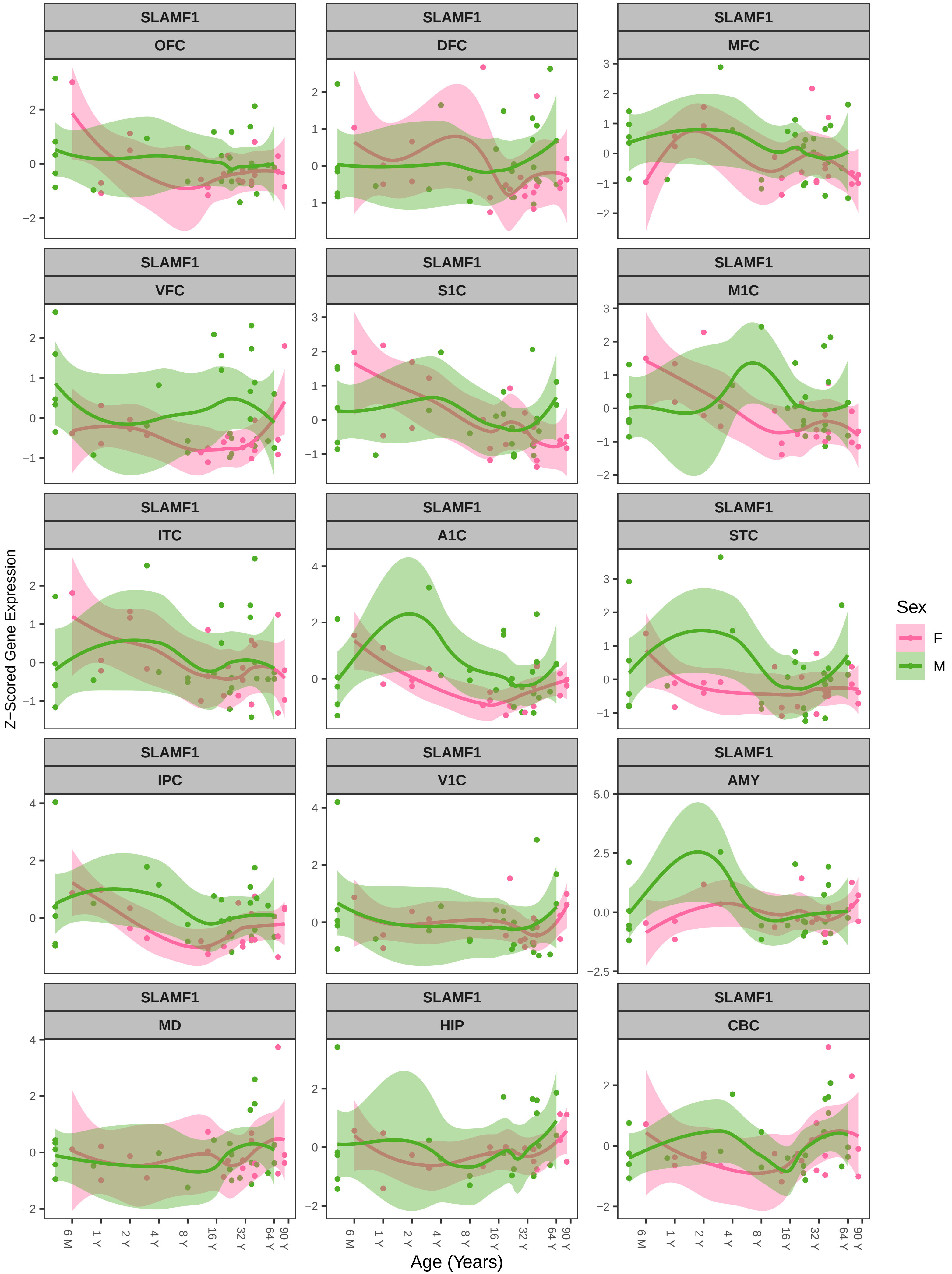
SLAMF1
OFC
DFC
MFC
VFC
S1C
M1C
ITC
A1C
STC
IPC
V1C
AMY
MD
HIP
CBC
Z−Scored Gene Expression
Age (Years)
6 M
1 Y
2 Y
4 Y
8 Y
16 Y
32 Y
64 Y
90 Y
Sex
F
M

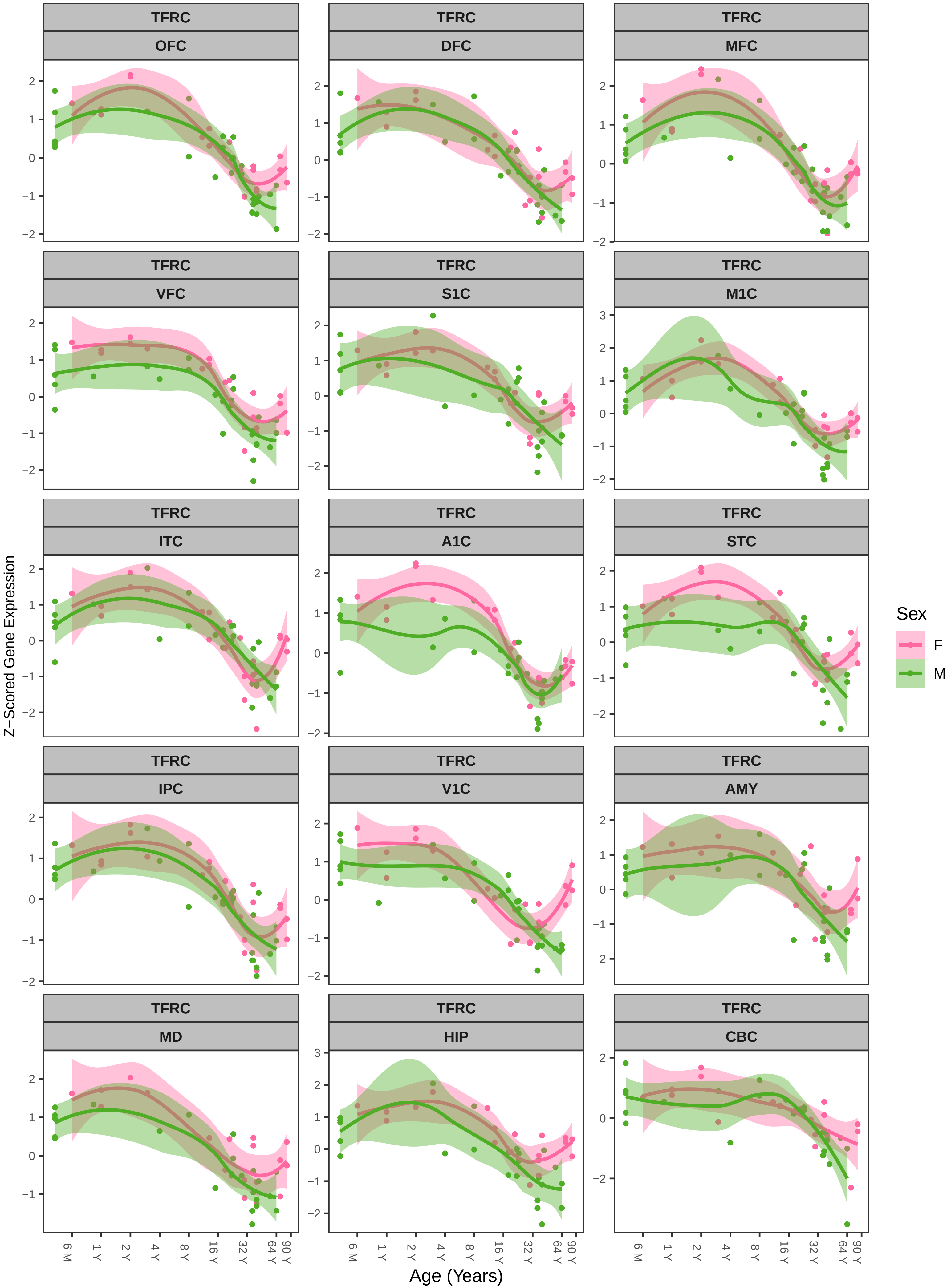

TFRC
OFC
DFC
MFC
VFC
S1C
M1C
ITC
A1C
STC
IPC
V1C
AMY
MD
HIP
CBC
Z−Scored Gene Expression
Age (Years)
6 M
1 Y
2 Y
4 Y
8 Y
16 Y
32 Y
64 Y
90 Y
Sex
F
M

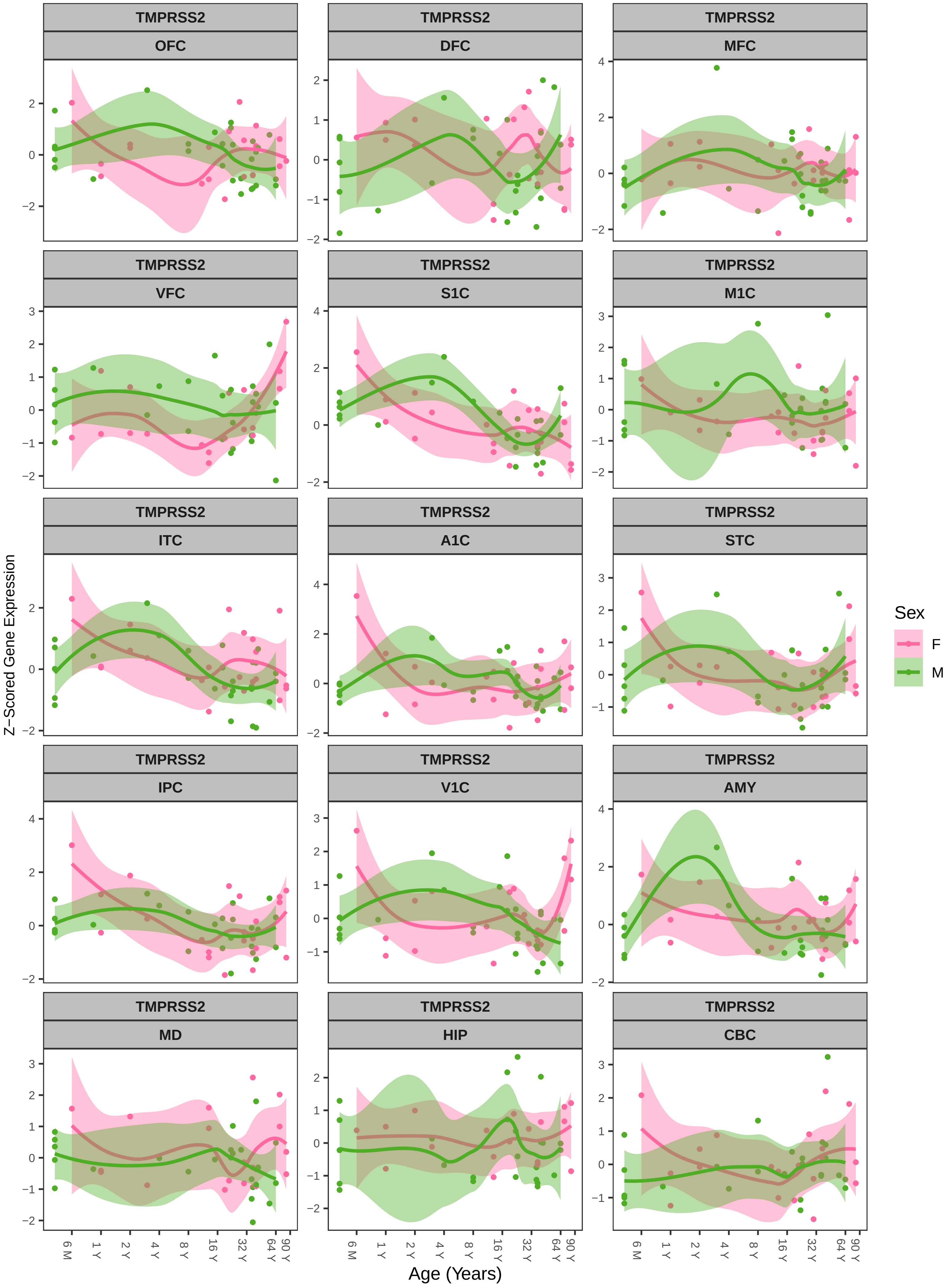
TMPRSS2
OFC
DFC
MFC
VFC
S1C
M1C
ITC
A1C
STC
IPC
V1C
AMY
MD
HIP
CBC
Z−Scored Gene Expression
Age (Years)
6 M
1 Y
2 Y
4 Y
8 Y
16 Y
32 Y
64 Y
90 Y
Sex
F
M

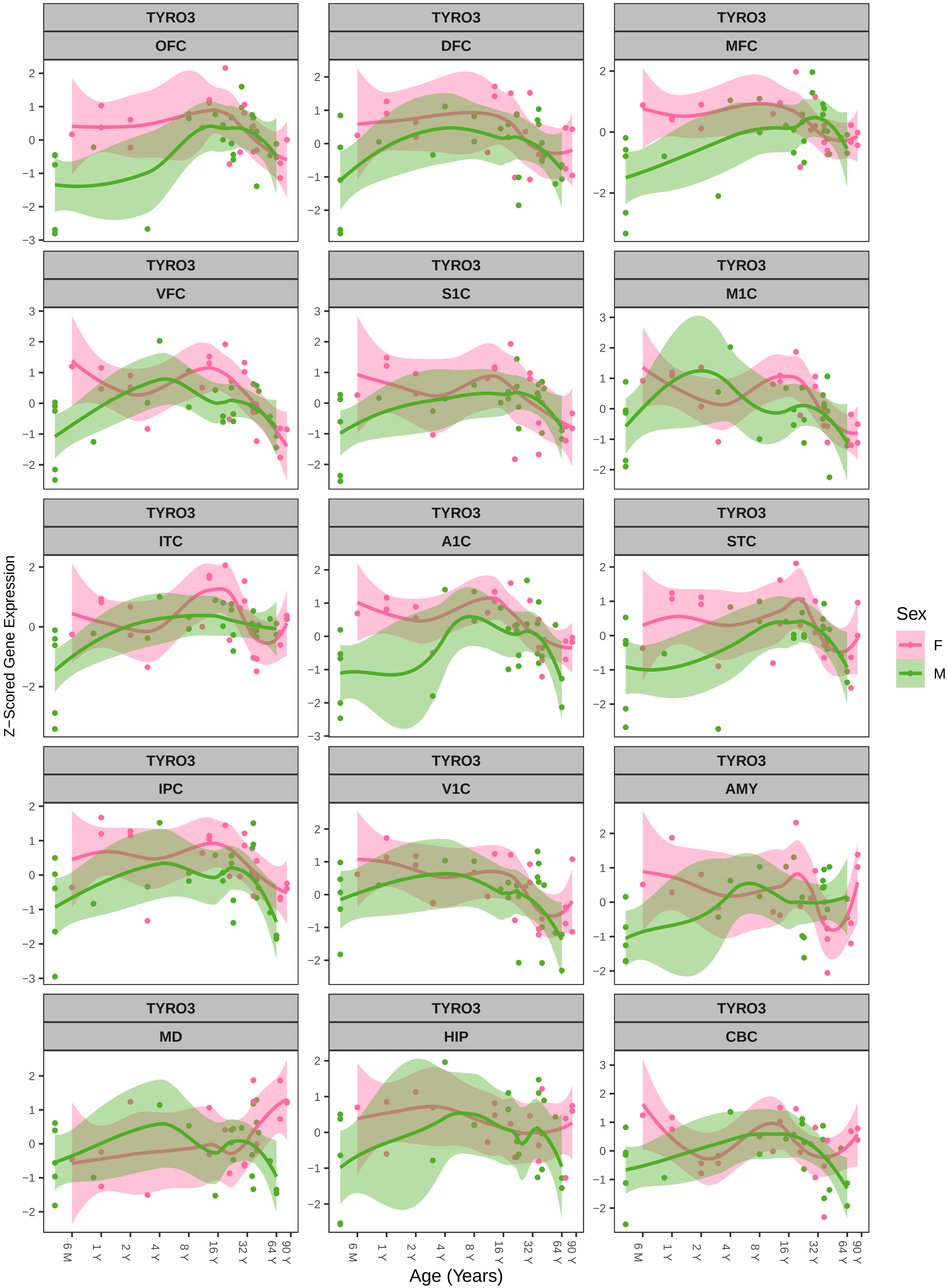
TYRO3
OFC
DFC
MFC
VFC
S1C
M1C
ITC
A1C
STC
IPC
V1C
AMY
MD
HIP
CBC
Z−Scored Gene Expression
Age (Years)
6 M
1 Y
2 Y
4 Y
8 Y
16 Y
32 Y
64 Y
90 Y
Sex
F
M

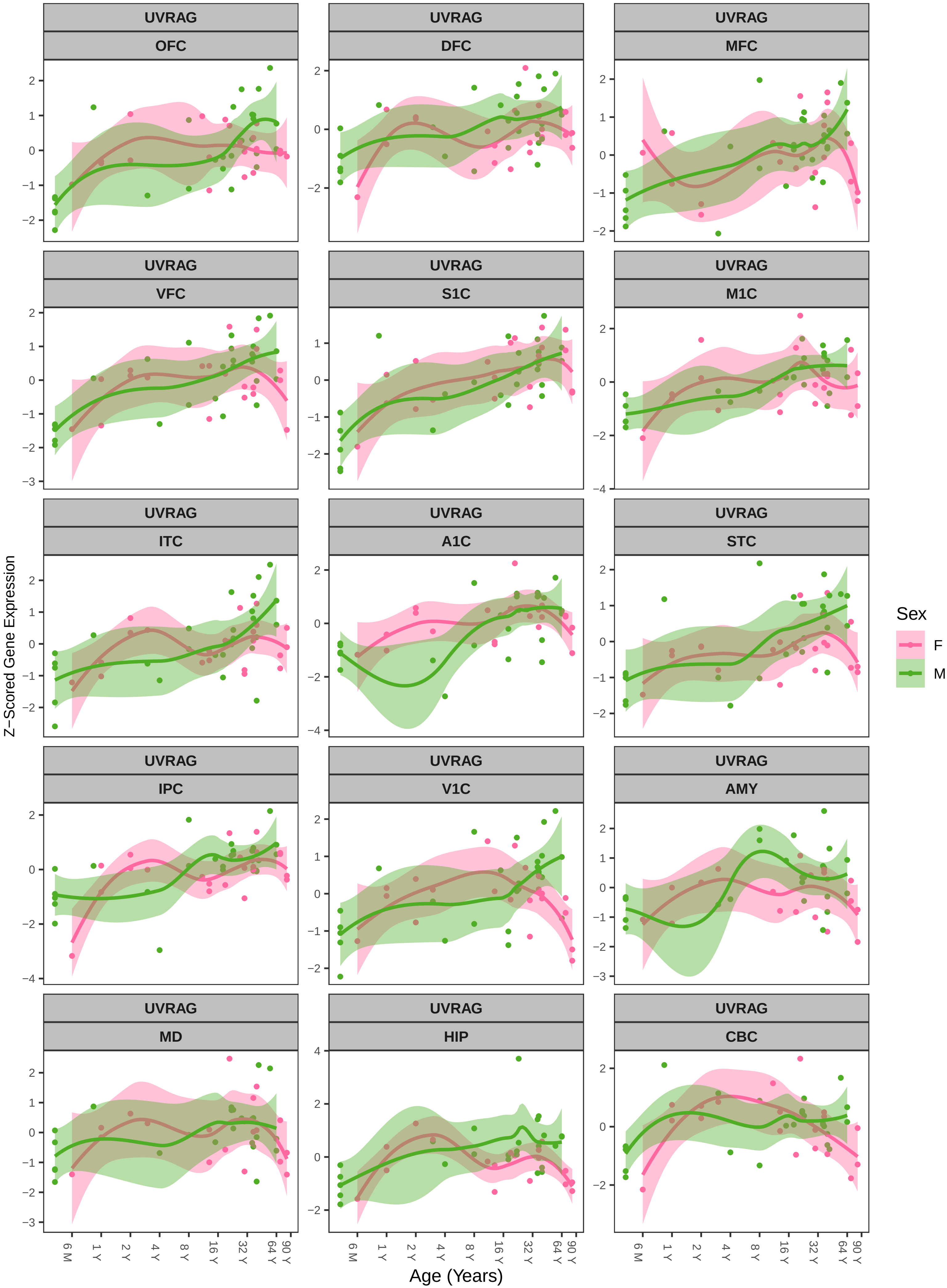

UVRAG
OFC
DFC
MFC
VFC
S1C
M1C
ITC
A1C
STC
IPC
V1C
AMY
MD
HIP
CBC
Z−Scored Gene Expression
Age (Years)
6 M
1 Y
2 Y
4 Y
8 Y
16 Y
32 Y
64 Y
90 Y
Sex
F
M

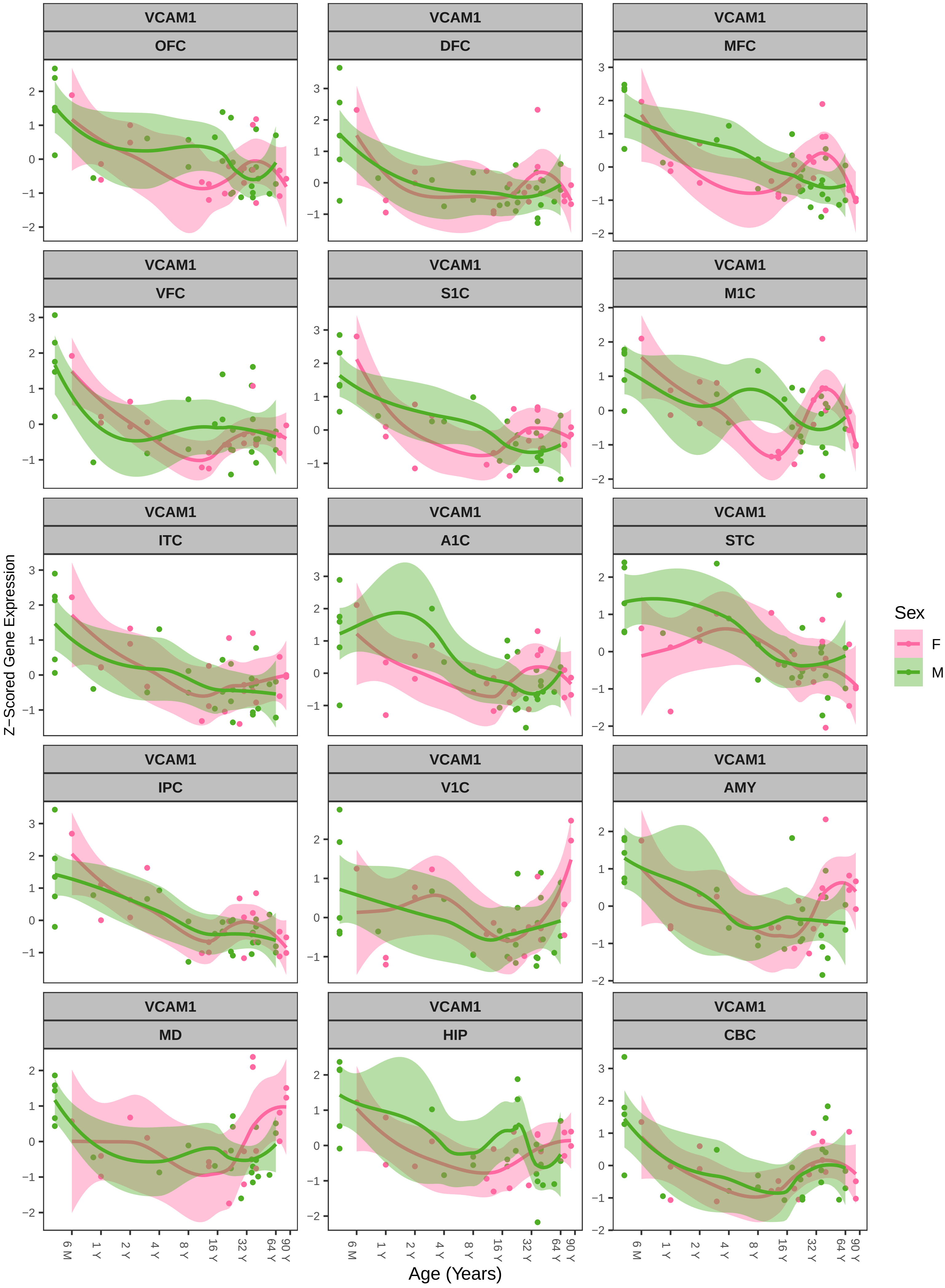

VCAM1
OFC
DFC
MFC
VFC
S1C
M1C
ITC
A1C
STC
IPC
V1C
AMY
MD
HIP
CBC
Z−Scored Gene Expression
Age (Years)
6 M
1 Y
2 Y
4 Y
8 Y
16 Y
32 Y
64 Y
90 Y
Sex
F
M
