# Supplementary material for: Sex- and age-specific development of ssRNA virus receptor expression in the human brain
Source: PLoS One. 2026 Oct 29;21(10):e0360526. doi: 10.1371/journal.pone.0360526 (PMC13718946; doi:10.1371/journal.pone.0360526)
Supplement: Supplementary file 2 [file pone.0360526.s002.pdf]

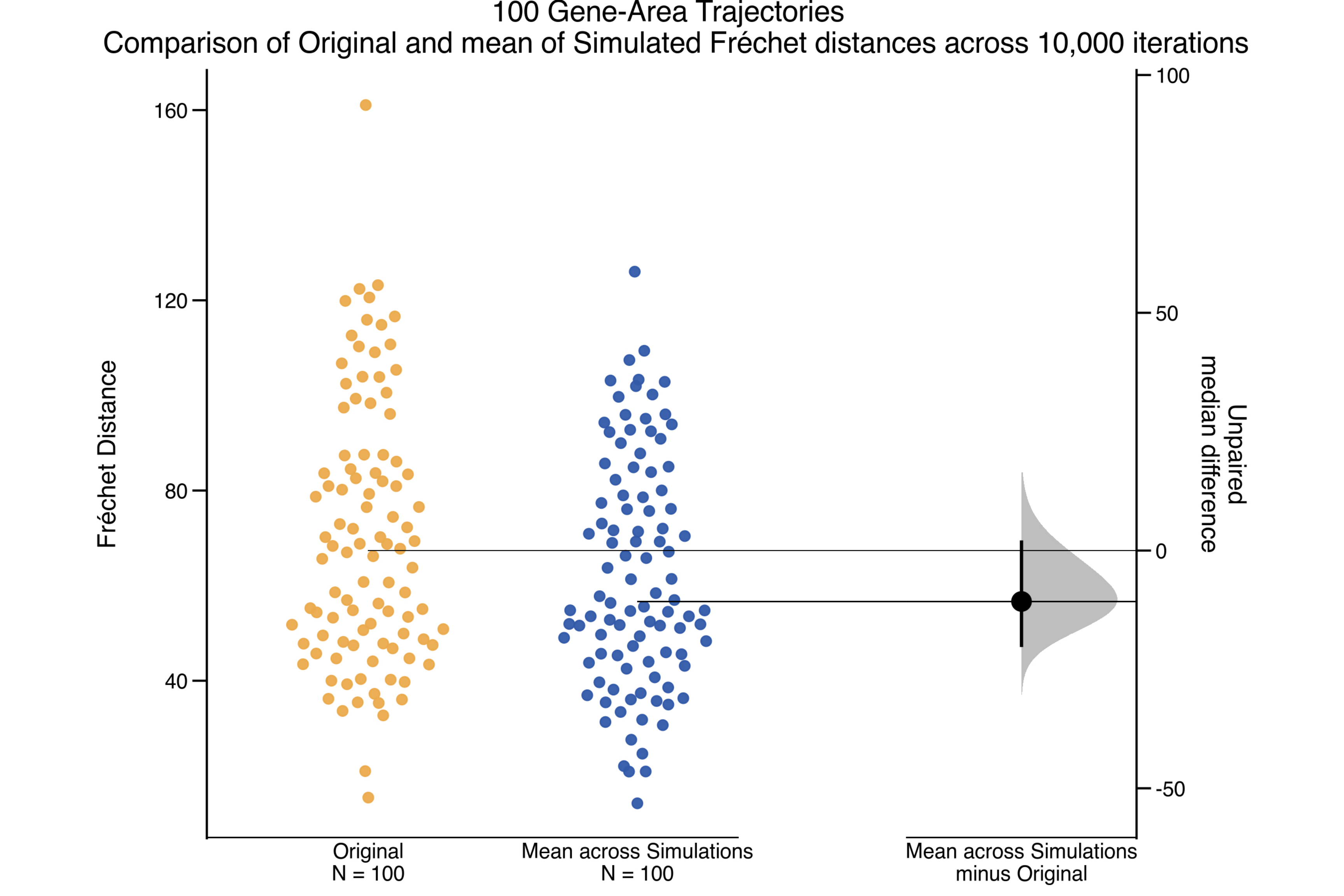
100 Gene-Area Trajectories
Comparison of Original and mean of Simulated Fréchet distances across 10,000 iterations
Fréchet Distance
160
120
80
40
100
50
0
-50
Unpaired median difference
Original
N = 100
Mean across Simulations
N = 100
Mean across Simulations
minus Original
