# Supplementary material for: Sex- and age-specific development of ssRNA virus receptor expression in the human brain
Source: PLoS One. 2026 Oct 29;21(10):e0360526. doi: 10.1371/journal.pone.0360526 (PMC13718946; doi:10.1371/journal.pone.0360526)
Supplement: Supplementary file 3 [file pone.0360526.s003.pdf]

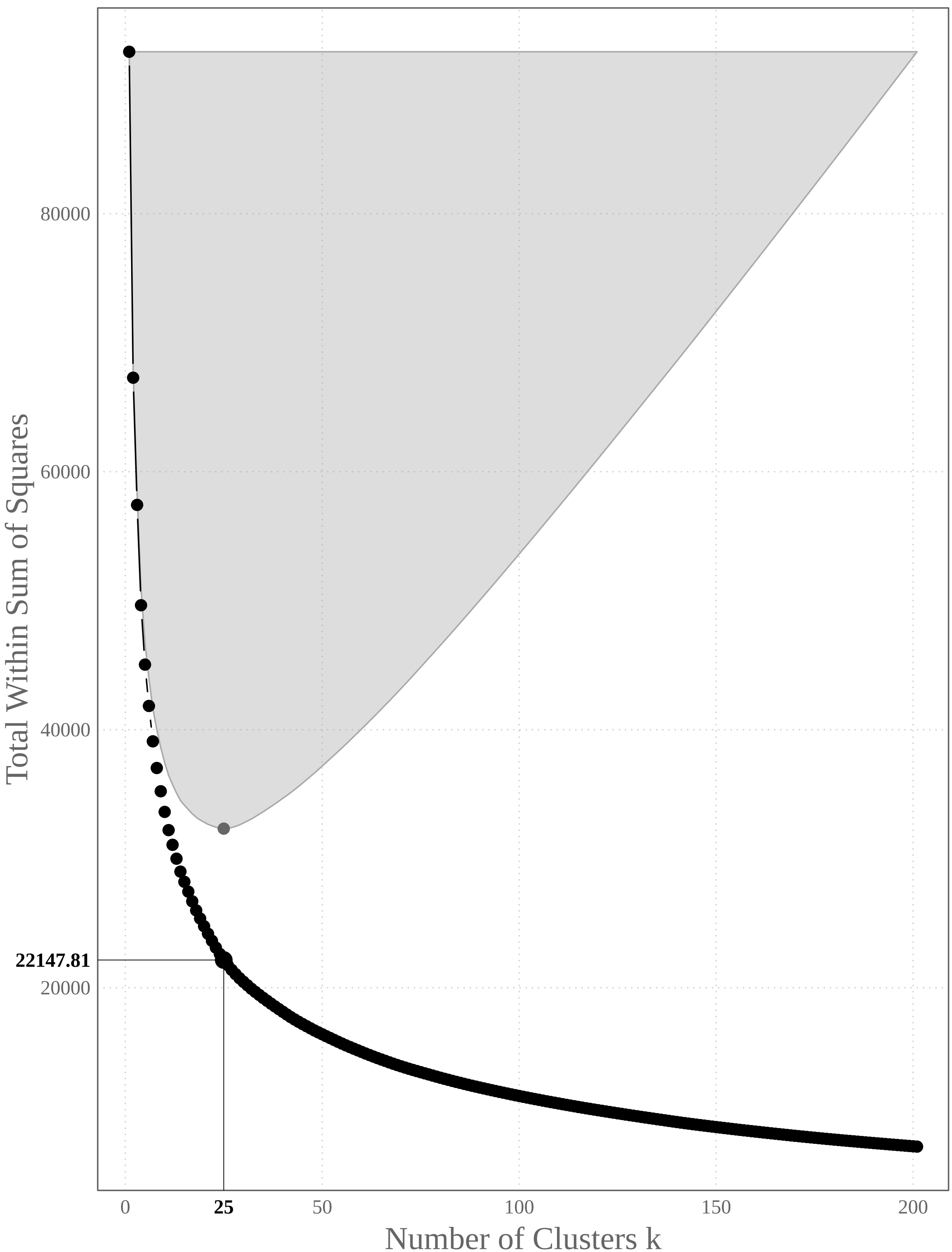

Total Within Sum of Squares
80000
60000
40000
22147.81
20000
0
25
50
100
150
200
Number of Clusters k
