# Supplementary material for: Sex- and age-specific development of ssRNA virus receptor expression in the human brain
Source: PLoS One. 2026 Oct 29;21(10):e0360526. doi: 10.1371/journal.pone.0360526 (PMC13718946; doi:10.1371/journal.pone.0360526)

Selected examples of Between−cluster estimation statistics for clustering on 11 cortical areas

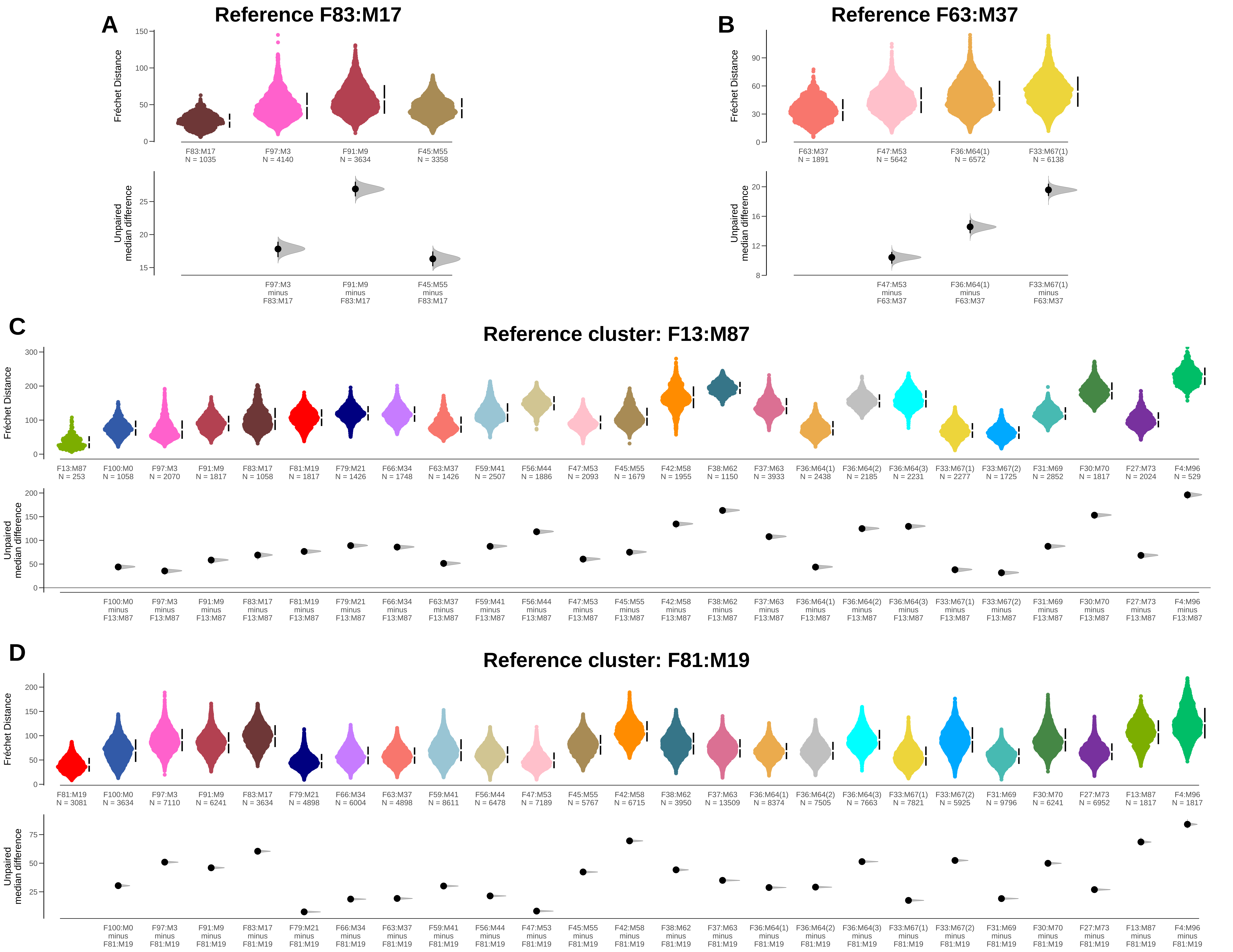

Supplement: Supplementary file 4 [file pone.0360526.s004.pdf]
