# Supplementary material for: Sex- and age-specific development of ssRNA virus receptor expression in the human brain
Source: PLoS One. 2026 Oct 29;21(10):e0360526. doi: 10.1371/journal.pone.0360526 (PMC13718946; doi:10.1371/journal.pone.0360526)
Supplement: Supplementary file 5 [file pone.0360526.s005.pdf]

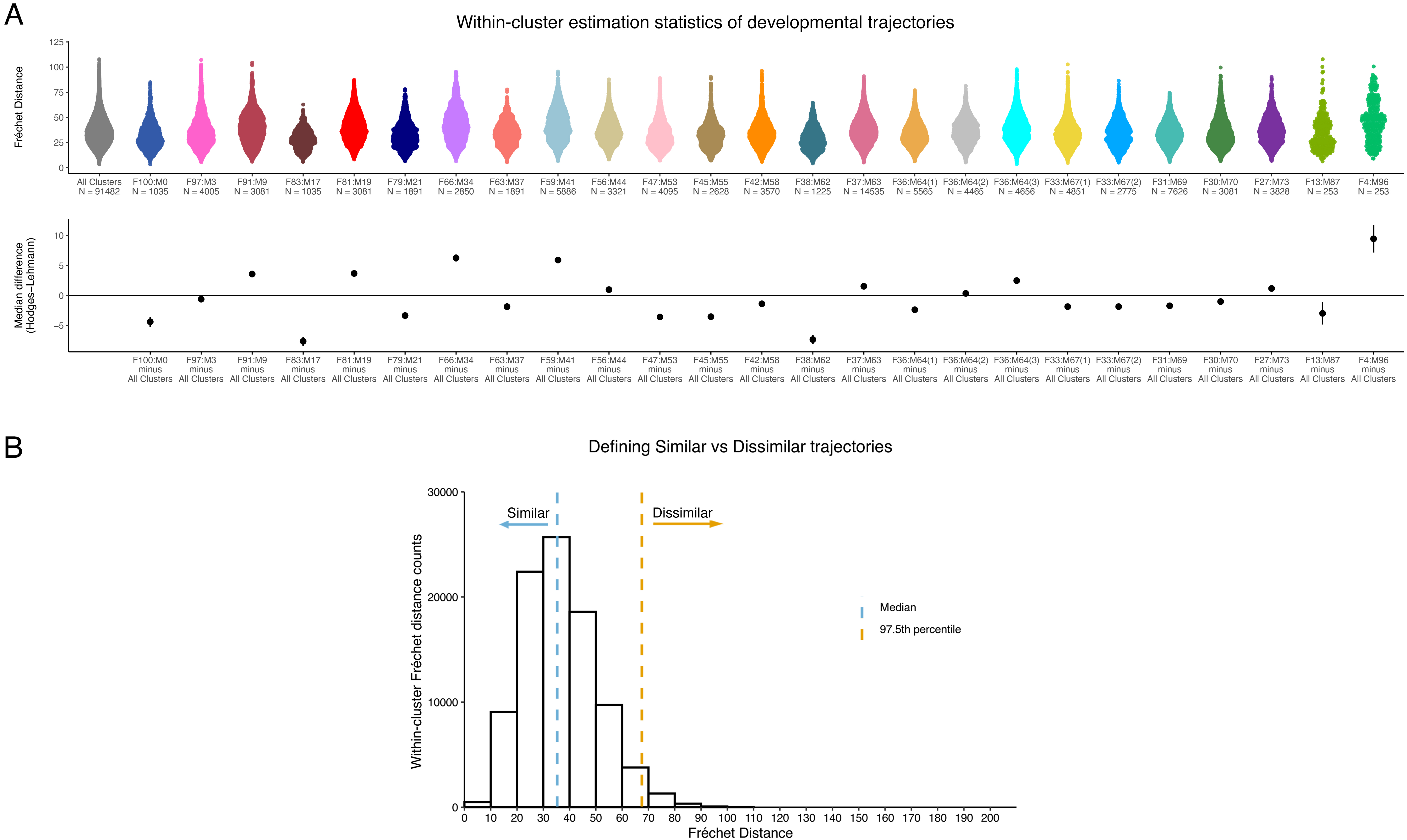
A
Within-cluster estimation statistics of developmental trajectories
Fréchet Distance
All Clusters N = 91482
F100:M0 N = 1035
F97:M3 N = 4005
F91:M9 N = 3081
F83:M17 N = 1035
F81:M19 N = 3081
F79:M21 N = 1891
F66:M34 N = 2850
F63:M37 N = 1891
F59:M41 N = 5886
F56:M44 N = 3321
F47:M53 N = 4095
F45:M55 N = 2628
F42:M58 N = 3570
F38:M62 N = 1225
F37:M63 N = 14535
F36:M64(1) N = 5565
F36:M64(2) N = 4465
F36:M64(3) N = 4656
F33:M67(1) N = 4851
F33:M67(2) N = 2775
F31:M69 N = 7626
F30:M70 N = 3081
F27:M73 N = 3828
F13:M87 N = 253
F4:M96 N = 253
Median difference (Hodges–Lehmann)
F100:M0 minus All Clusters
F97:M3 minus All Clusters
F91:M9 minus All Clusters
F83:M17 minus All Clusters
F81:M19 minus All Clusters
F79:M21 minus All Clusters
F66:M34 minus All Clusters
F63:M37 minus All Clusters
F59:M41 minus All Clusters
F56:M44 minus All Clusters
F47:M53 minus All Clusters
F45:M55 minus All Clusters
F42:M58 minus All Clusters
F38:M62 minus All Clusters
F37:M63 minus All Clusters
F36:M64(1) minus All Clusters
F36:M64(2) minus All Clusters
F36:M64(3) minus All Clusters
F33:M67(1) minus All Clusters
F33:M67(2) minus All Clusters
F31:M69 minus All Clusters
F30:M70 minus All Clusters
F27:M73 minus All Clusters
F13:M87 minus All Clusters
F4:M96 minus All Clusters
B
Defining Similar vs Dissimilar trajectories
Similar
Dissimilar
Within-cluster Fréchet distance counts
Median
97.5th percentile
Fréchet Distance
