# Supplementary material for: Sex- and age-specific development of ssRNA virus receptor expression in the human brain
Source: PLoS One. 2026 Oct 29;21(10):e0360526. doi: 10.1371/journal.pone.0360526 (PMC13718946; doi:10.1371/journal.pone.0360526)
Supplement: Supplementary file 6 [file pone.0360526.s006.pdf]

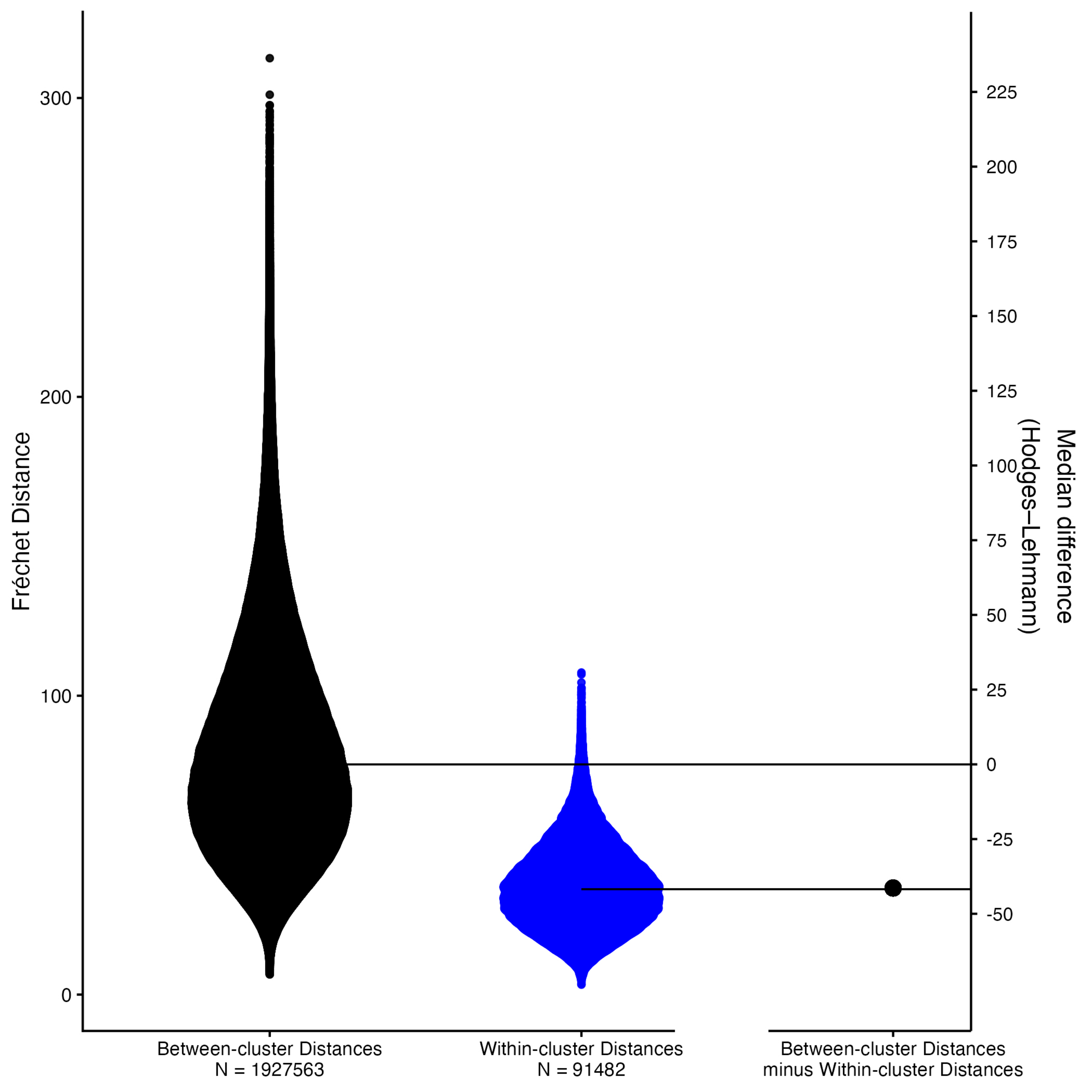
Fréchet Distance
300
200
100
0
Median difference
(Hodges–Lehmann)
225
200
175
150
125
100
75
50
25
0
-25
-50
Between-cluster Distances
N = 1927563
Within-cluster Distances
N = 91482
Between-cluster Distances
minus Within-cluster Distances
