# Supplementary material for: Sex- and age-specific development of ssRNA virus receptor expression in the human brain
Source: PLoS One. 2026 Oct 29;21(10):e0360526. doi: 10.1371/journal.pone.0360526 (PMC13718946; doi:10.1371/journal.pone.0360526)
Supplement: Supplementary file 7 [file pone.0360526.s007.pdf]

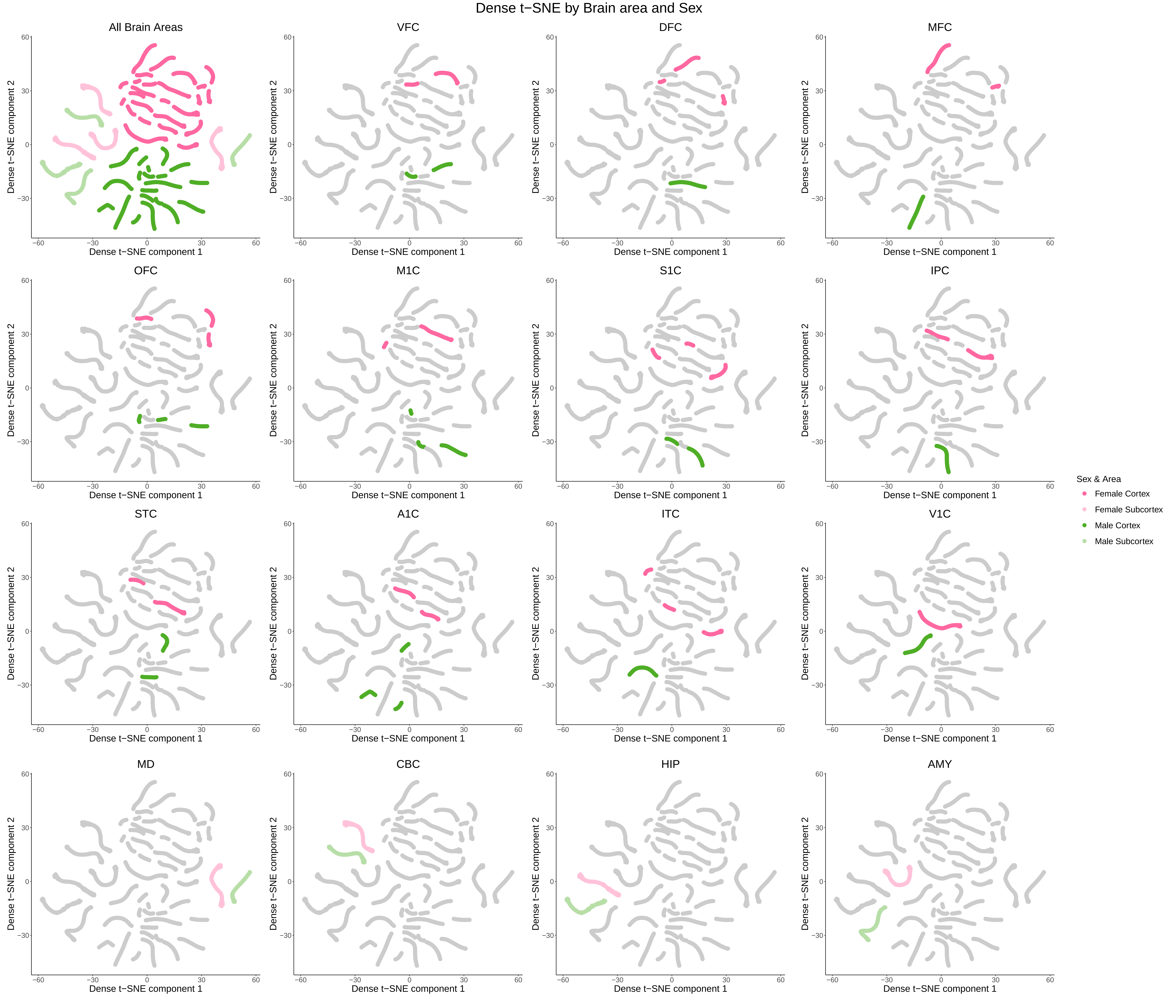
Dense t−SNE by Brain area and Sex
All Brain Areas
VFC
DFC
MFC
OFC
M1C
S1C
IPC
STC
A1C
ITC
V1C
MD
CBC
HIP
AMY
Dense t−SNE component 1
Dense t−SNE component 2
−60
−30
0
30
60
Sex & Area
Female Cortex
Female Subcortex
Male Cortex
Male Subcortex
