# Supplementary material for: Sex- and age-specific development of ssRNA virus receptor expression in the human brain
Source: PLoS One. 2026 Oct 29;21(10):e0360526. doi: 10.1371/journal.pone.0360526 (PMC13718946; doi:10.1371/journal.pone.0360526)
Supplement: Supplementary file 8 [file pone.0360526.s008.pdf]

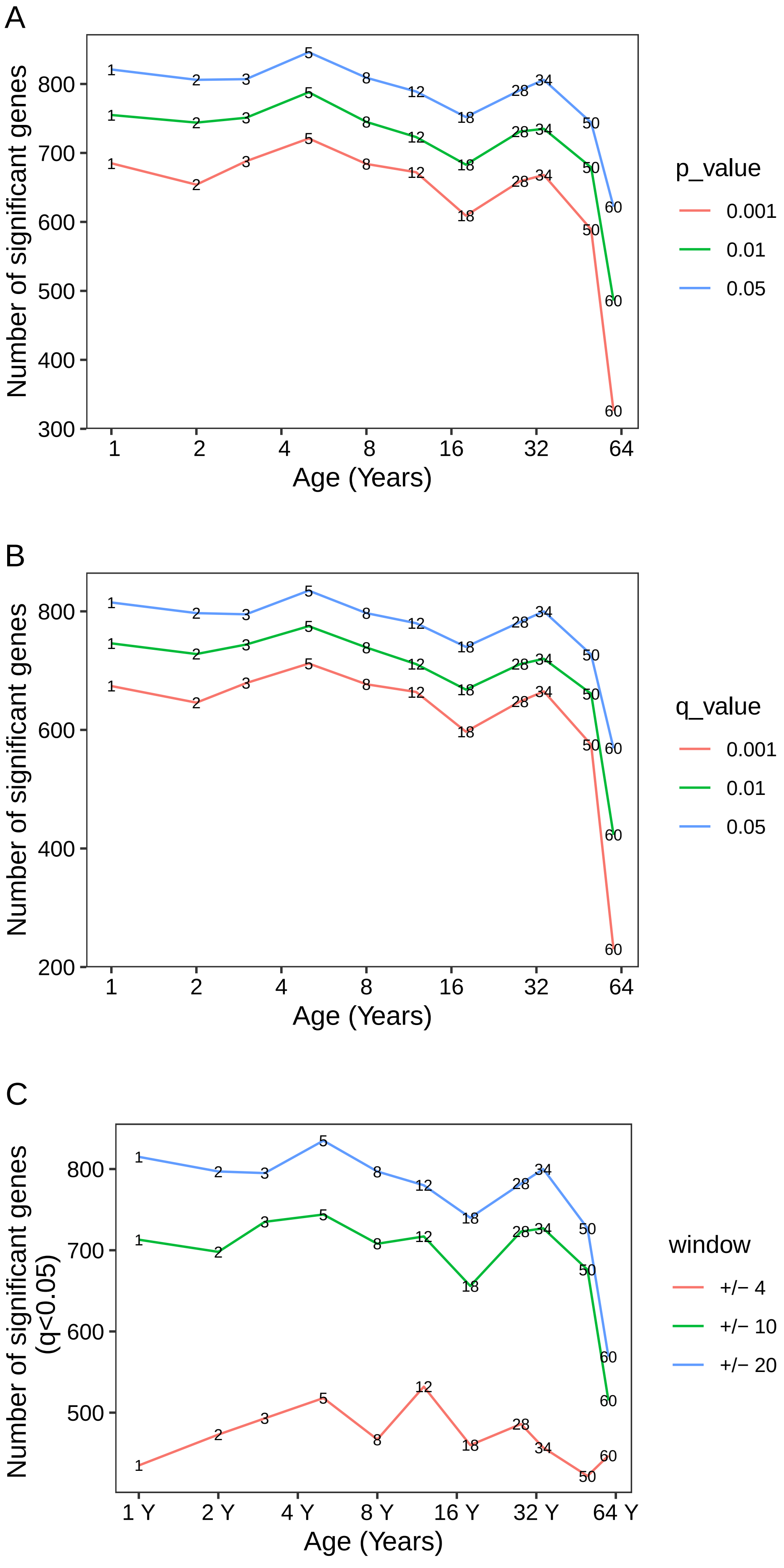
A
Number of significant genes
300
400
500
600
700
800
1
2
4
8
16
32
64
Age (Years)
p_value
0.001
0.01
0.05
B
Number of significant genes
200
400
600
800
1
2
4
8
16
32
64
Age (Years)
q_value
0.001
0.01
0.05
C
Number of significant genes
(q<0.05)
500
600
700
800
1 Y
2 Y
4 Y
8 Y
16 Y
32 Y
64 Y
Age (Years)
window
+/− 4
+/− 10
+/− 20
