# Supplementary material for: Sex- and age-specific development of ssRNA virus receptor expression in the human brain
Source: PLoS One. 2026 Oct 29;21(10):e0360526. doi: 10.1371/journal.pone.0360526 (PMC13718946; doi:10.1371/journal.pone.0360526)
Supplement: Supplementary file 9 [file pone.0360526.s009.pdf]

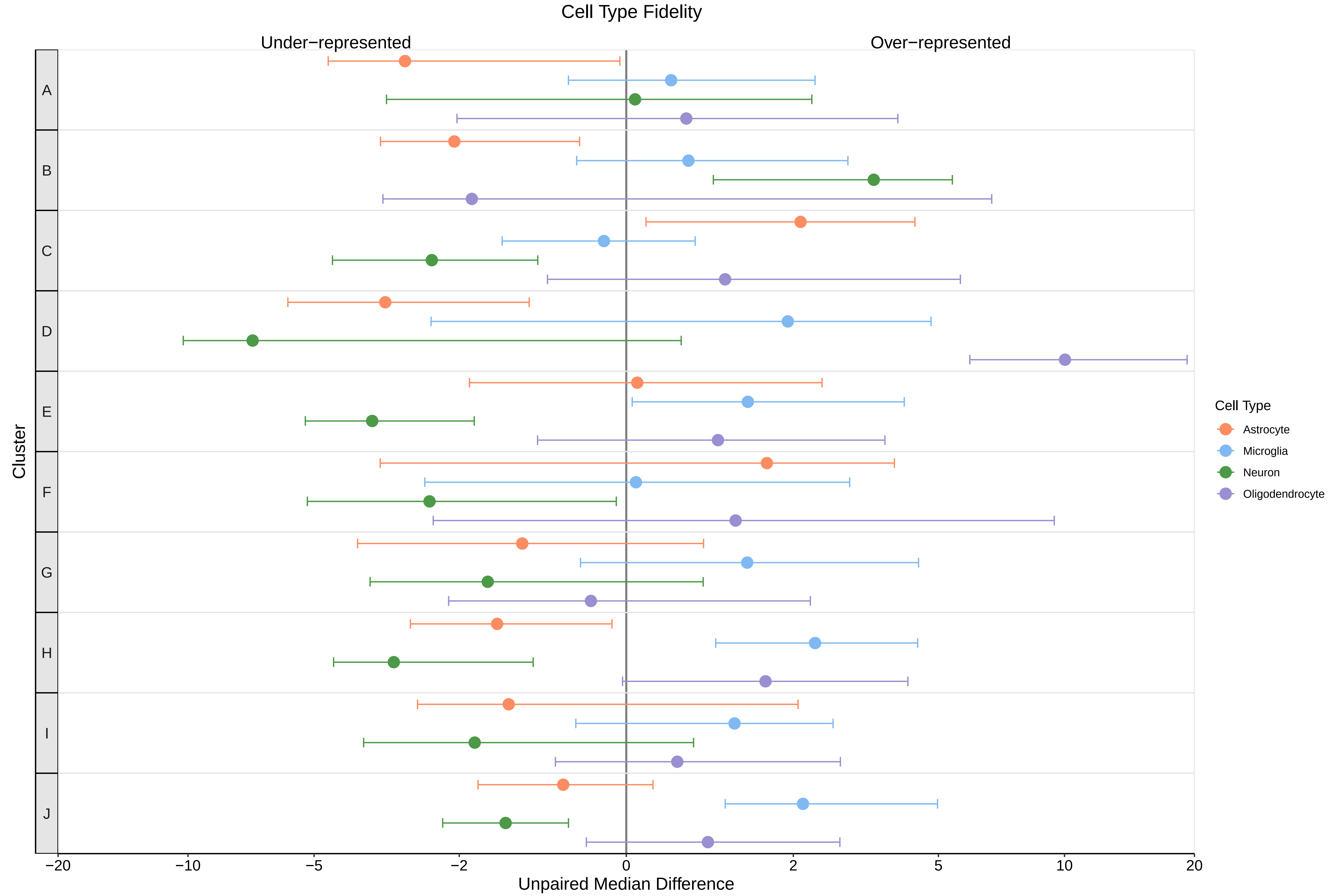

Cell Type Fidelity
Under−represented
Over−represented
Cluster
A
B
C
D
E
F
G
H
I
J
−20
−10
−5
−2
0
2
5
10
20
Unpaired Median Difference
Cell Type
Astrocyte
Microglia
Neuron
Oligodendrocyte
